# Supplementary figures and images for: ATAD2 drives melanoma growth and progression and inhibits ferroptosis
Source: EMBO Rep. 2025 Dec 2;27(2):501–32. doi: 10.1038/s44319-025-00660-w (PMC12852765; doi:10.1038/s44319-025-00660-w)

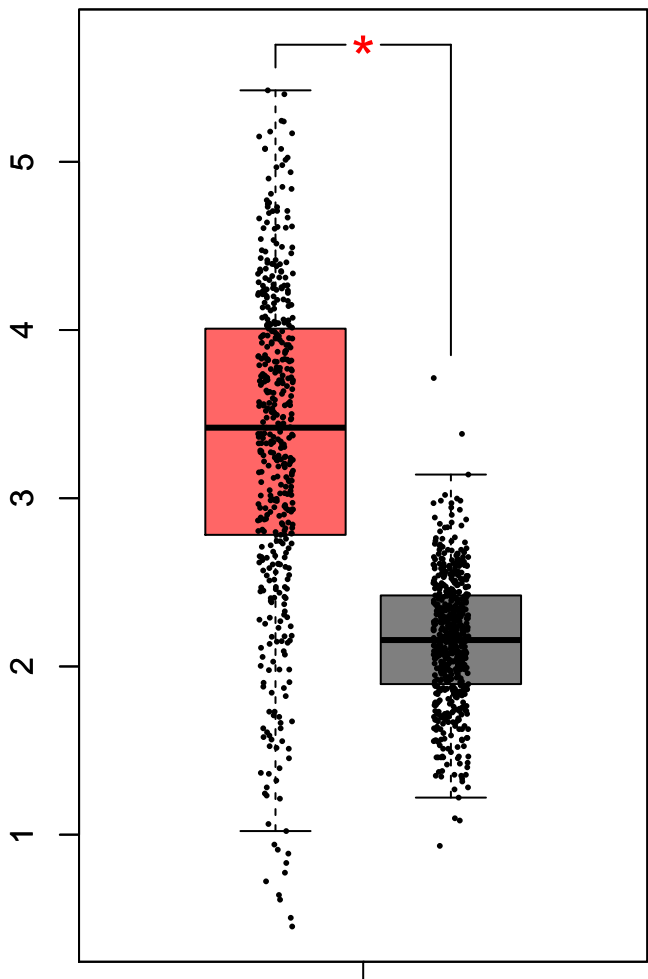

SKCM  
(num(T)=461; num(N)=558)

Supplement: Supplementary file 7 — Source data Fig. 1 [file 44319_2025_660_MOESM7_ESM.zip › Figure 1/A/atad2_boxplot_2EFfP copy.pdf]

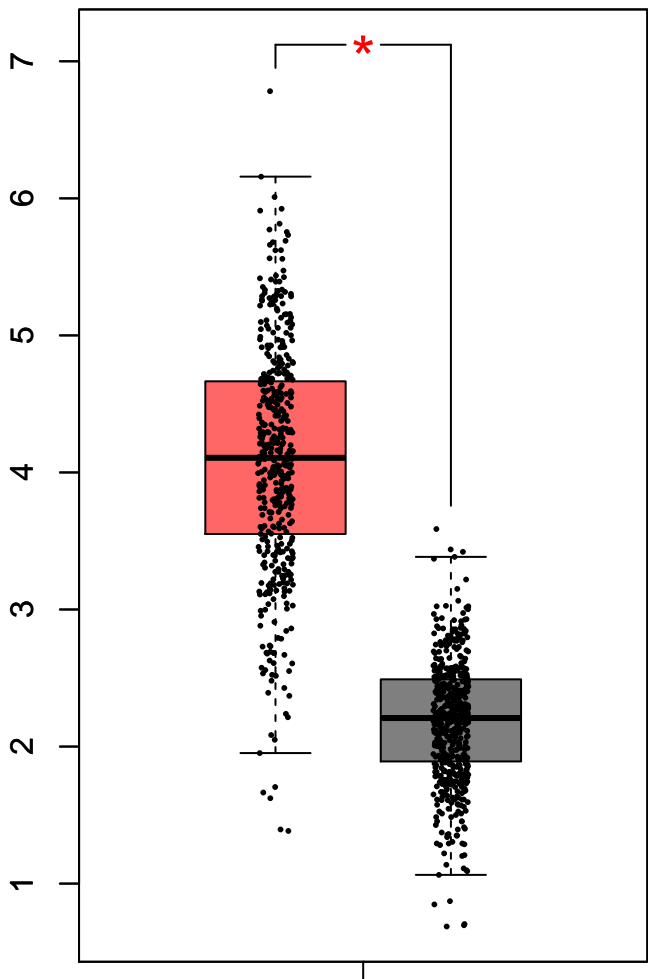

SKCM  
(num(T)=461; num(N)=558)

Supplement: Supplementary file 7 — Source data Fig. 1 [file 44319_2025_660_MOESM7_ESM.zip › Figure 1/F/E2F1_boxplot_bwuQj.pdf]

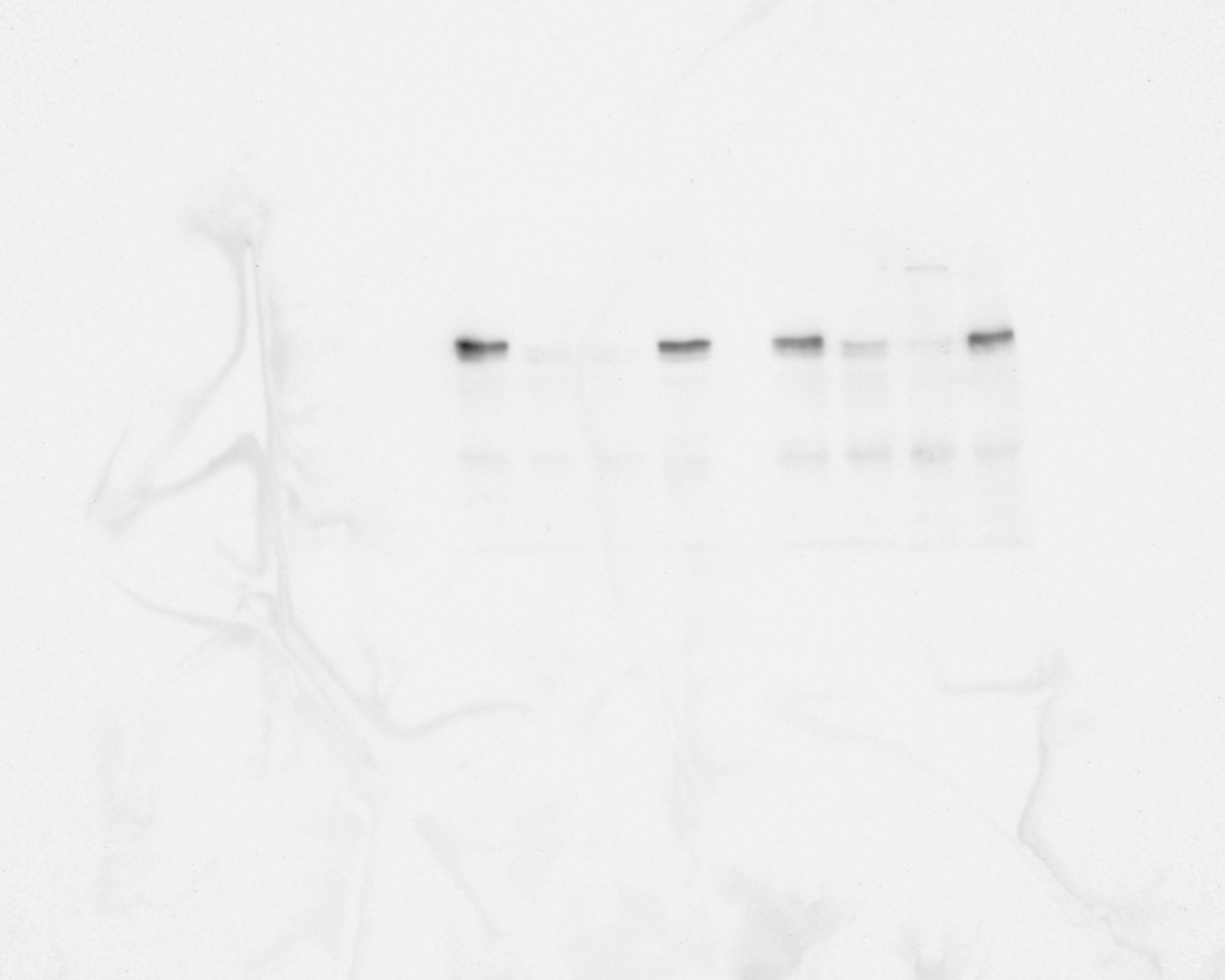

Supplement: Supplementary file 7 — Source data Fig. 1 [file 44319_2025_660_MOESM7_ESM.zip › Figure 1/H/A375_SKMEL-103_E2F1_1(Chemiluminescence).tif]

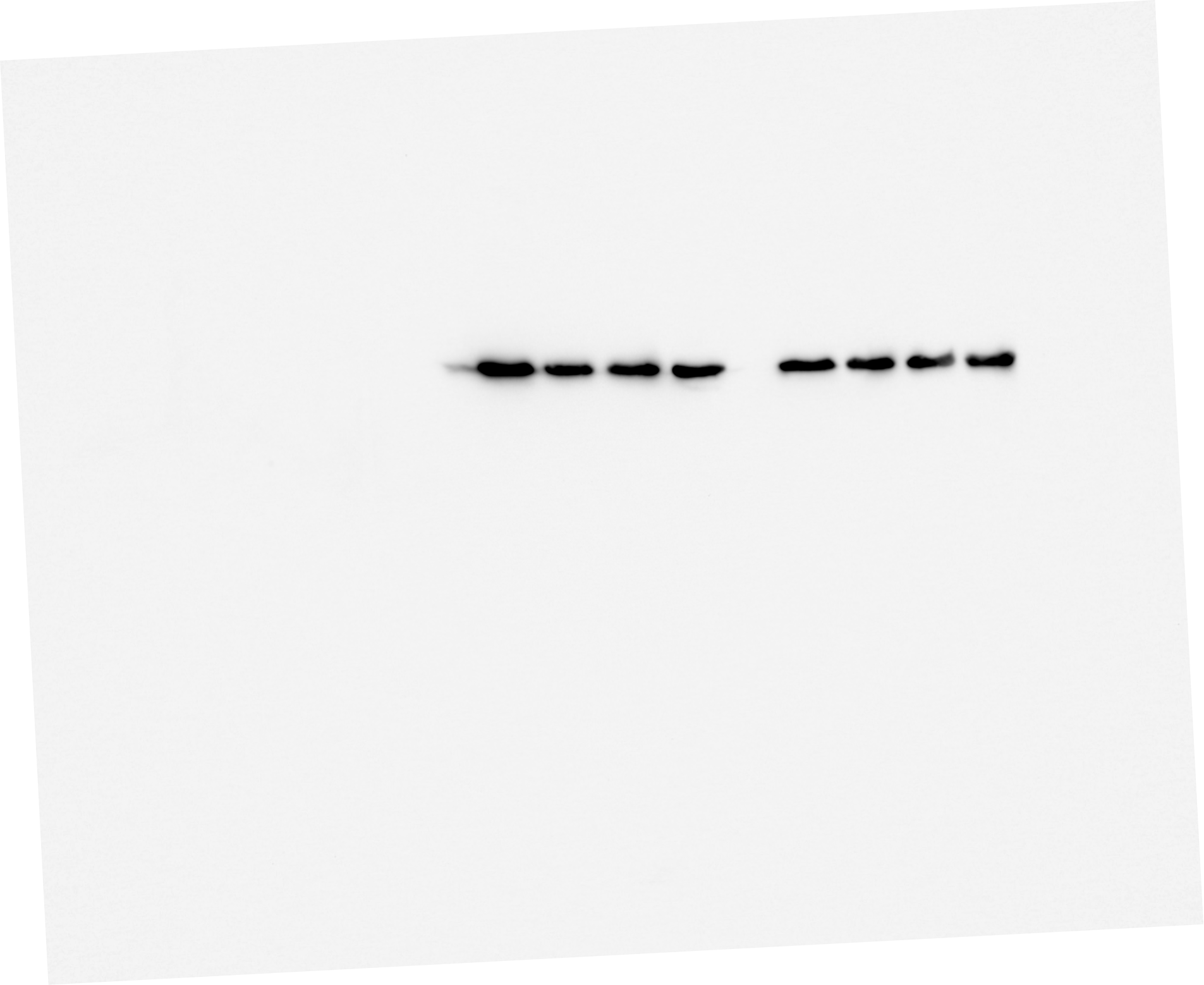

Supplement: Supplementary file 7 — Source data Fig. 1 [file 44319_2025_660_MOESM7_ESM.zip › Figure 1/H/A375_SKMEL-103_E2F1_actin_2(Chemiluminescence).tif]

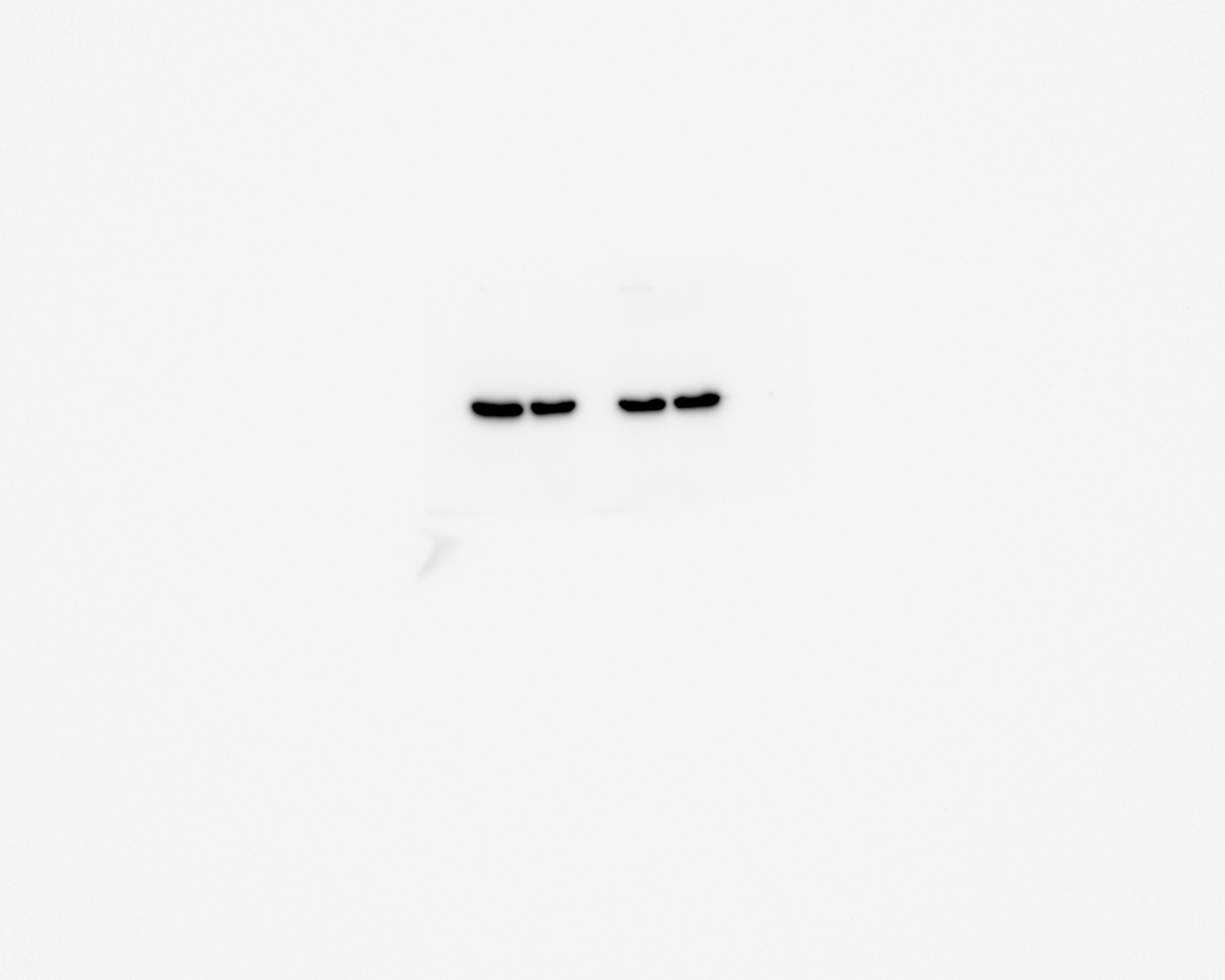

Supplement: Supplementary file 7 — Source data Fig. 1 [file 44319_2025_660_MOESM7_ESM.zip › Figure 1/J/Actin for ATAD2 protein level in E2F1 KD cells.tif]

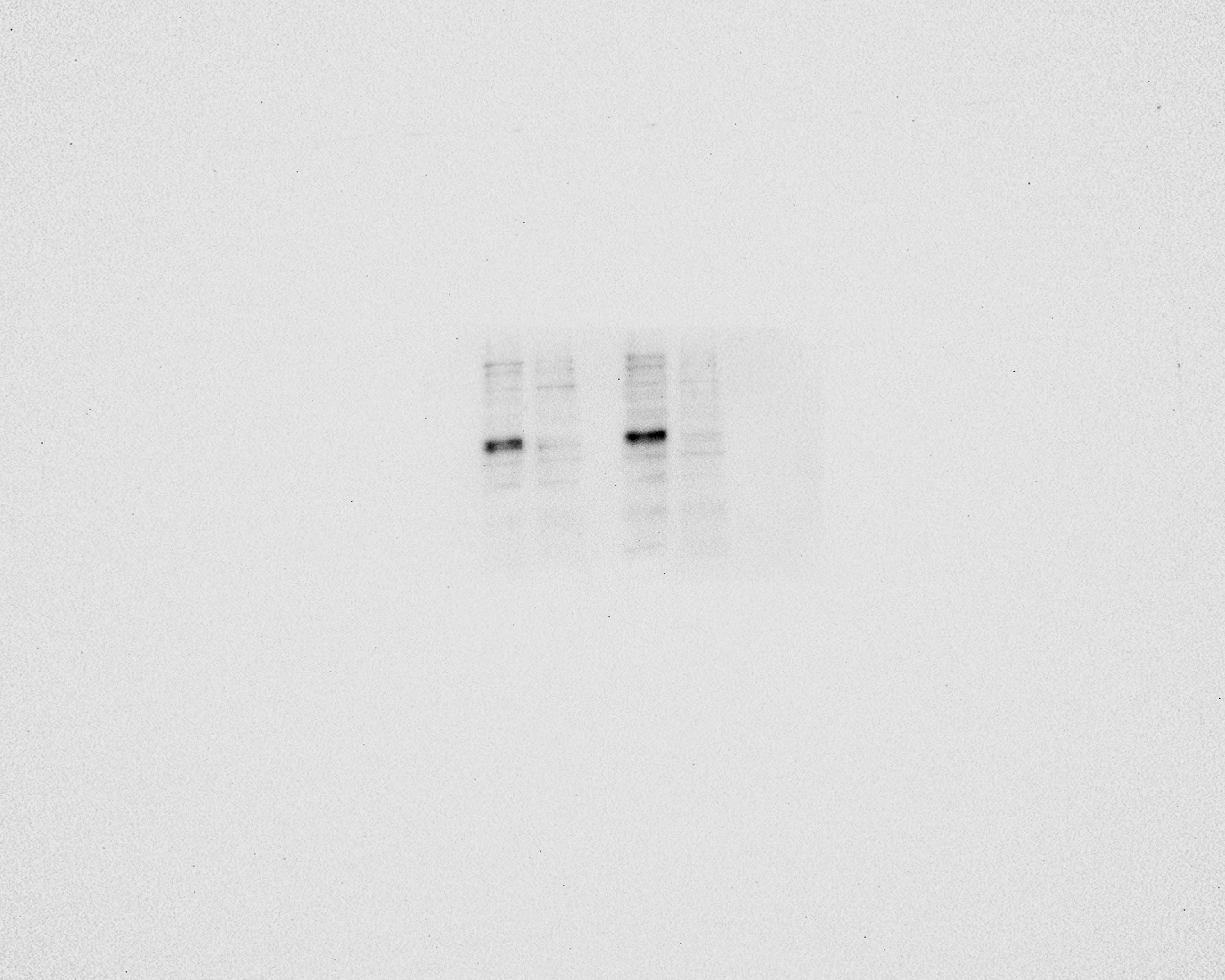

Supplement: Supplementary file 7 — Source data Fig. 1 [file 44319_2025_660_MOESM7_ESM.zip › Figure 1/J/E2F1 protein level in E2F1 KD cells.tif]

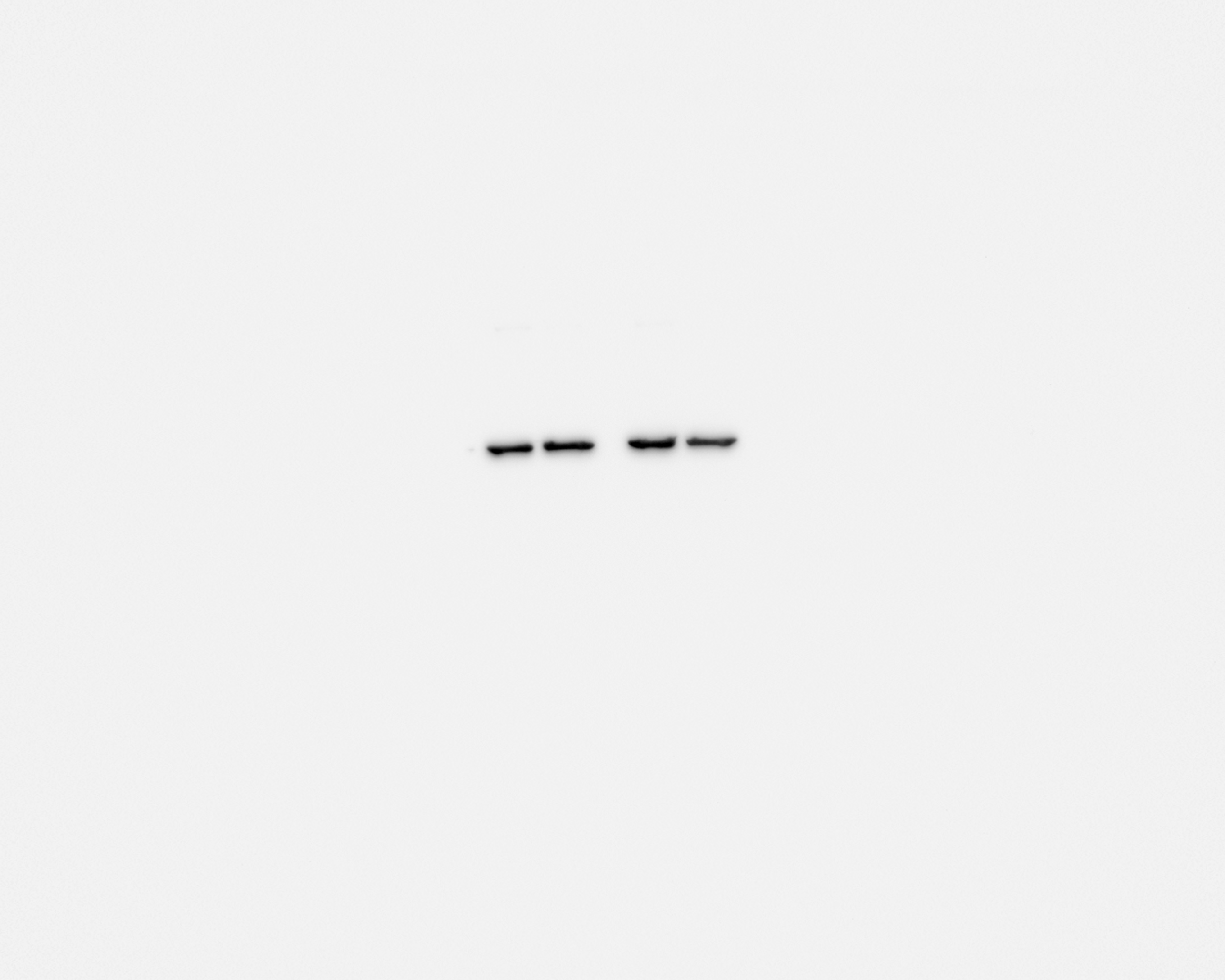

Supplement: Supplementary file 7 — Source data Fig. 1 [file 44319_2025_660_MOESM7_ESM.zip › Figure 1/J/Actin for E2F1 protein level in E2F1 KD cells.tif]

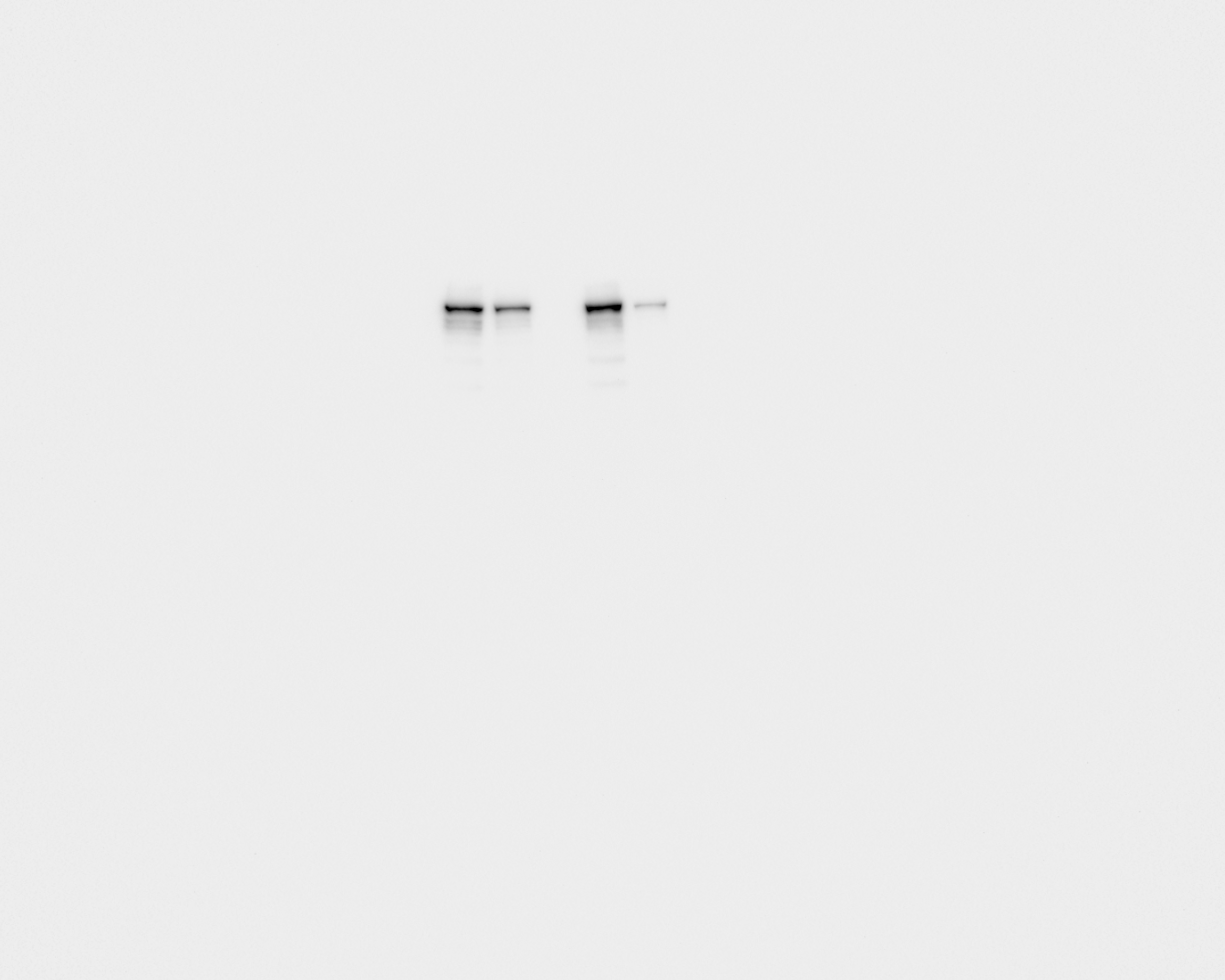

Supplement: Supplementary file 7 — Source data Fig. 1 [file 44319_2025_660_MOESM7_ESM.zip › Figure 1/J/ATAD2 protein level in E2F1 KD cells.tif]

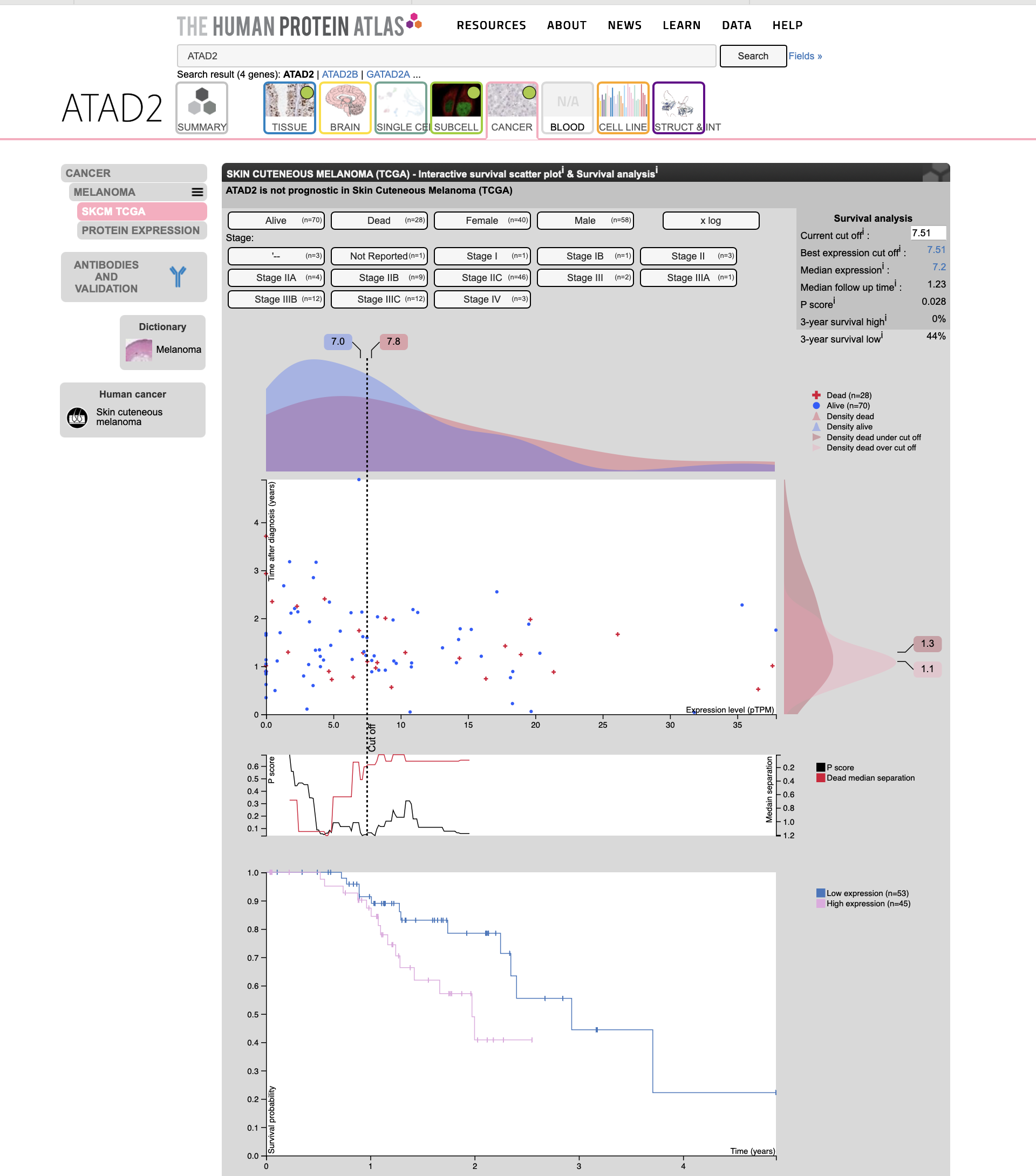

Supplement: Supplementary file 7 — Source data Fig. 1 [file 44319_2025_660_MOESM7_ESM.zip › Figure 1/C/Screenshot 2025-07-14 at 8.55.48ΓÇ»AM.png]

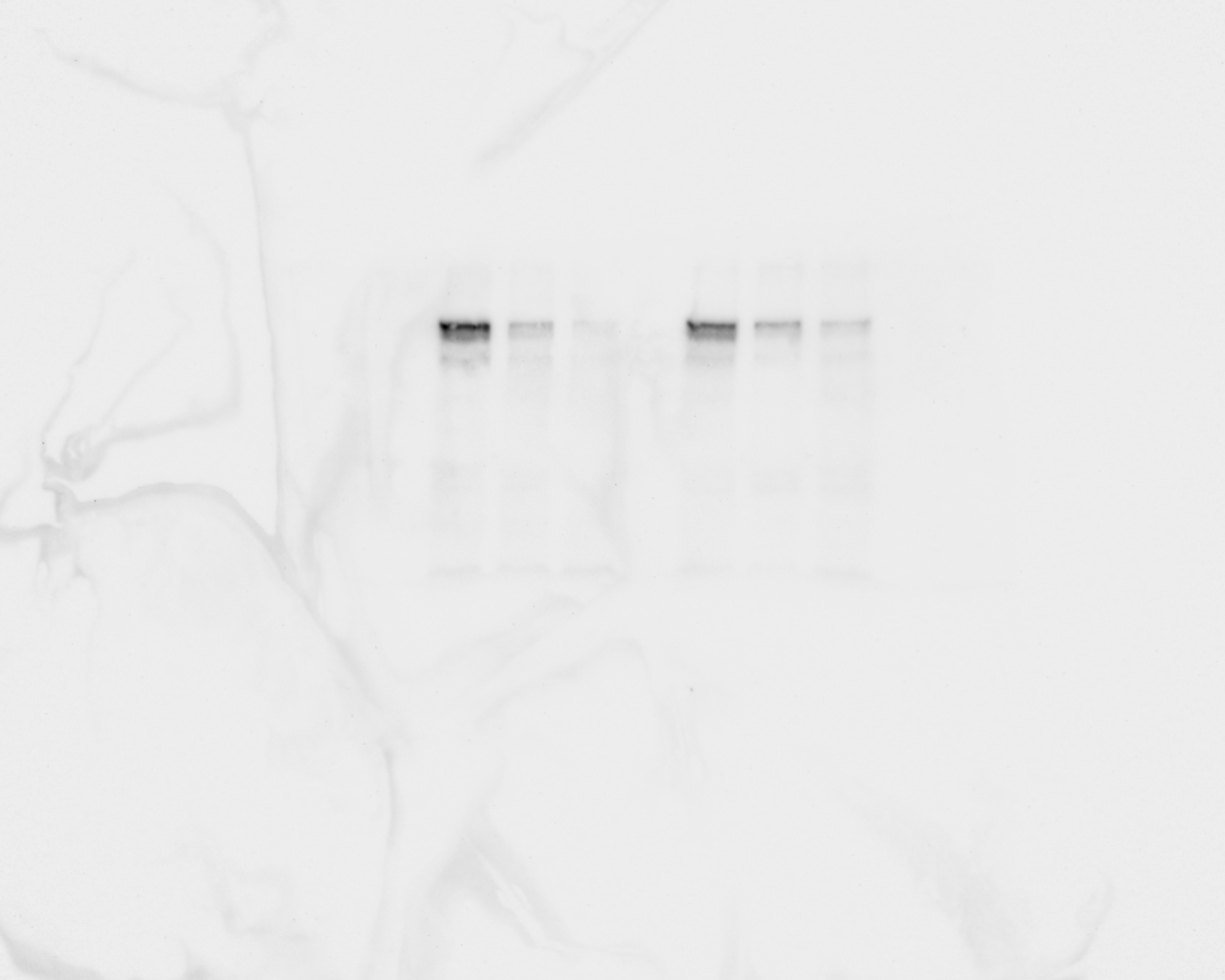

Supplement: Supplementary file 7 — Source data Fig. 1 [file 44319_2025_660_MOESM7_ESM.zip › Figure 1/E/SKMEL-2 and SKMEL-103_ATAD2(Chemiluminescence).tif]

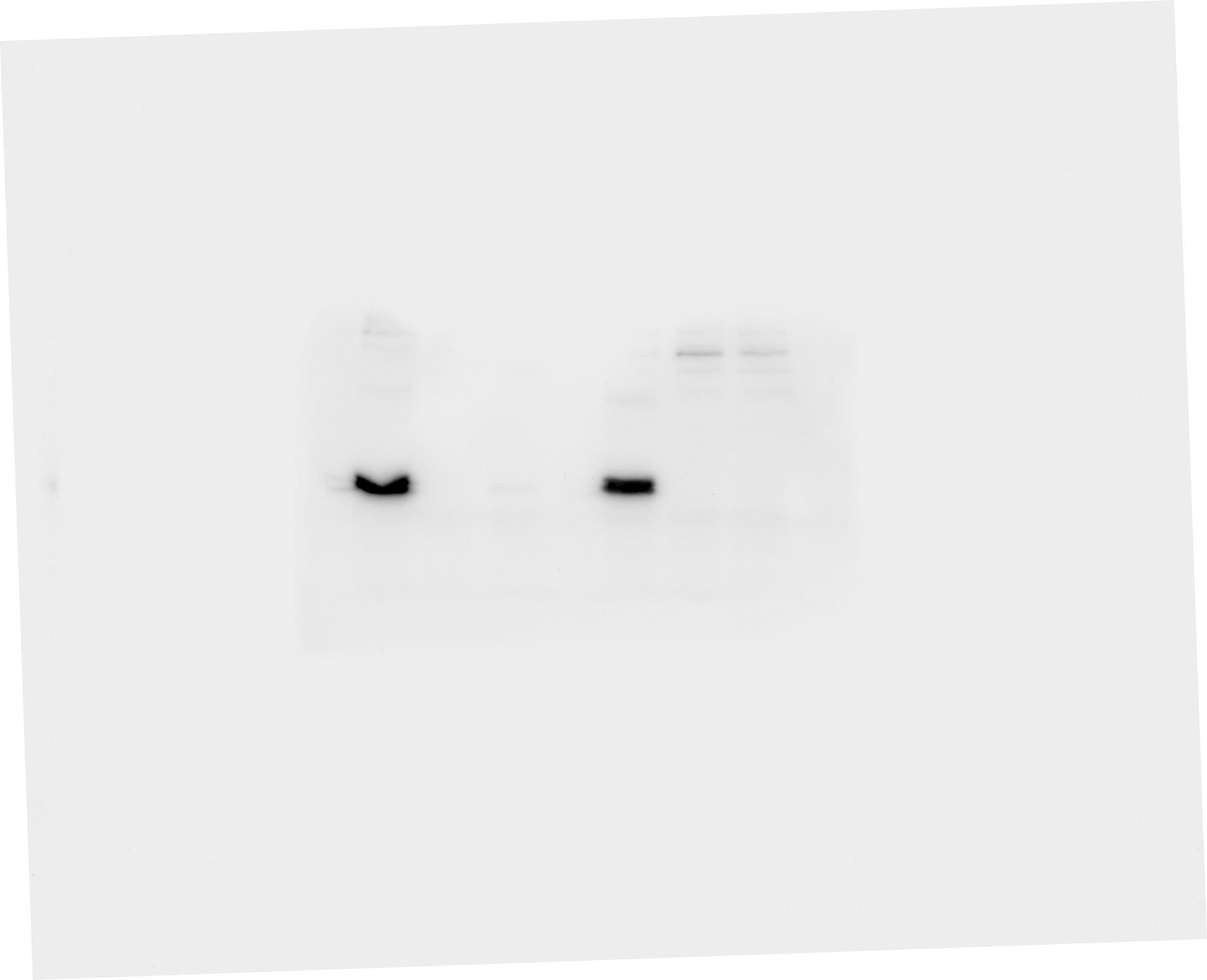

Supplement: Supplementary file 7 — Source data Fig. 1 [file 44319_2025_660_MOESM7_ESM.zip › Figure 1/E/SKMEL-2 and SKMEL-103_perk_1(Chemiluminescence).tif]

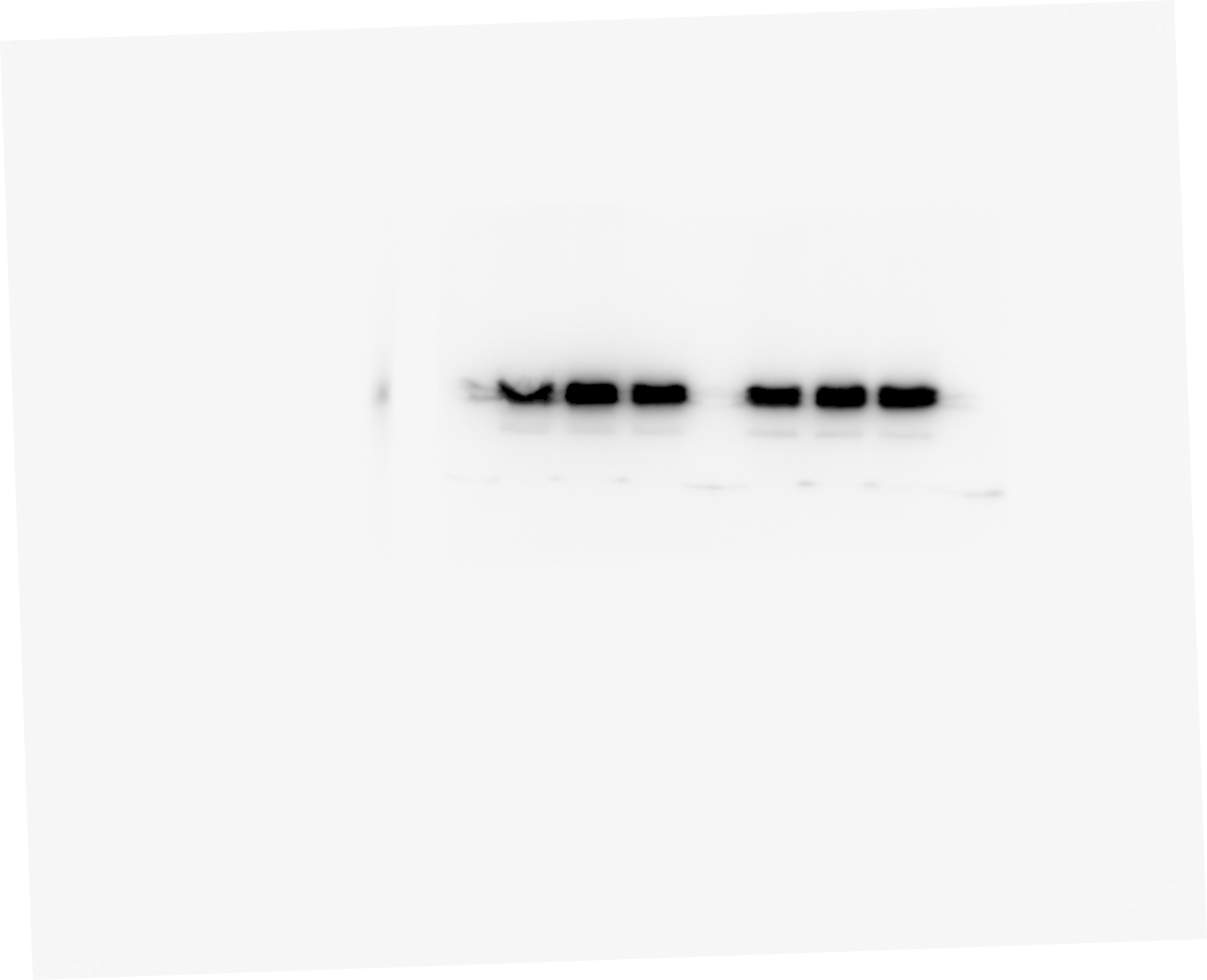

Supplement: Supplementary file 7 — Source data Fig. 1 [file 44319_2025_660_MOESM7_ESM.zip › Figure 1/E/SKMEL-2 and SKMEL-103_terk_2(Chemiluminescence).tif]

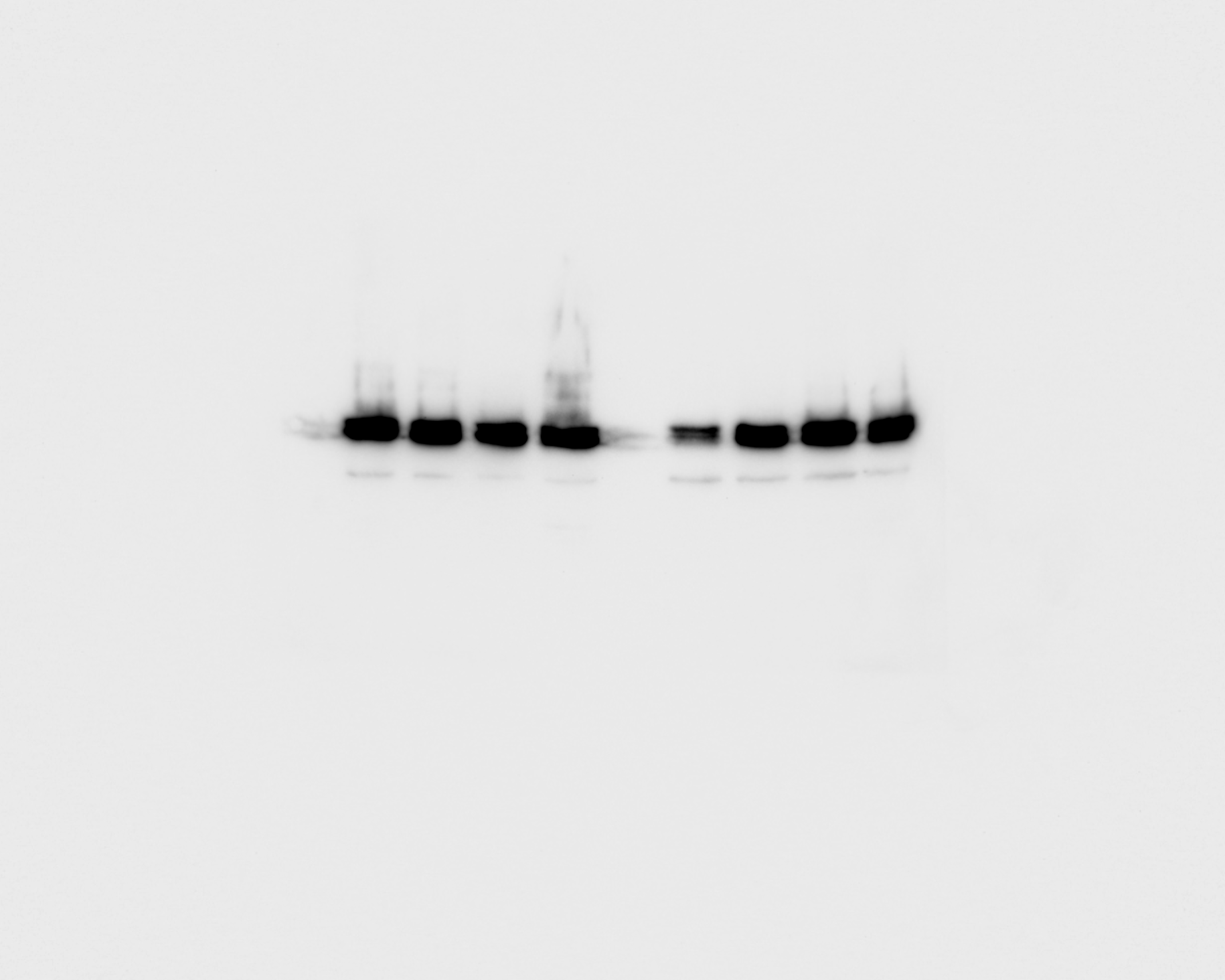

Supplement: Supplementary file 7 — Source data Fig. 1 [file 44319_2025_660_MOESM7_ESM.zip › Figure 1/E/A375_M14_terk_2(Chemiluminescence).tif]

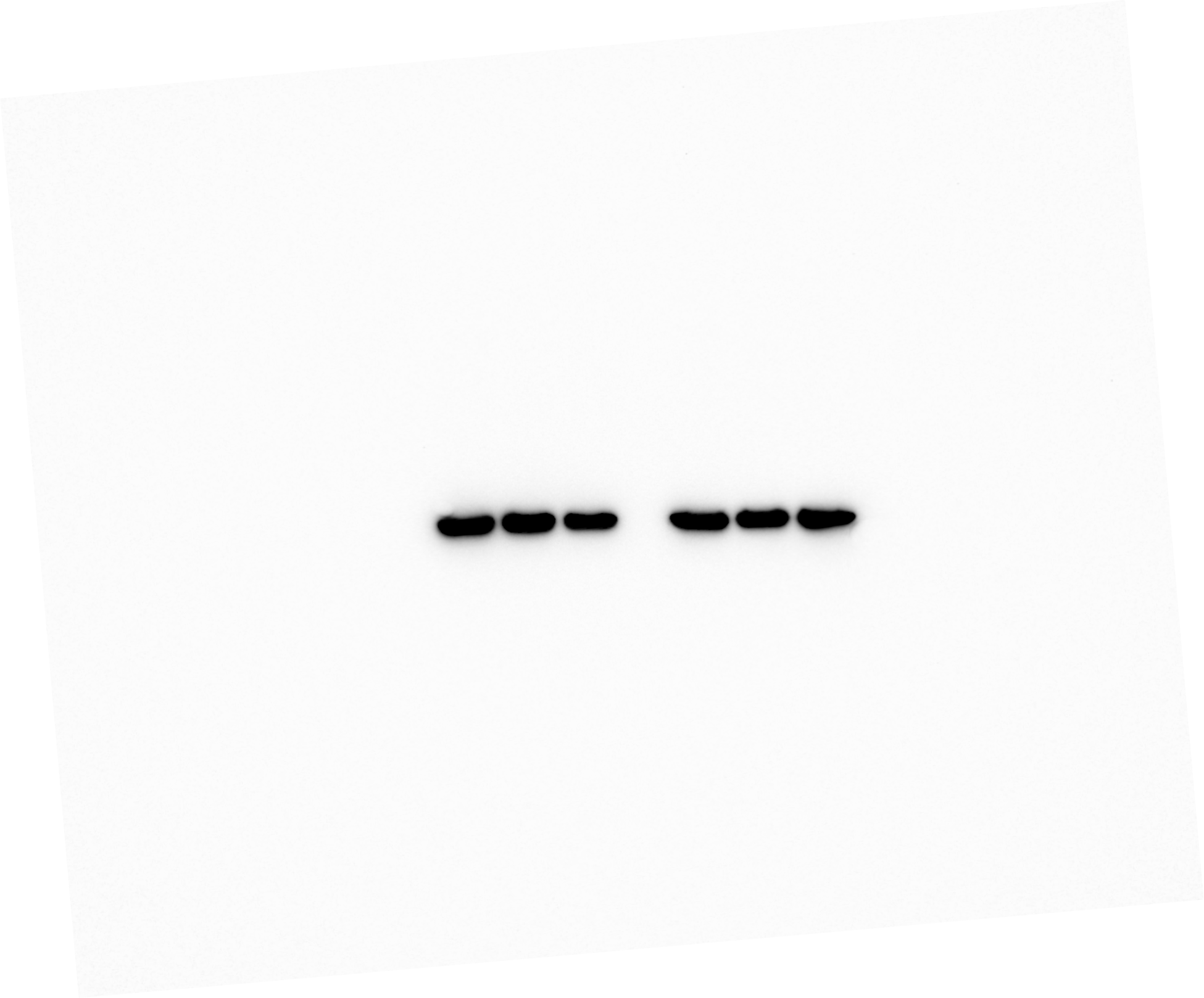

Supplement: Supplementary file 7 — Source data Fig. 1 [file 44319_2025_660_MOESM7_ESM.zip › Figure 1/E/SKMEL-2 and SKMEL-103_ATAD2_actin(Chemiluminescence).tif]

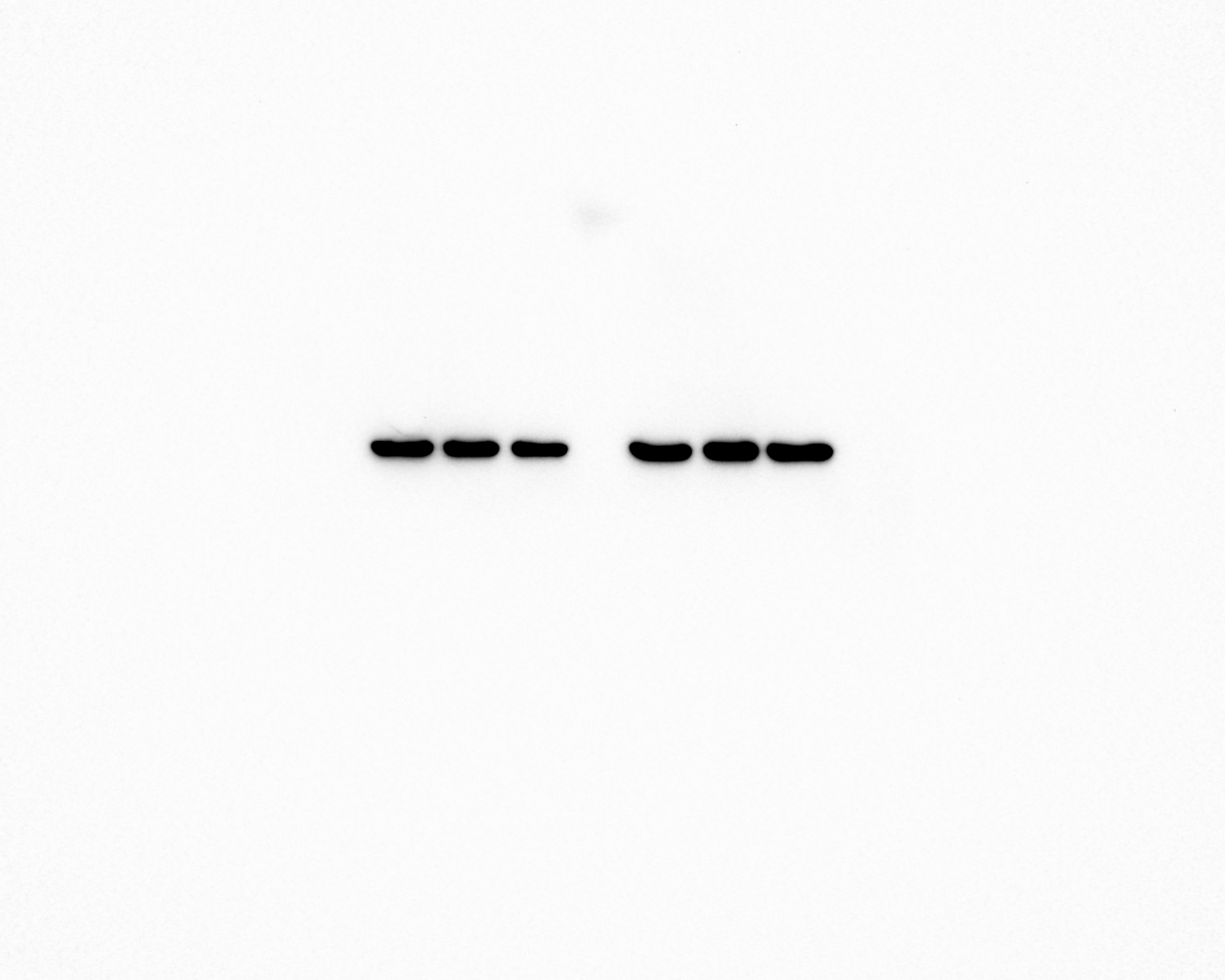

Supplement: Supplementary file 7 — Source data Fig. 1 [file 44319_2025_660_MOESM7_ESM.zip › Figure 1/E/A375_M14_ATAD2_actin(Chemiluminescence).tif]

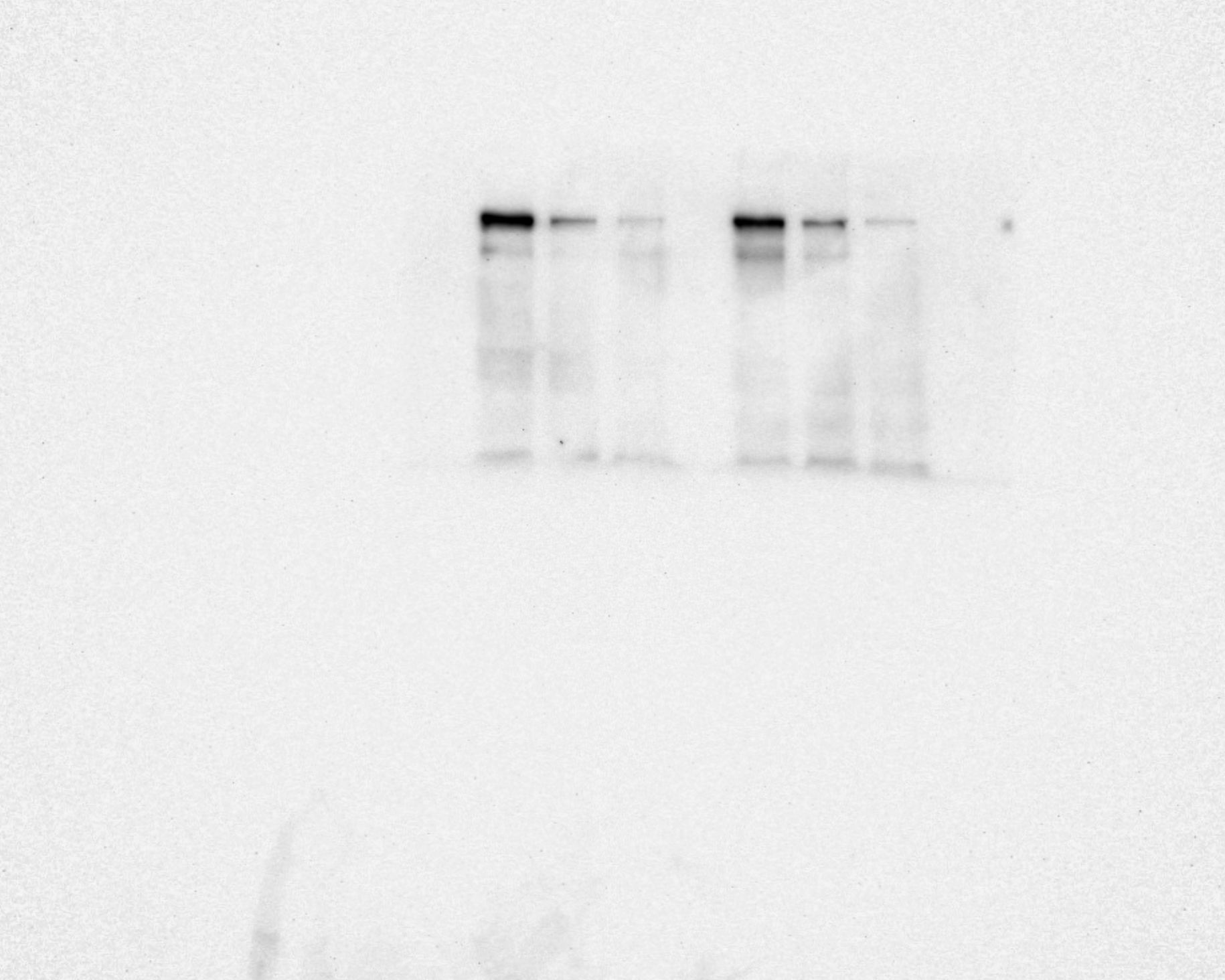

Supplement: Supplementary file 7 — Source data Fig. 1 [file 44319_2025_660_MOESM7_ESM.zip › Figure 1/E/A375_M14_ATAD2(Chemiluminescence).tif]

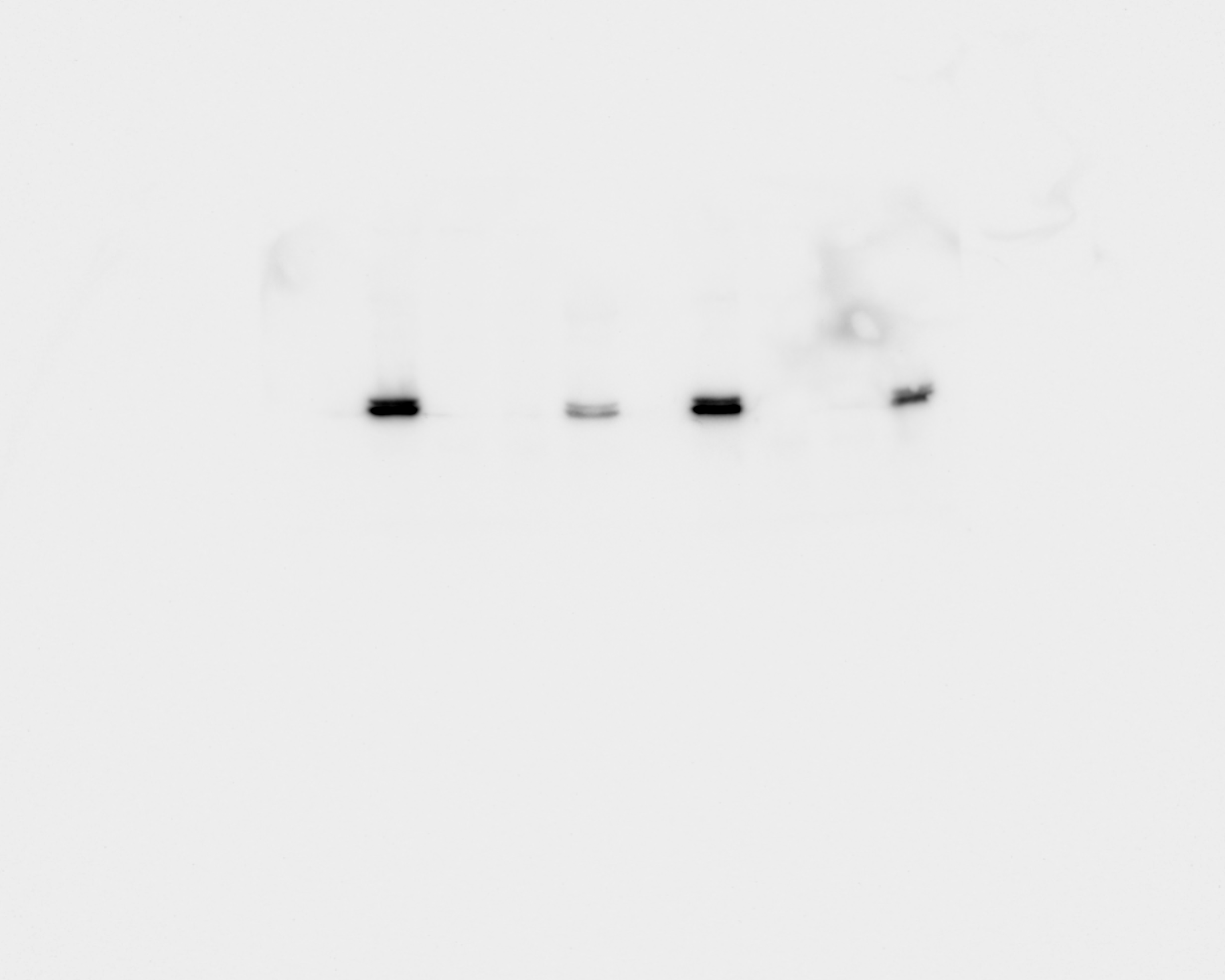

Supplement: Supplementary file 7 — Source data Fig. 1 [file 44319_2025_660_MOESM7_ESM.zip › Figure 1/E/A375_M14_perk_1(Chemiluminescence).tif]

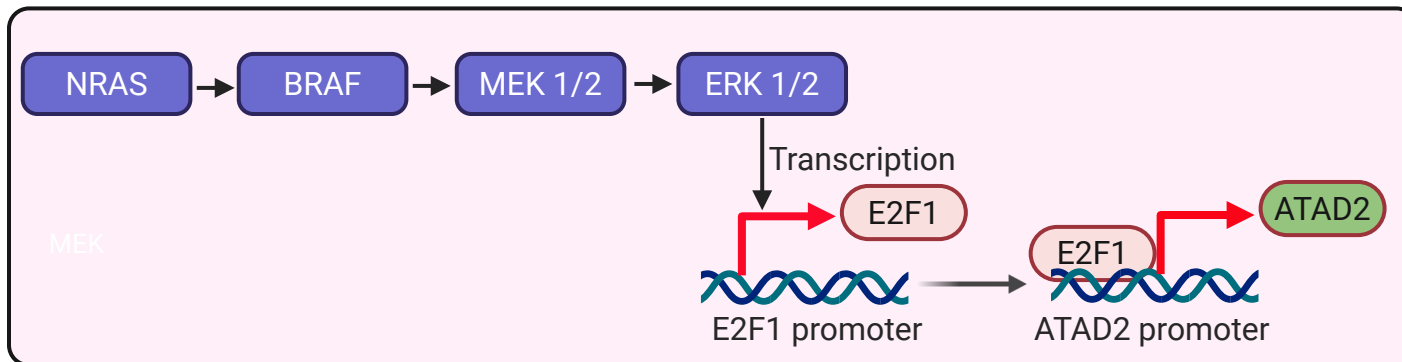

Supplement: Supplementary file 7 — Source data Fig. 1 [file 44319_2025_660_MOESM7_ESM.zip › Figure 1/L/Schematics.pdf]

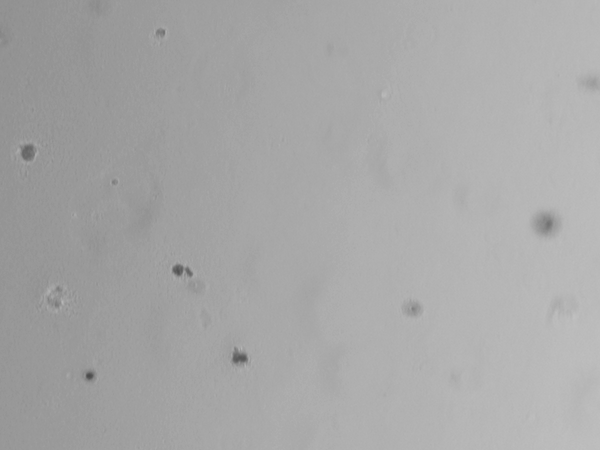

Supplement: Supplementary file 8 — Source data Fig. 2 [file 44319_2025_660_MOESM8_ESM.zip › Figure 2/G/A375-ATAD2-shRNA.tif]

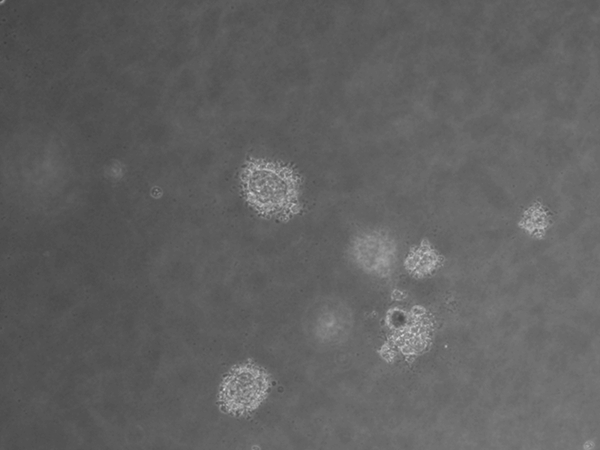

Supplement: Supplementary file 8 — Source data Fig. 2 [file 44319_2025_660_MOESM8_ESM.zip › Figure 2/G/SKMEL-103-NS-shRNA.tif]

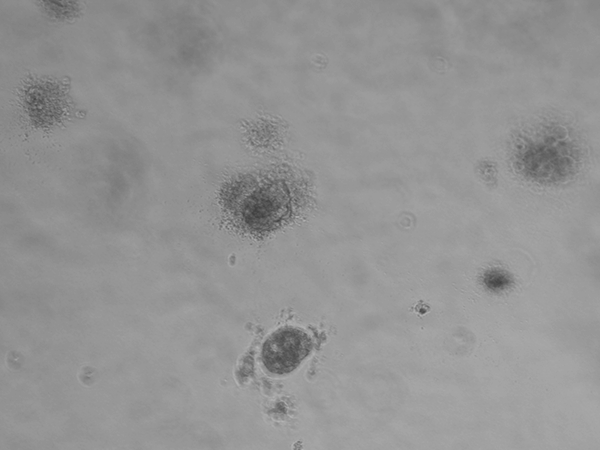

Supplement: Supplementary file 8 — Source data Fig. 2 [file 44319_2025_660_MOESM8_ESM.zip › Figure 2/G/A375-NS-shRNA.tif]

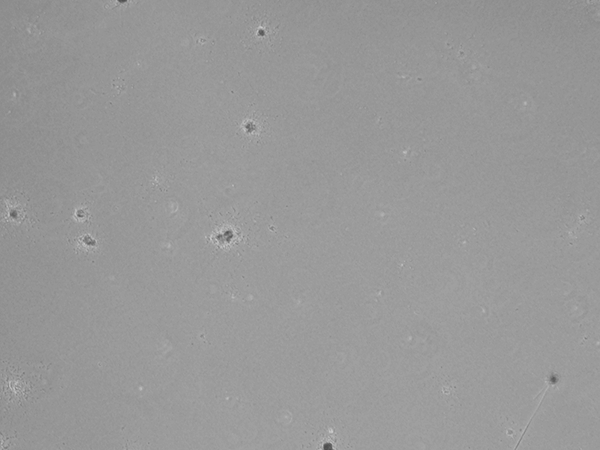

Supplement: Supplementary file 8 — Source data Fig. 2 [file 44319_2025_660_MOESM8_ESM.zip › Figure 2/G/SKMEL-103-ATAD2 shRNA.tif]

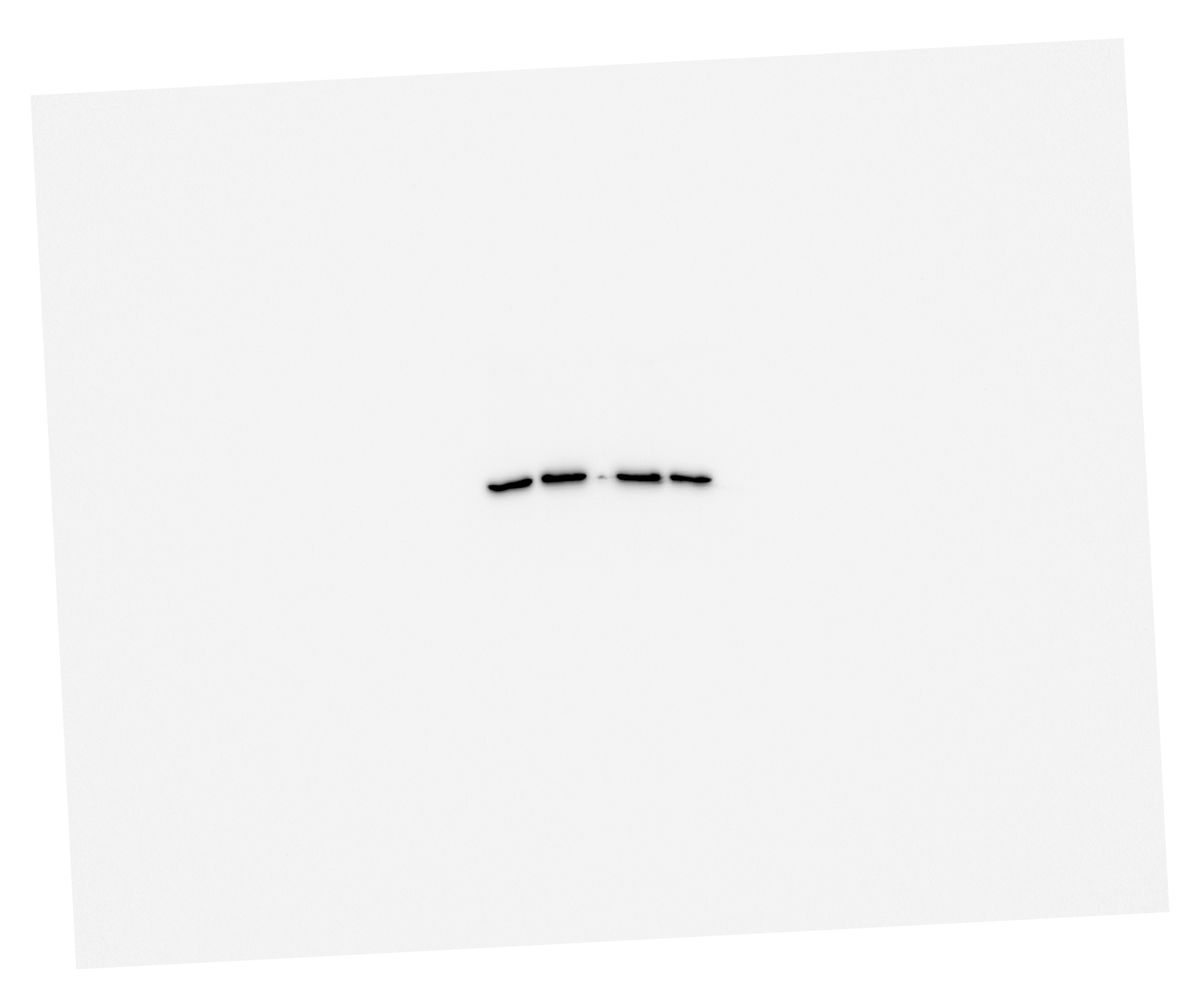

Supplement: Supplementary file 8 — Source data Fig. 2 [file 44319_2025_660_MOESM8_ESM.zip › Figure 2/F/ACTIN ATAD2 KD (Chemiluminescence).tif]

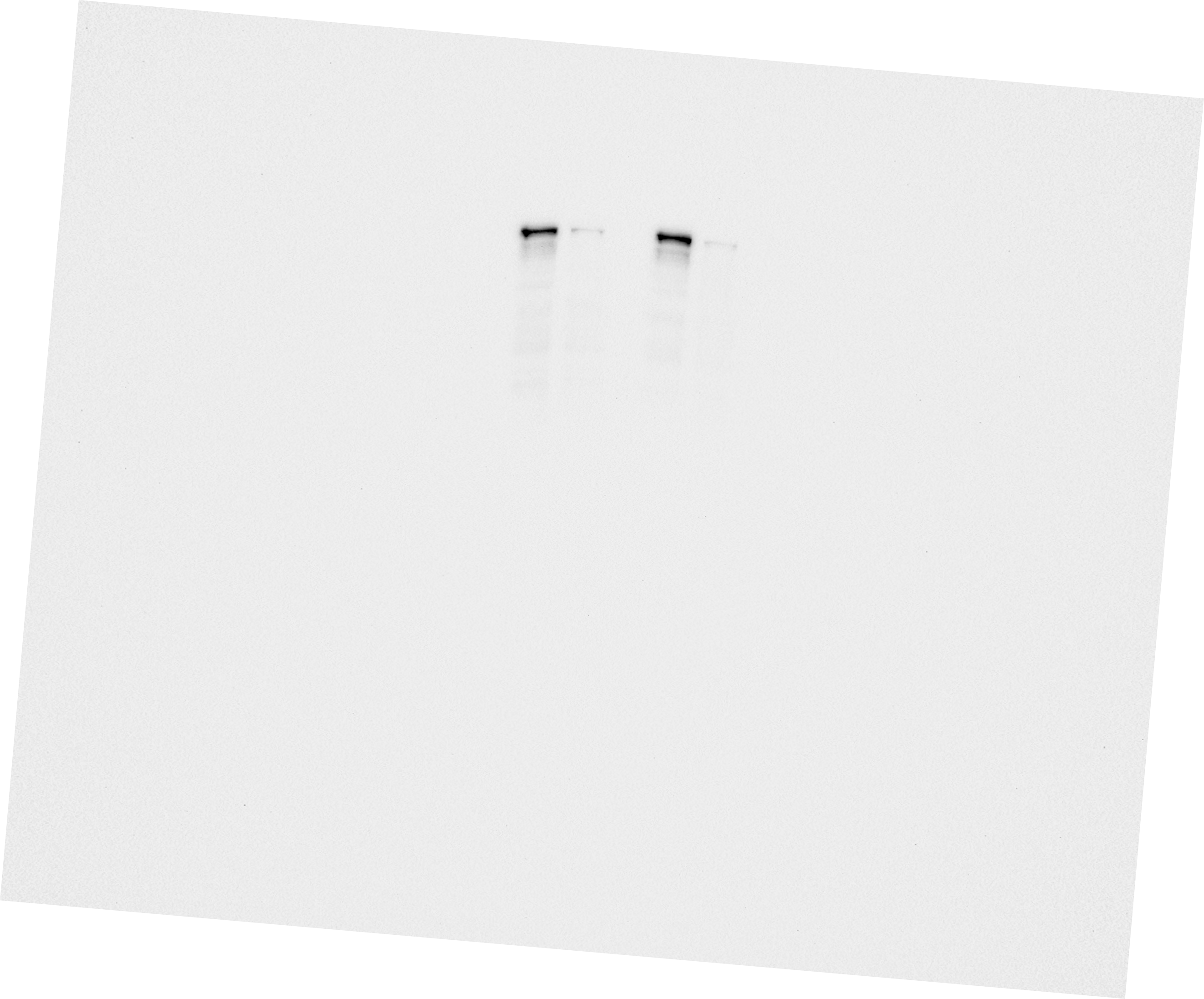

Supplement: Supplementary file 8 — Source data Fig. 2 [file 44319_2025_660_MOESM8_ESM.zip › Figure 2/F/ATAD2 KD western blotting(Chemiluminescence).tif]

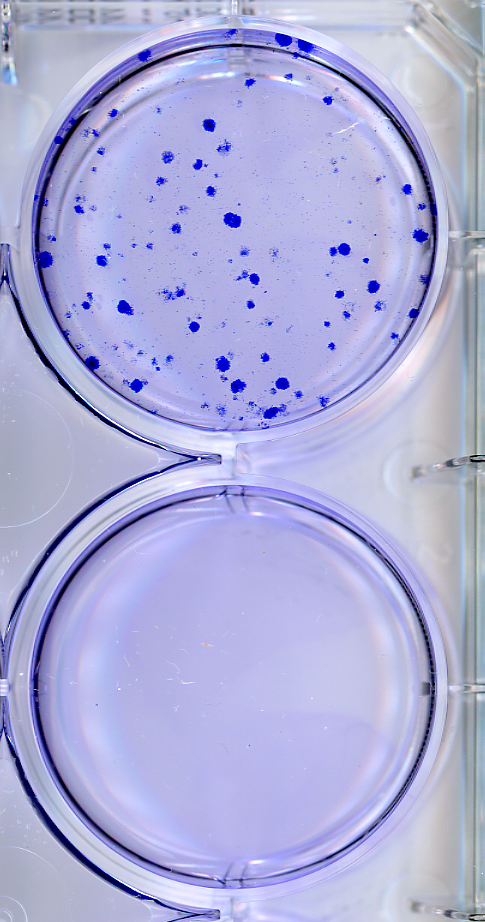

Supplement: Supplementary file 8 — Source data Fig. 2 [file 44319_2025_660_MOESM8_ESM.zip › Figure 2/B/A375_DMSO_BAY850_clonogenic.tif]

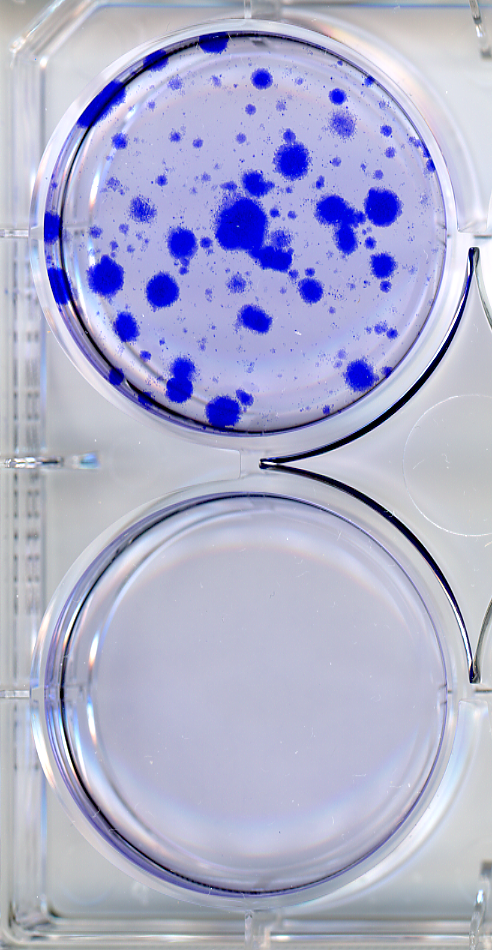

Supplement: Supplementary file 8 — Source data Fig. 2 [file 44319_2025_660_MOESM8_ESM.zip › Figure 2/B/SKMEL_2_DMSO_BAY-850_clonogenic.tif]

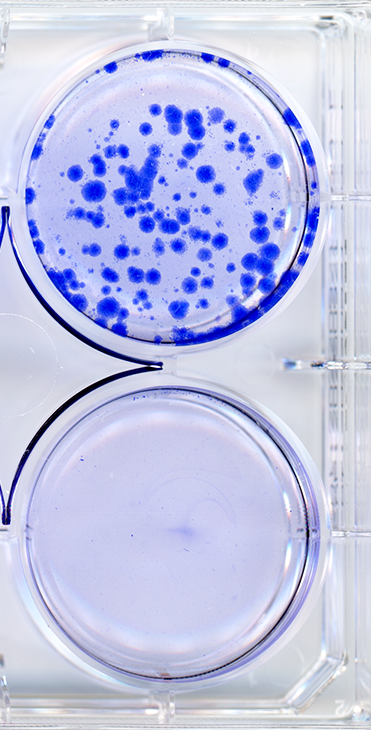

Supplement: Supplementary file 8 — Source data Fig. 2 [file 44319_2025_660_MOESM8_ESM.zip › Figure 2/B/SKMEL-103_DMSO_BAY-850_clonogenic.tif]

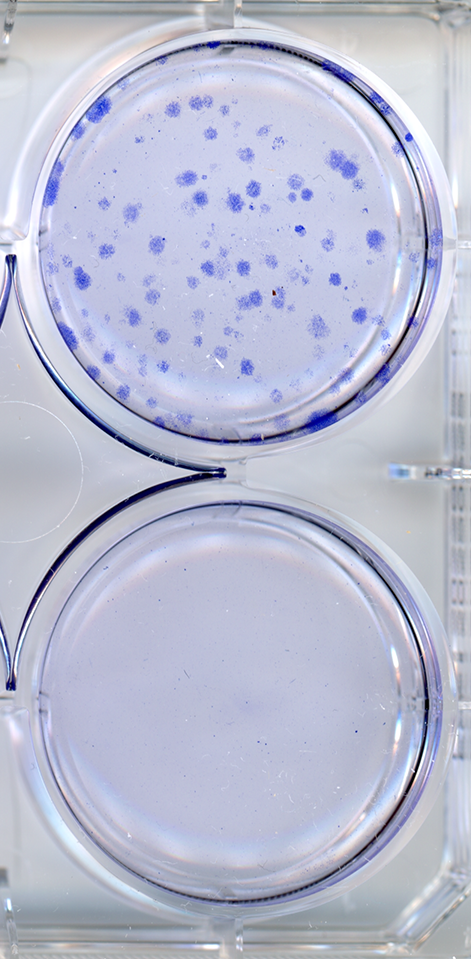

Supplement: Supplementary file 8 — Source data Fig. 2 [file 44319_2025_660_MOESM8_ESM.zip › Figure 2/B/M14_DMSO_BAY-850_clonogenic.tif]

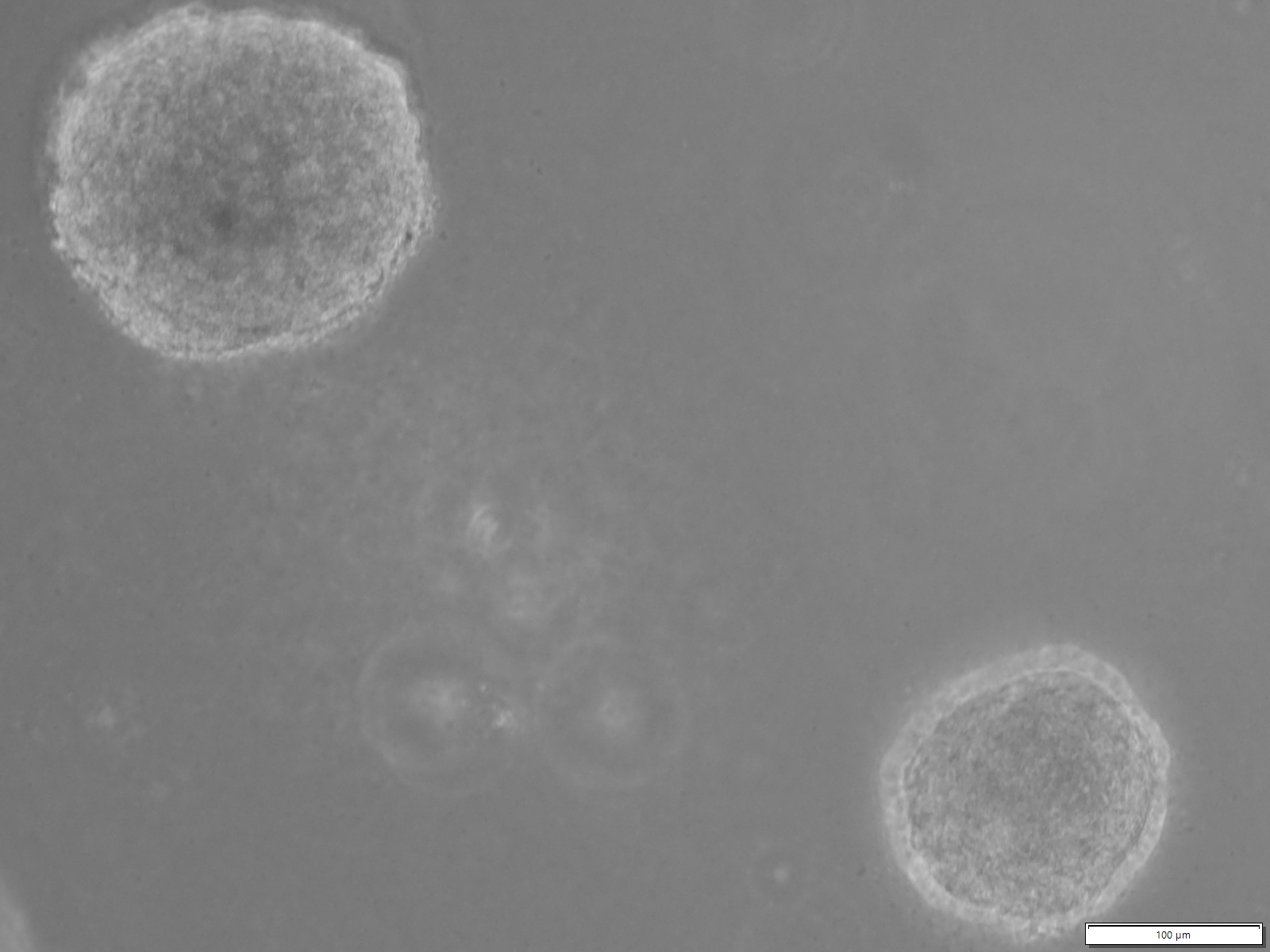

Supplement: Supplementary file 8 — Source data Fig. 2 [file 44319_2025_660_MOESM8_ESM.zip › Figure 2/C/SKMEL-2/SKMEL-2-DMSO.tif]

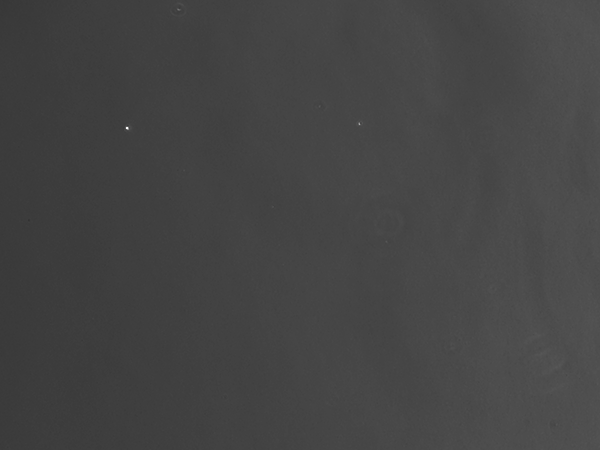

Supplement: Supplementary file 8 — Source data Fig. 2 [file 44319_2025_660_MOESM8_ESM.zip › Figure 2/C/SKMEL-2/SKMEL-2-BAY-850.tif]

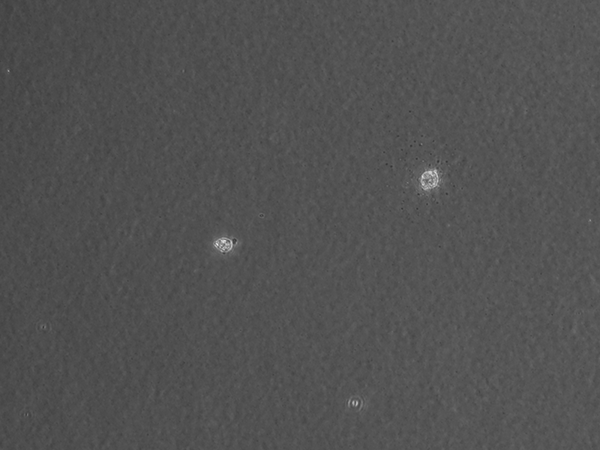

Supplement: Supplementary file 8 — Source data Fig. 2 [file 44319_2025_660_MOESM8_ESM.zip › Figure 2/C/M14/M14-BAY-850.tif]

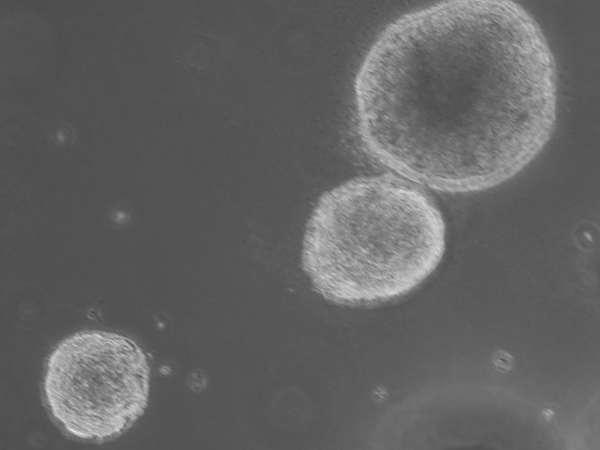

Supplement: Supplementary file 8 — Source data Fig. 2 [file 44319_2025_660_MOESM8_ESM.zip › Figure 2/C/M14/M14-DMSO.tif]

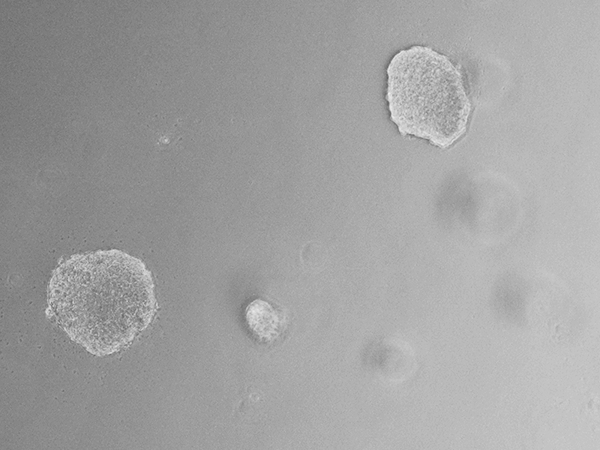

Supplement: Supplementary file 8 — Source data Fig. 2 [file 44319_2025_660_MOESM8_ESM.zip › Figure 2/C/SKMEL-103/SKMEL-103-DMSO.tif]

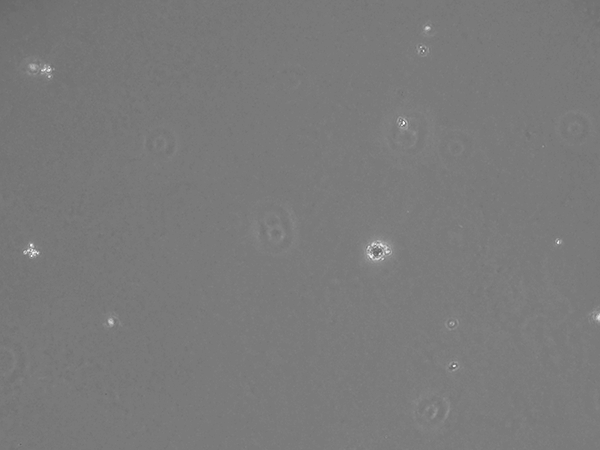

Supplement: Supplementary file 8 — Source data Fig. 2 [file 44319_2025_660_MOESM8_ESM.zip › Figure 2/C/SKMEL-103/SKMEL-103-BAY-850.tif]

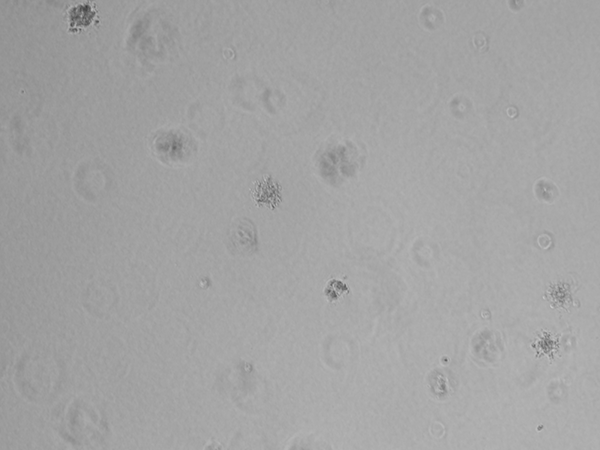

Supplement: Supplementary file 8 — Source data Fig. 2 [file 44319_2025_660_MOESM8_ESM.zip › Figure 2/C/A375/A375-BAY-850.tif]

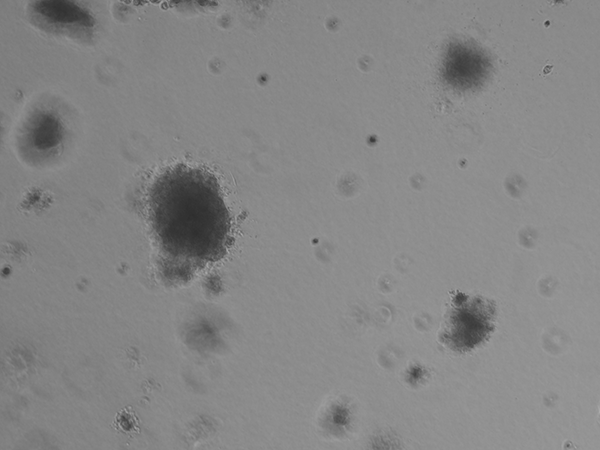

Supplement: Supplementary file 8 — Source data Fig. 2 [file 44319_2025_660_MOESM8_ESM.zip › Figure 2/C/A375/A375-DMSO.tif]

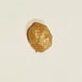

Supplement: Supplementary file 9 — Source data Fig. 3 [file 44319_2025_660_MOESM9_ESM.zip › Figure 3/3B/BAY-850 trt.jpg]

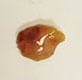

Supplement: Supplementary file 9 — Source data Fig. 3 [file 44319_2025_660_MOESM9_ESM.zip › Figure 3/3B/Vehicle.jpg]

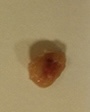

Supplement: Supplementary file 9 — Source data Fig. 3 [file 44319_2025_660_MOESM9_ESM.zip › Figure 3/3C/BAy-850 trt.jpg]

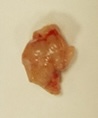

Supplement: Supplementary file 9 — Source data Fig. 3 [file 44319_2025_660_MOESM9_ESM.zip › Figure 3/3C/Vehicle.jpg]

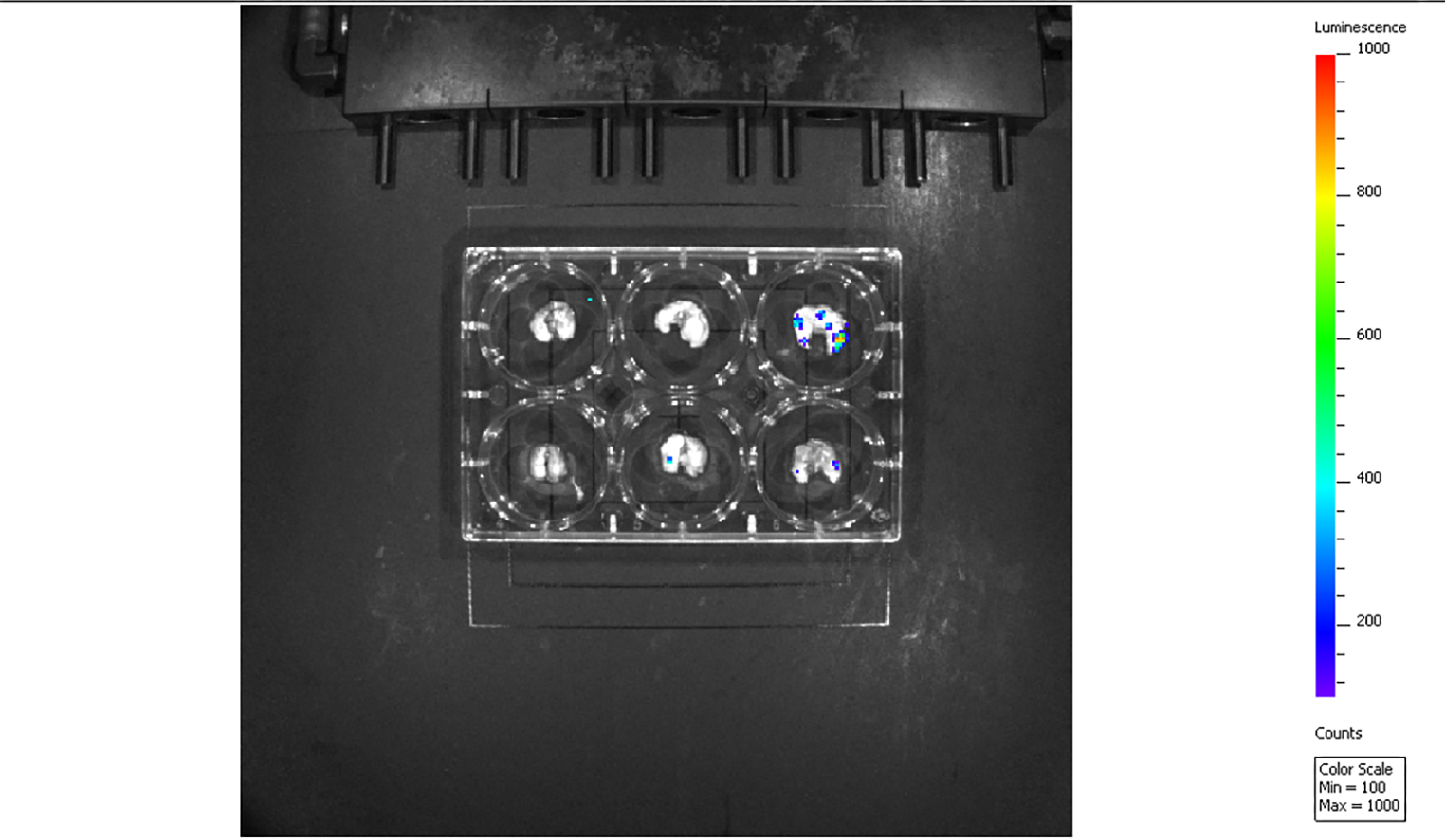

Supplement: Supplementary file 9 — Source data Fig. 3 [file 44319_2025_660_MOESM9_ESM.zip › Figure 3/3F/Lungs_BAY-850_A375.tif]

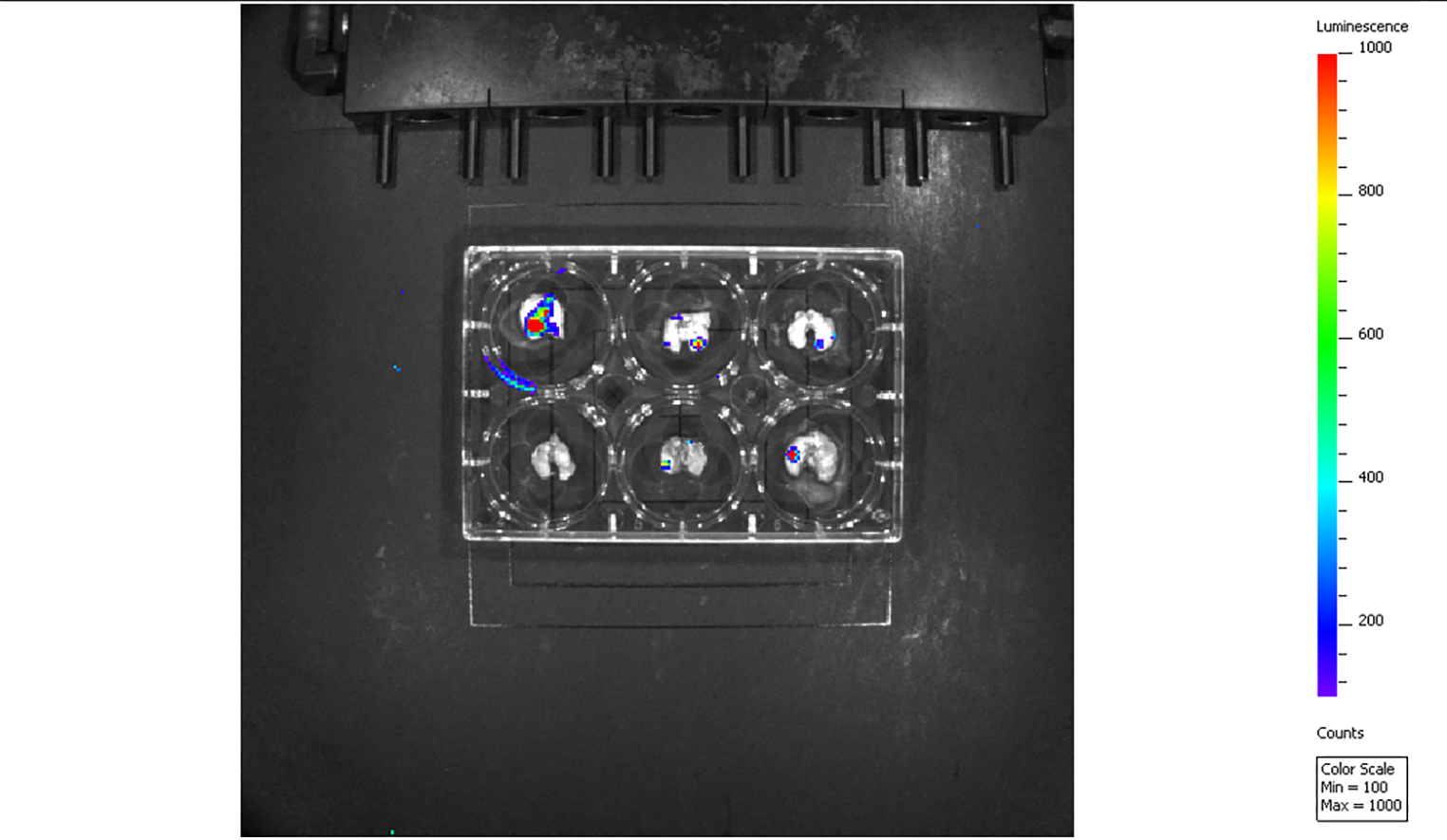

Supplement: Supplementary file 9 — Source data Fig. 3 [file 44319_2025_660_MOESM9_ESM.zip › Figure 3/3F/Lungs_Vehicle_A375.tif]

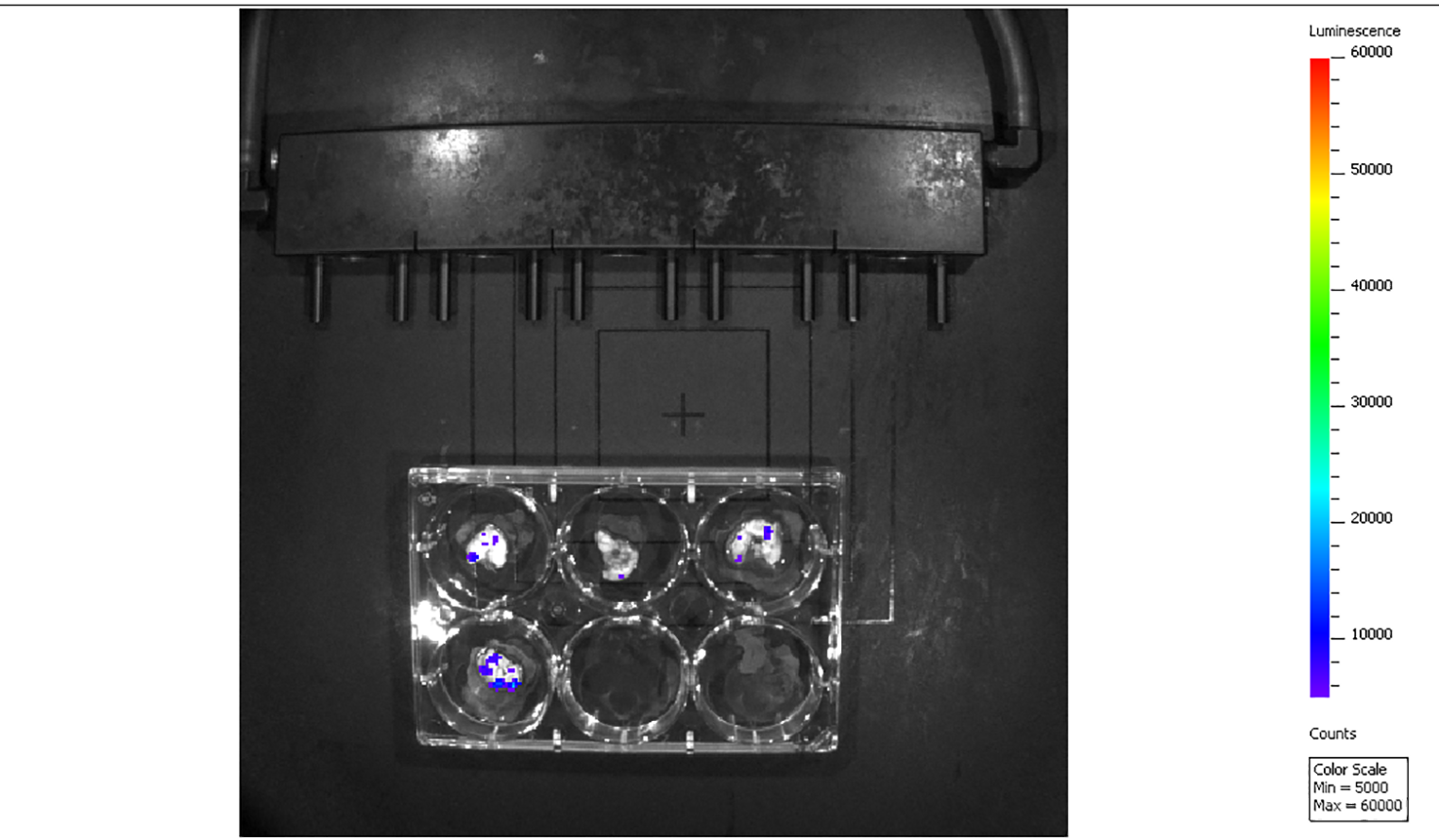

Supplement: Supplementary file 9 — Source data Fig. 3 [file 44319_2025_660_MOESM9_ESM.zip › Figure 3/3I/SKMEL-103_lungs_BAY-850.tif]

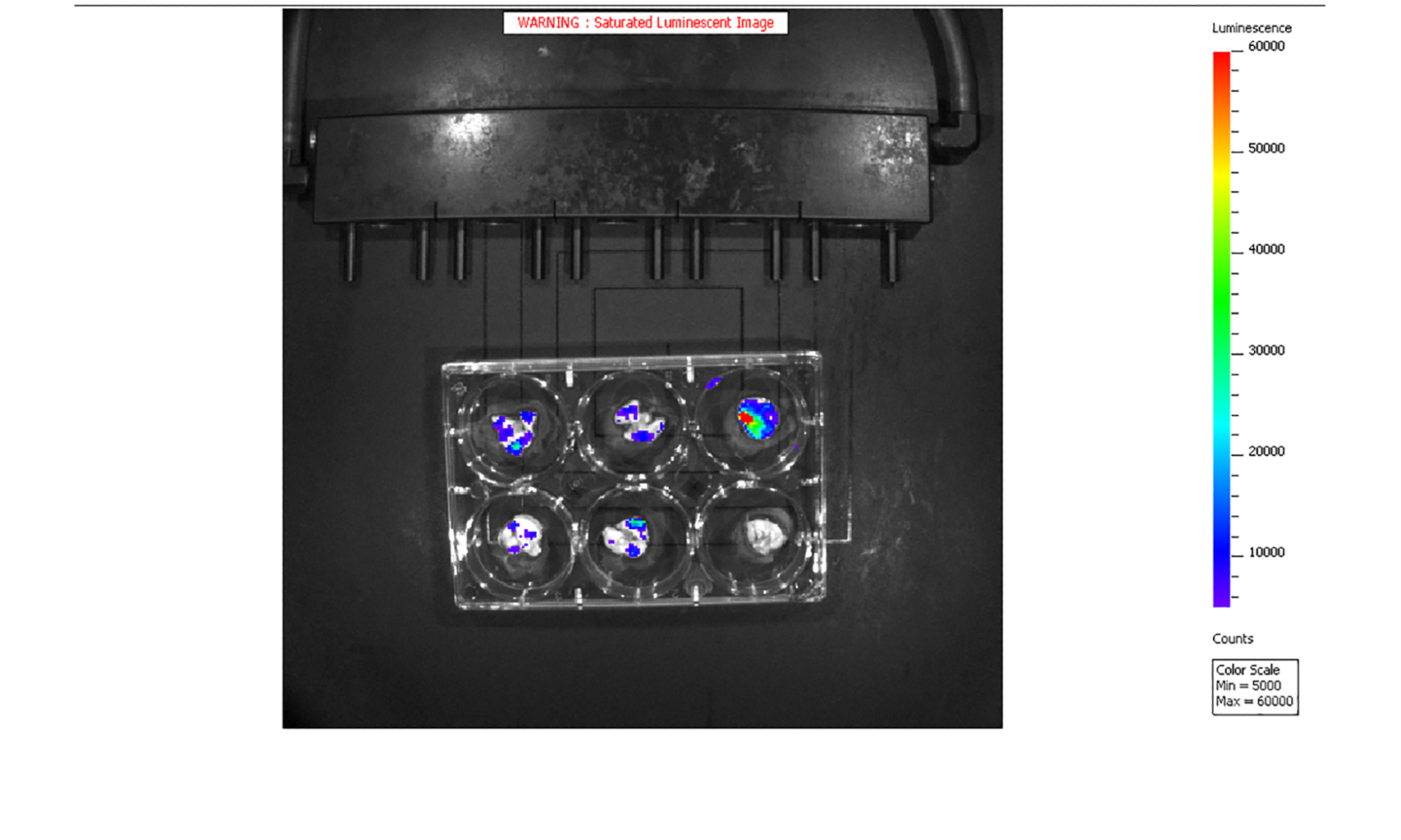

Supplement: Supplementary file 9 — Source data Fig. 3 [file 44319_2025_660_MOESM9_ESM.zip › Figure 3/3I/SKMEL-103_lungs_Vehicle.tif]

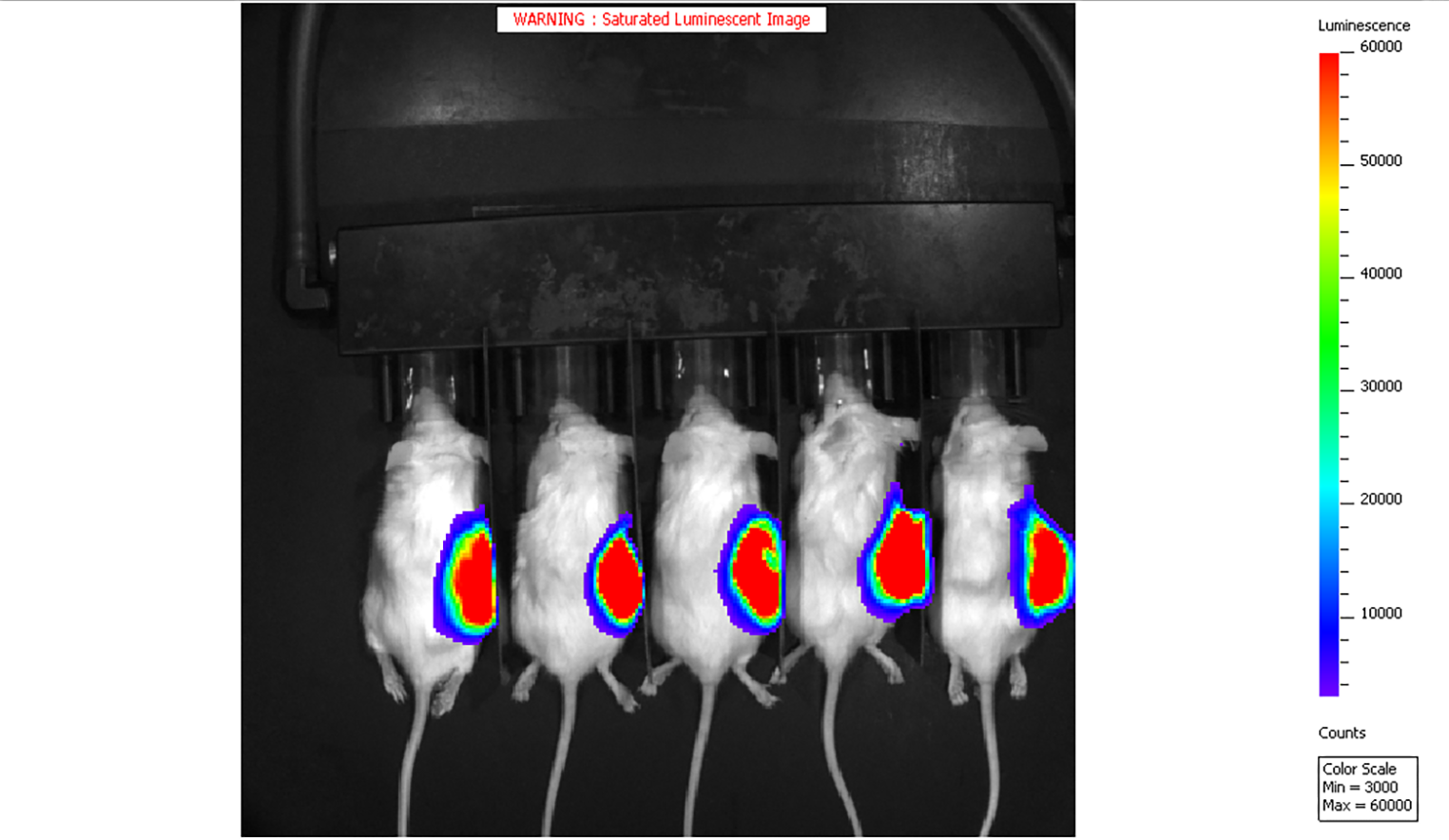

Supplement: Supplementary file 9 — Source data Fig. 3 [file 44319_2025_660_MOESM9_ESM.zip › Figure 3/3D/Vehicle/A375_Vehicle_Week 6.tif]

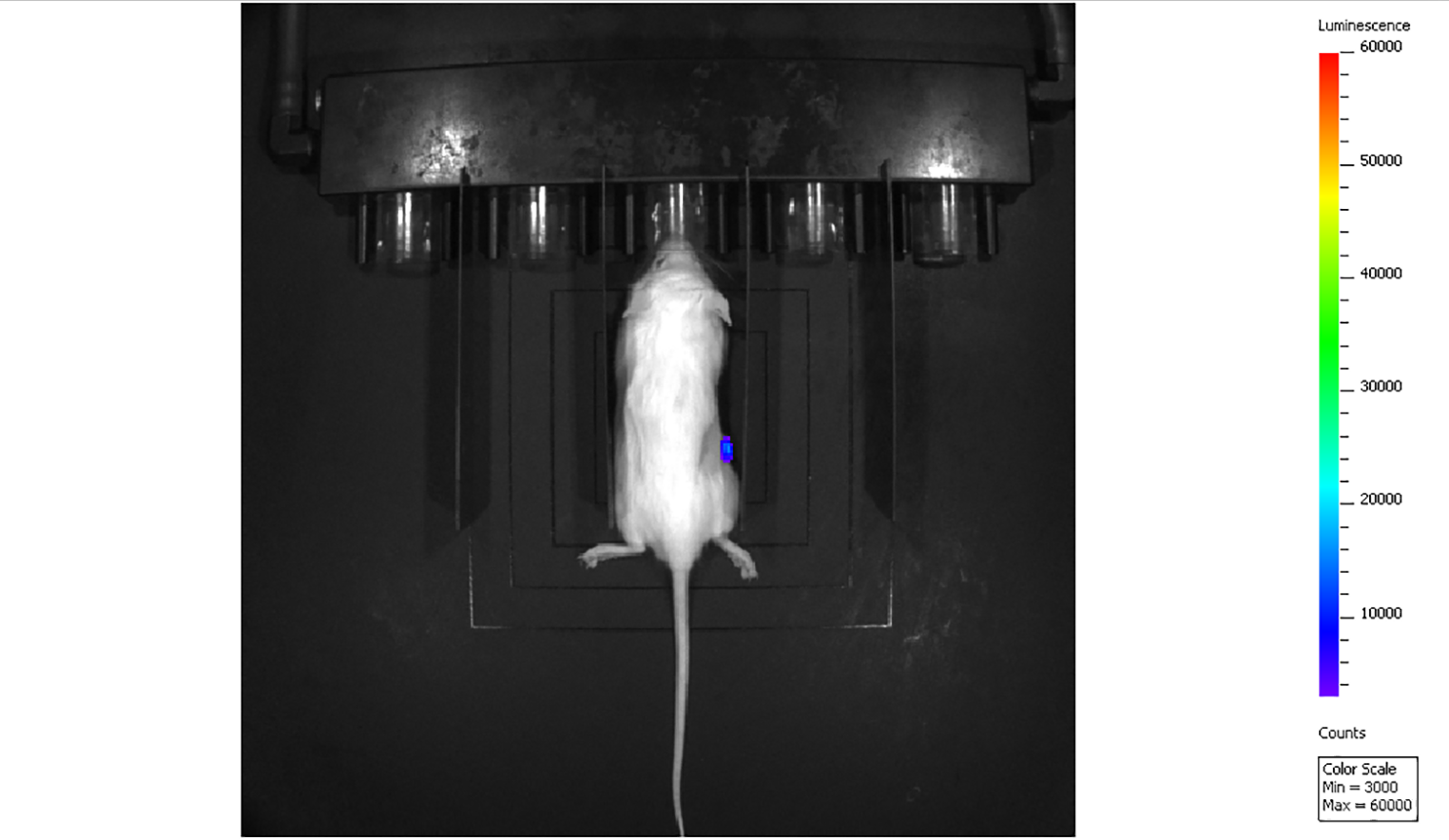

Supplement: Supplementary file 9 — Source data Fig. 3 [file 44319_2025_660_MOESM9_ESM.zip › Figure 3/3D/Vehicle/A375_Vehicle_Week 2_6th animal.tif]

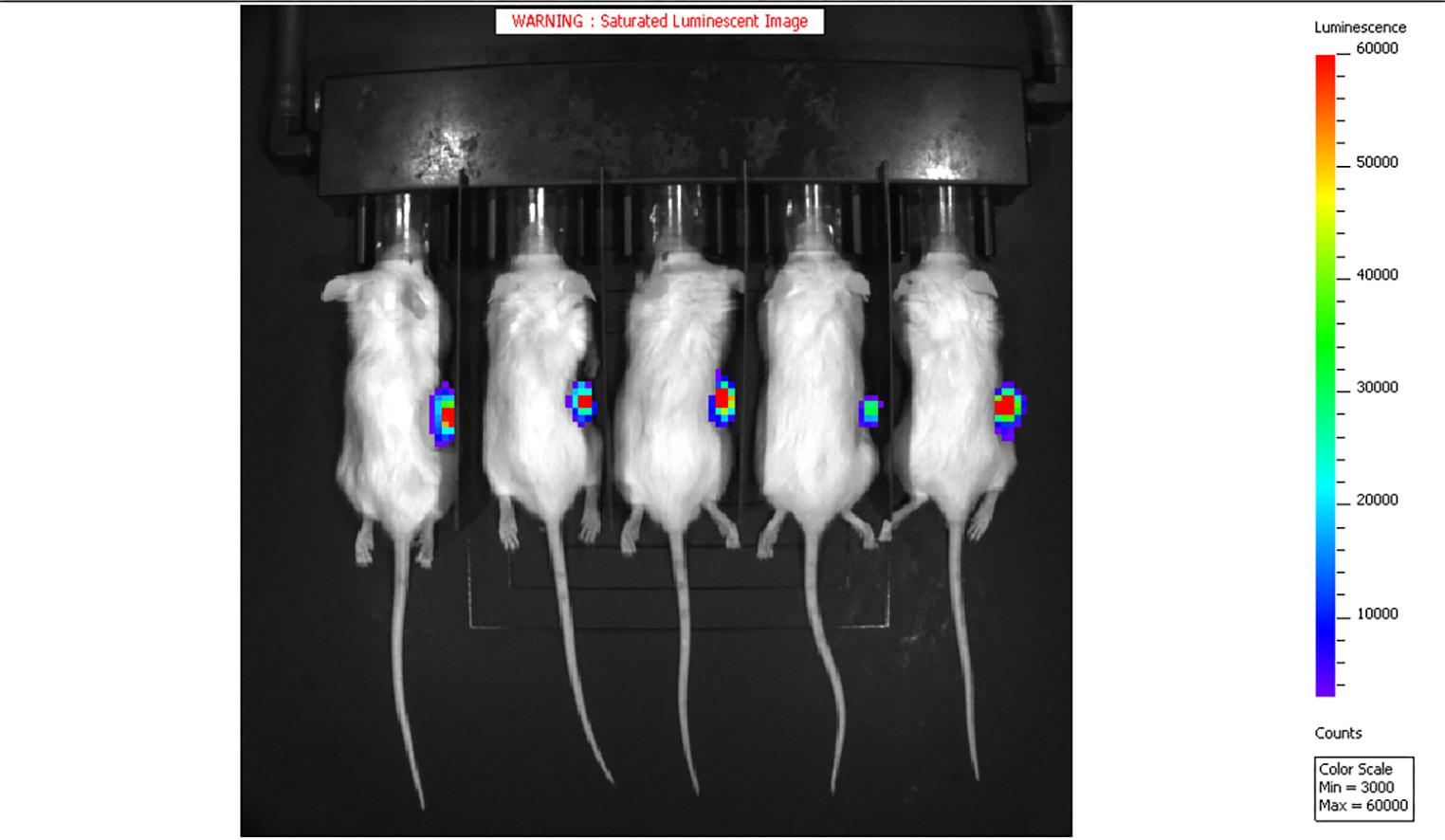

Supplement: Supplementary file 9 — Source data Fig. 3 [file 44319_2025_660_MOESM9_ESM.zip › Figure 3/3D/Vehicle/A375_Vehicle_Week 2.tif]

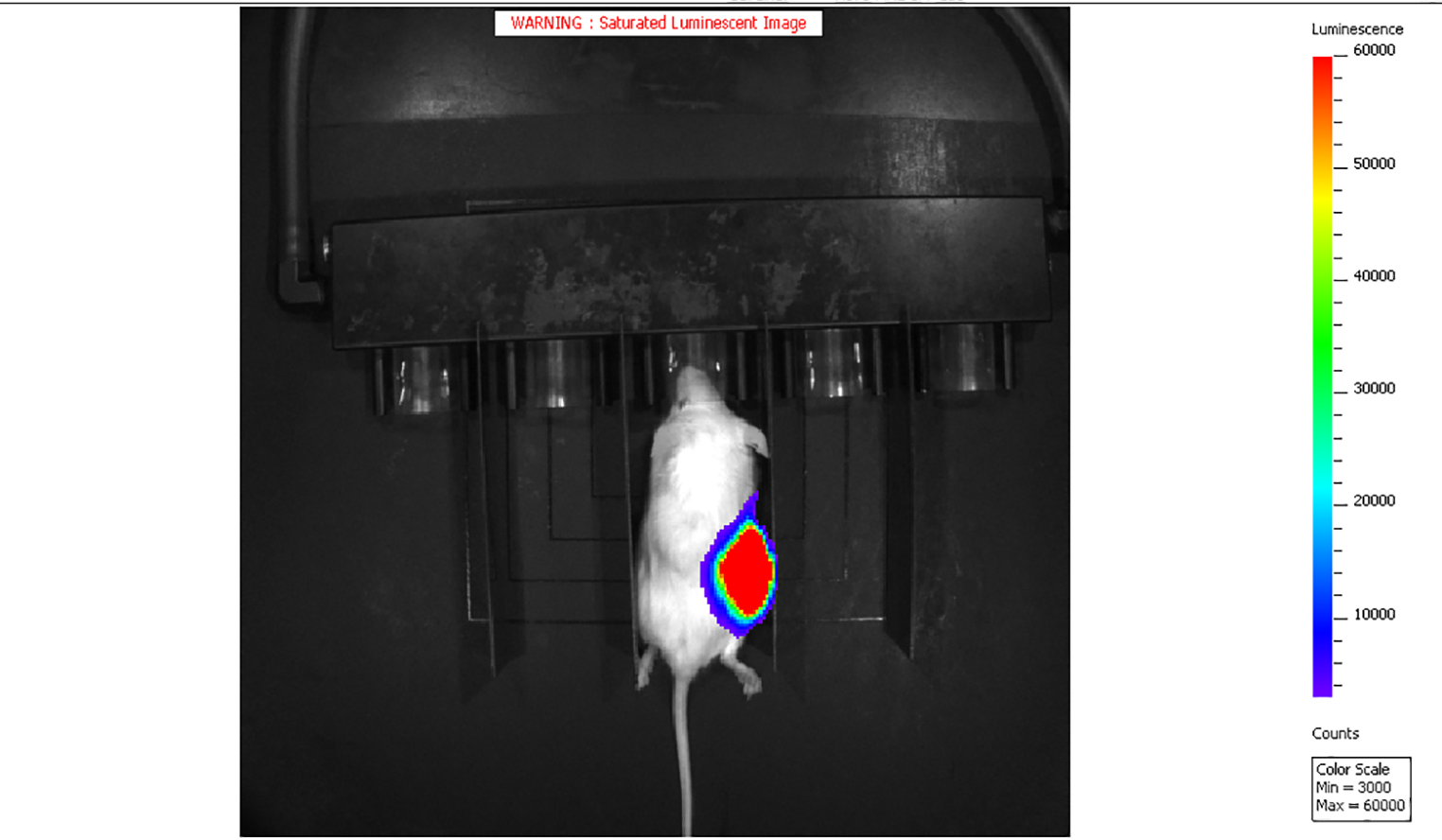

Supplement: Supplementary file 9 — Source data Fig. 3 [file 44319_2025_660_MOESM9_ESM.zip › Figure 3/3D/Vehicle/A375_Vehicle_Week 6-6th animal.tif]

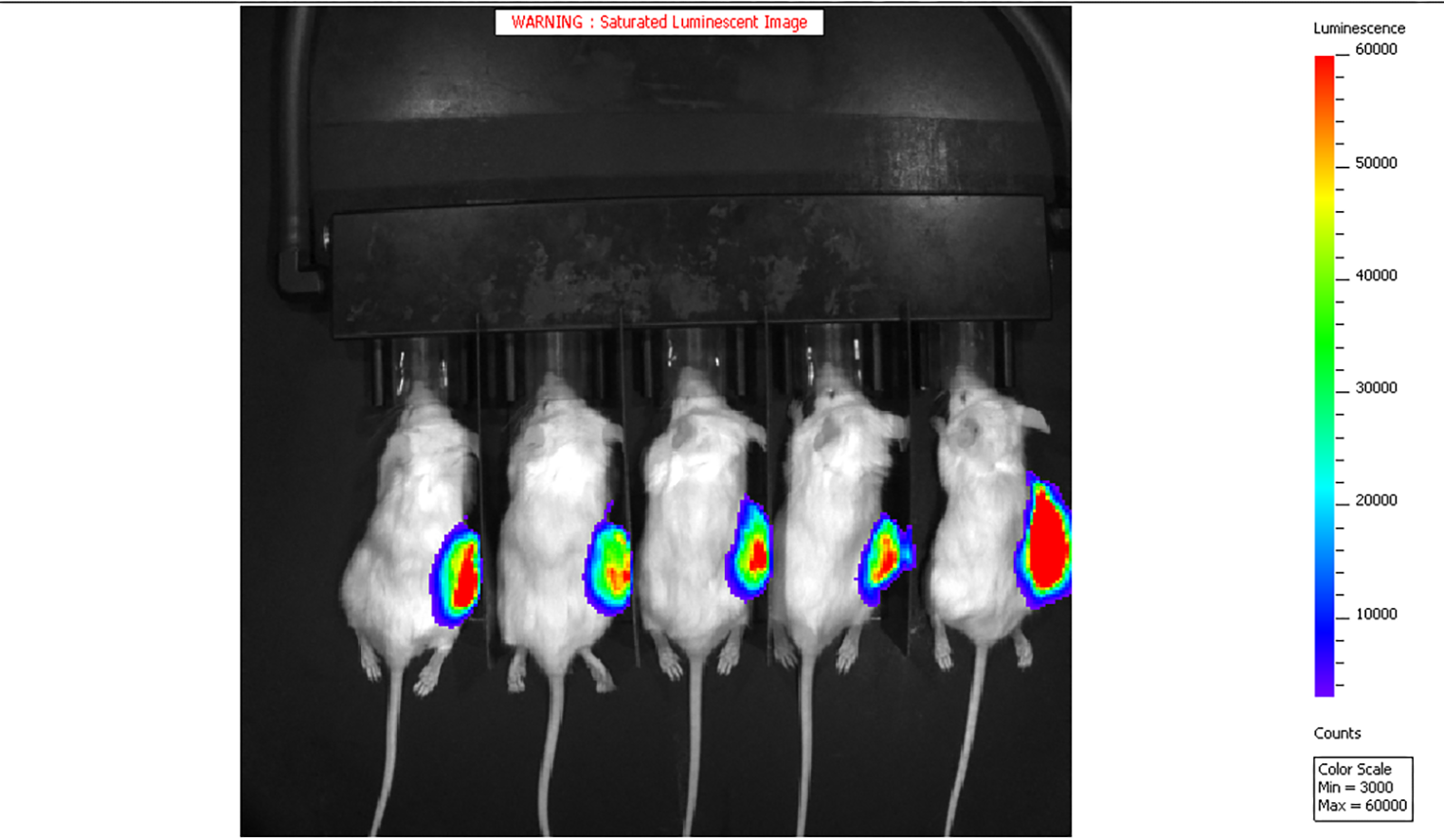

Supplement: Supplementary file 9 — Source data Fig. 3 [file 44319_2025_660_MOESM9_ESM.zip › Figure 3/3D/BAY-850/A375_BAY-850_Week 6.tif]

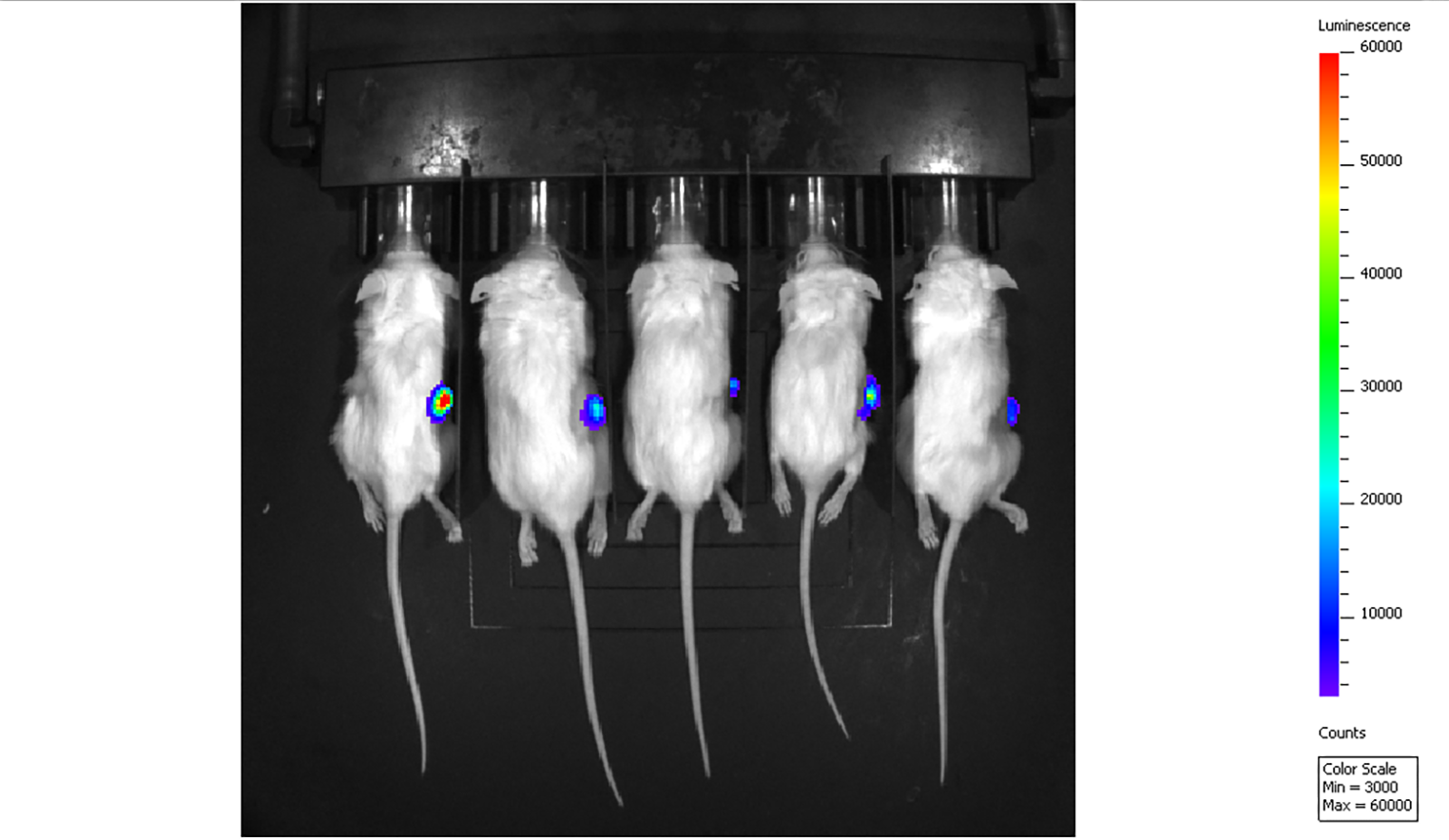

Supplement: Supplementary file 9 — Source data Fig. 3 [file 44319_2025_660_MOESM9_ESM.zip › Figure 3/3D/BAY-850/A375_BAY-850_Week 2.tif]

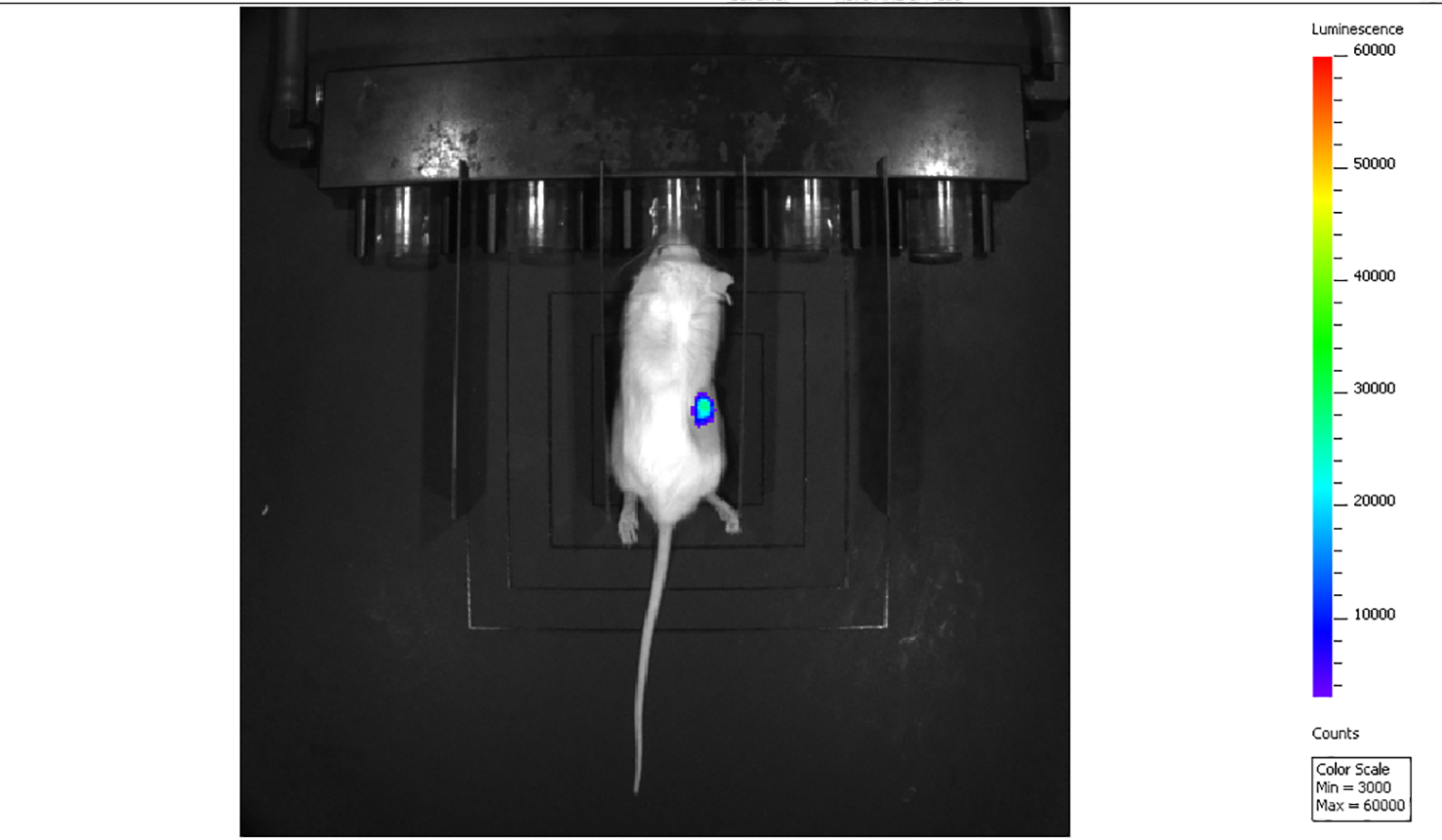

Supplement: Supplementary file 9 — Source data Fig. 3 [file 44319_2025_660_MOESM9_ESM.zip › Figure 3/3D/BAY-850/A375_BAY-850_Week 2-6th animal.tif]

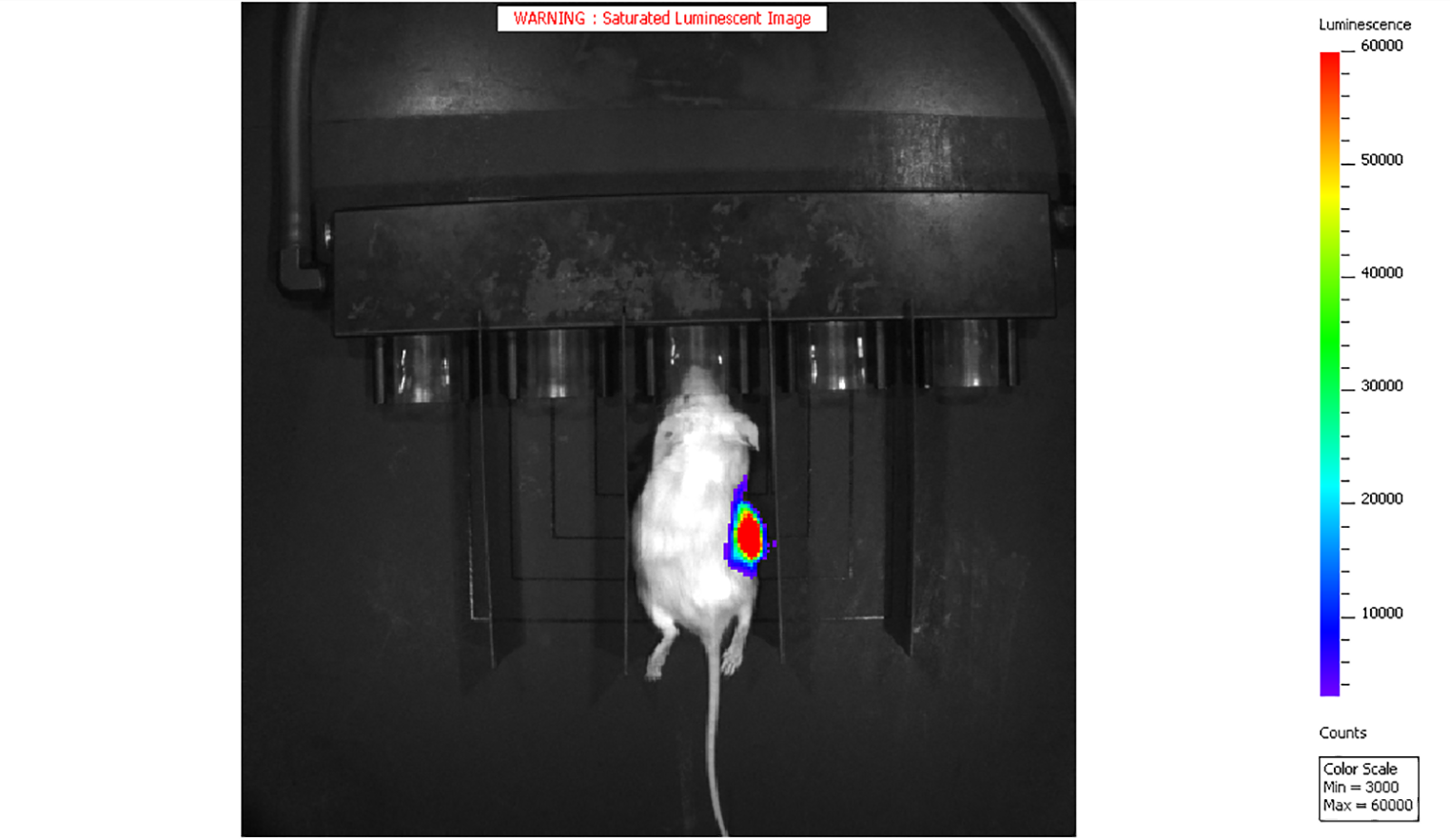

Supplement: Supplementary file 9 — Source data Fig. 3 [file 44319_2025_660_MOESM9_ESM.zip › Figure 3/3D/BAY-850/A375_BAY-850_Week 6-6th animal.tif]

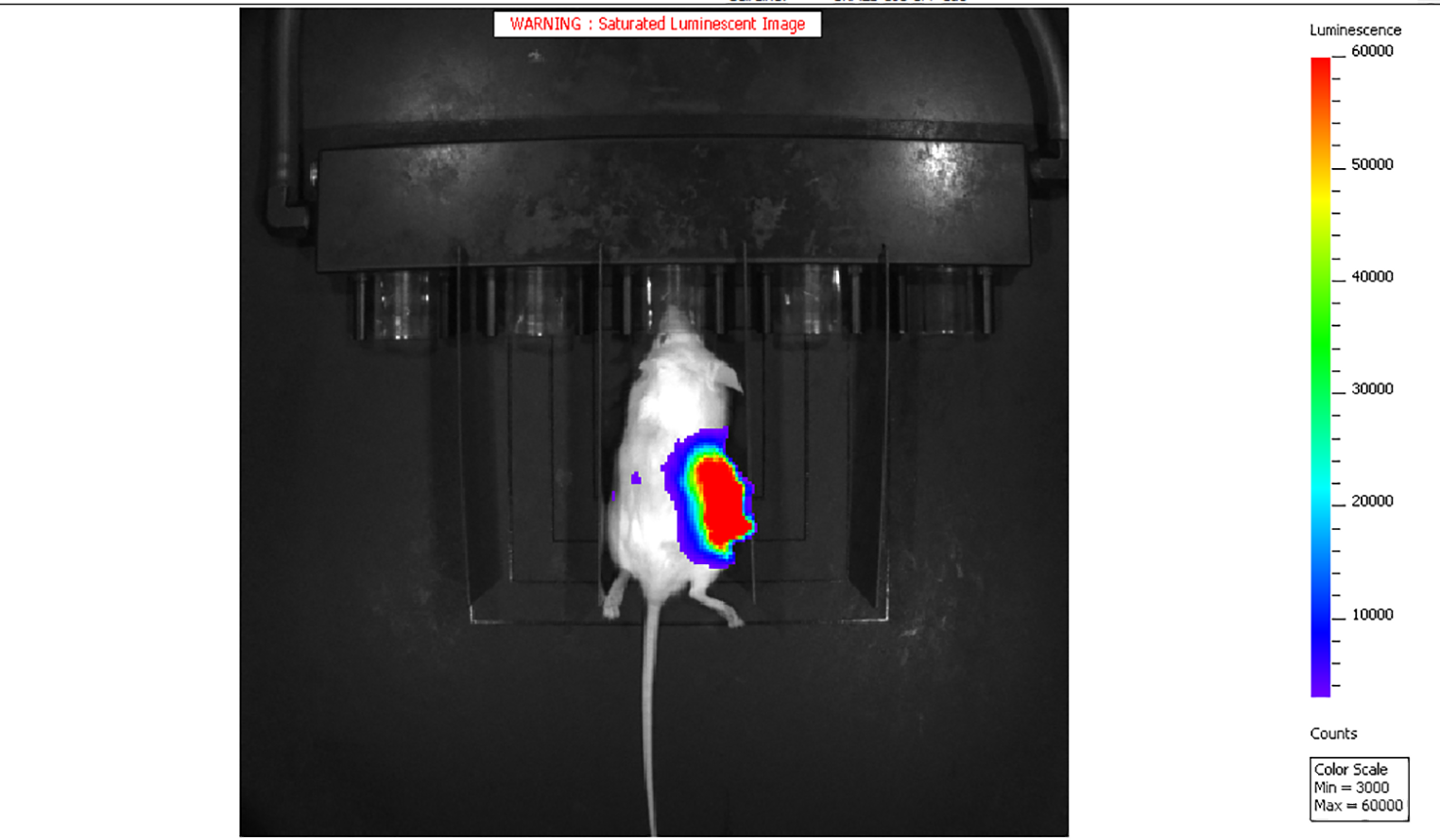

Supplement: Supplementary file 9 — Source data Fig. 3 [file 44319_2025_660_MOESM9_ESM.zip › Figure 3/3G/Vehicle/SKMEL-103_Vehicle_Week 6-6th animal.tif]

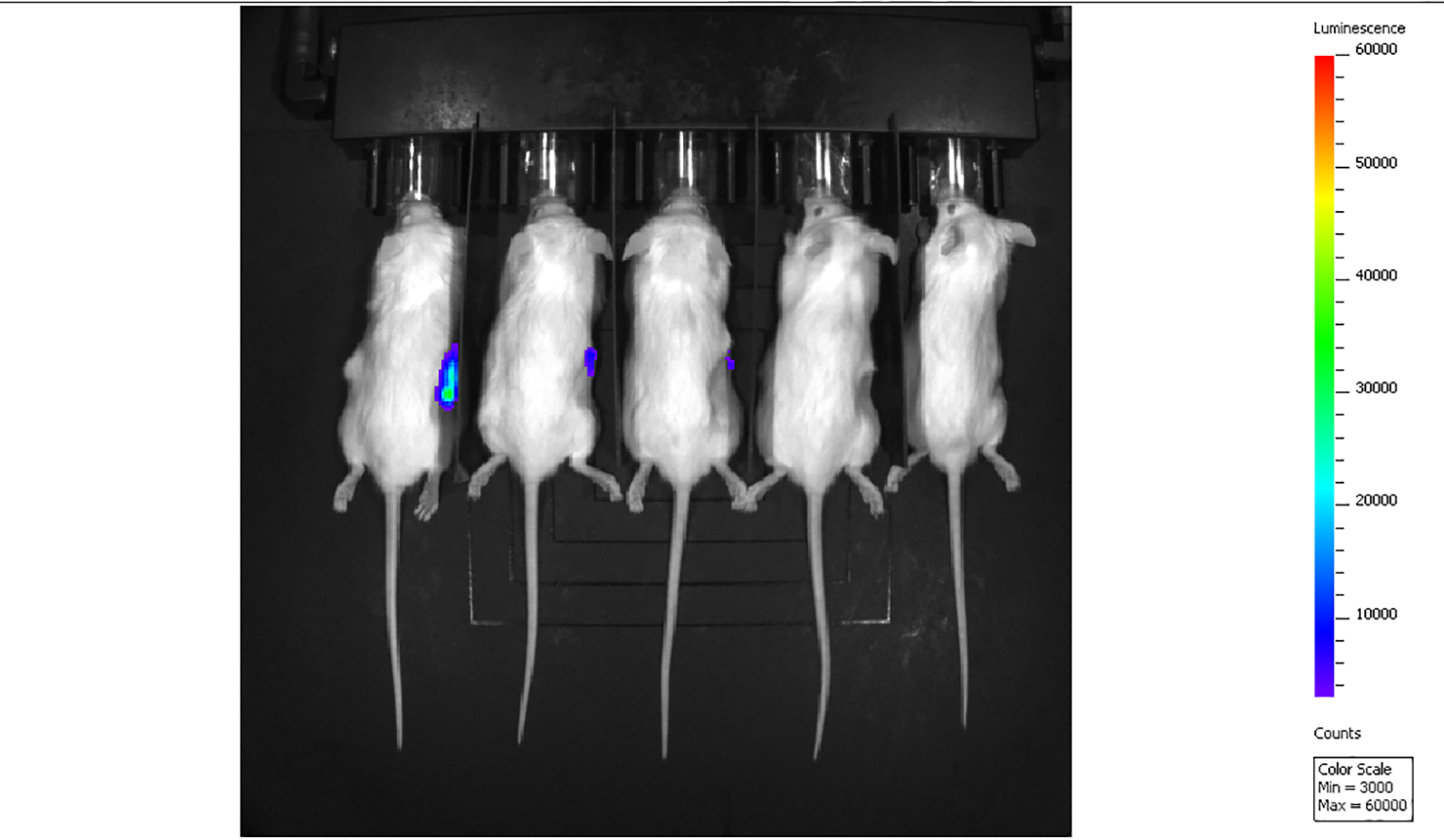

Supplement: Supplementary file 9 — Source data Fig. 3 [file 44319_2025_660_MOESM9_ESM.zip › Figure 3/3G/Vehicle/SKMEL-103_Vehicle_Week 2.tif]

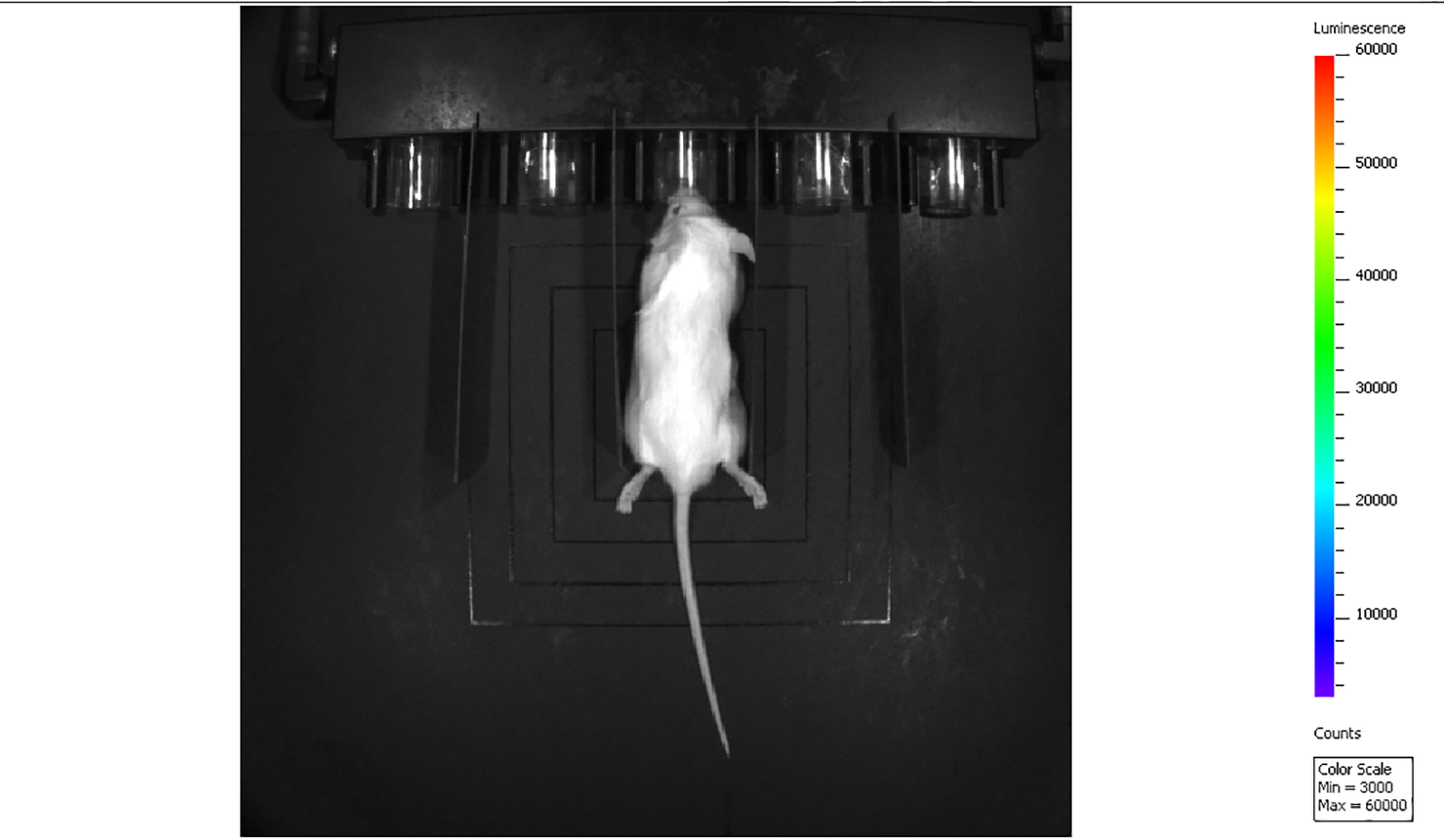

Supplement: Supplementary file 9 — Source data Fig. 3 [file 44319_2025_660_MOESM9_ESM.zip › Figure 3/3G/Vehicle/SKMEL-103_Vehicle_Week 2-6th animal.tif]

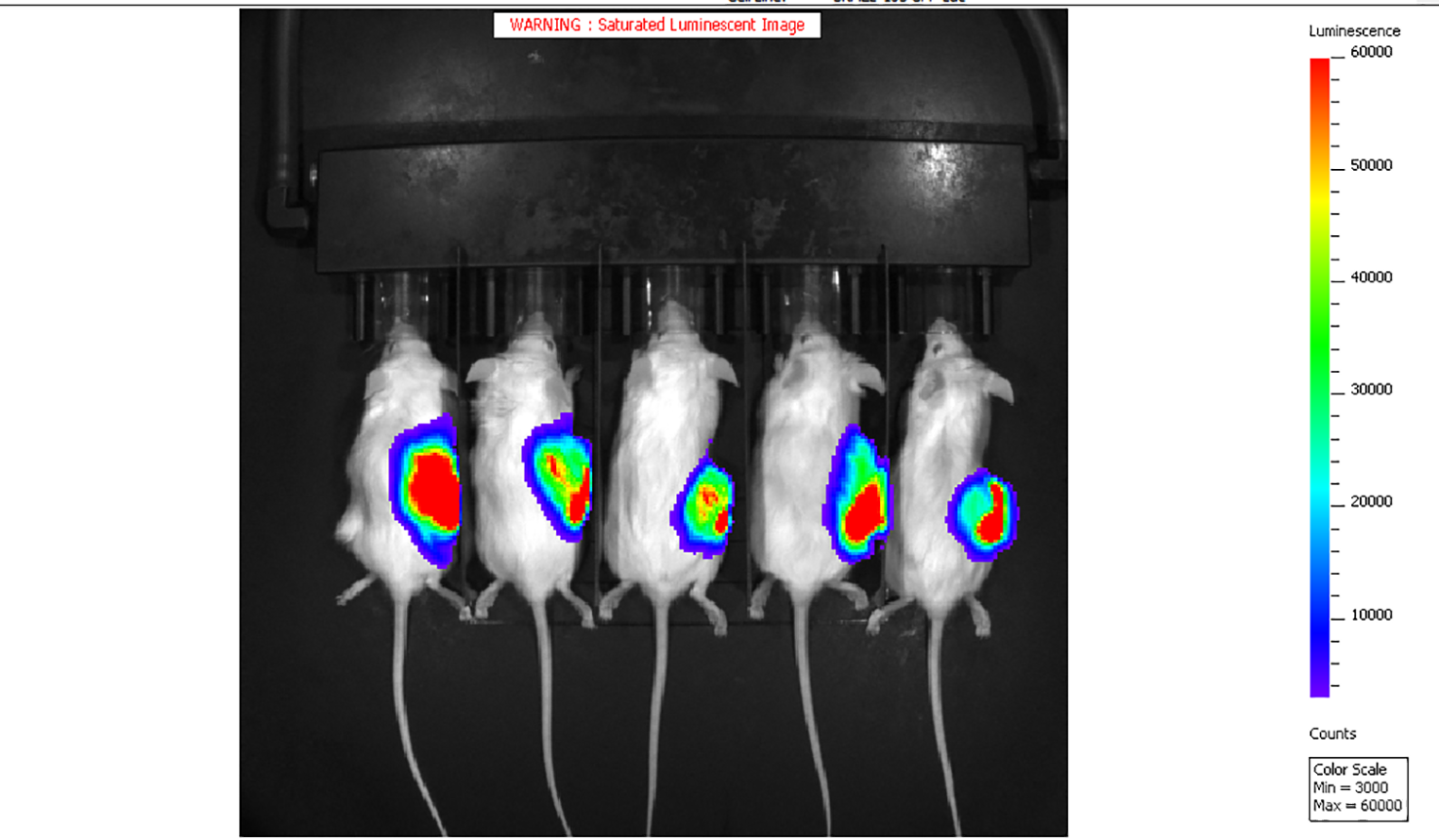

Supplement: Supplementary file 9 — Source data Fig. 3 [file 44319_2025_660_MOESM9_ESM.zip › Figure 3/3G/Vehicle/SKMEL-103_Vehicle_Week 6.tif]

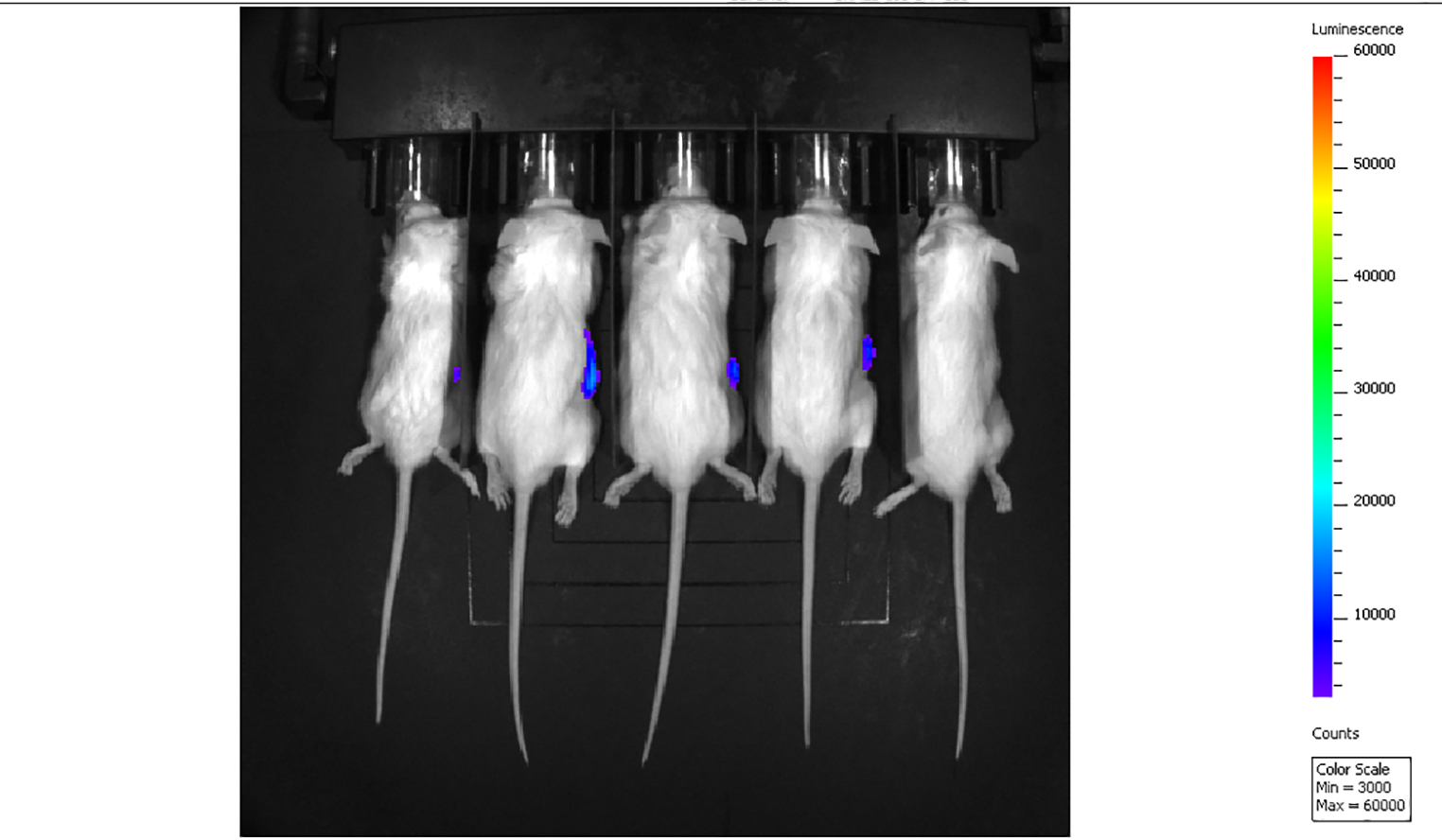

Supplement: Supplementary file 9 — Source data Fig. 3 [file 44319_2025_660_MOESM9_ESM.zip › Figure 3/3G/BAY-850/SKMEL-103_BAY-850_Week 2.tif]

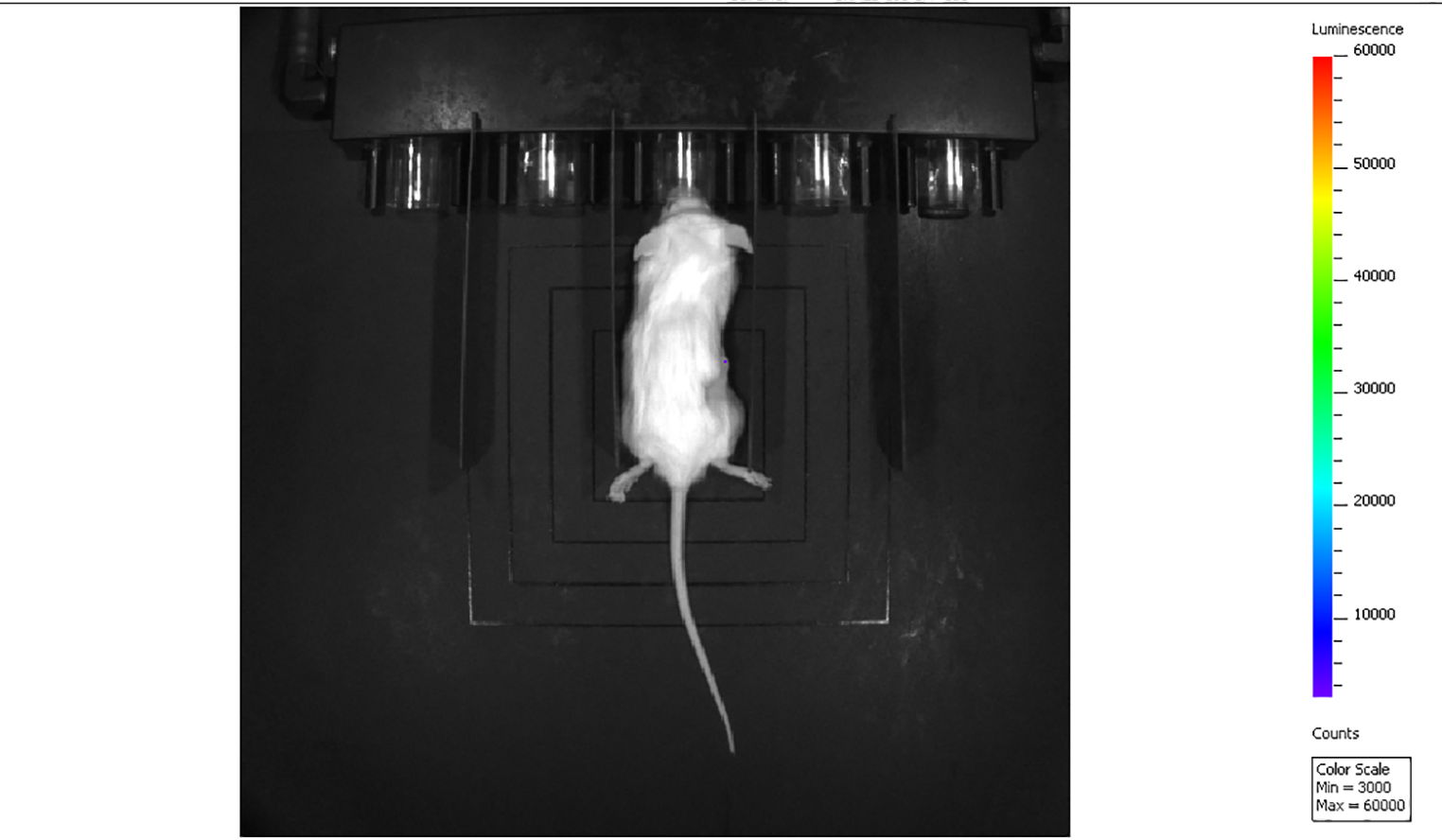

Supplement: Supplementary file 9 — Source data Fig. 3 [file 44319_2025_660_MOESM9_ESM.zip › Figure 3/3G/BAY-850/SKMEL-103_BAY-850_Week 2-6th animal.tif]

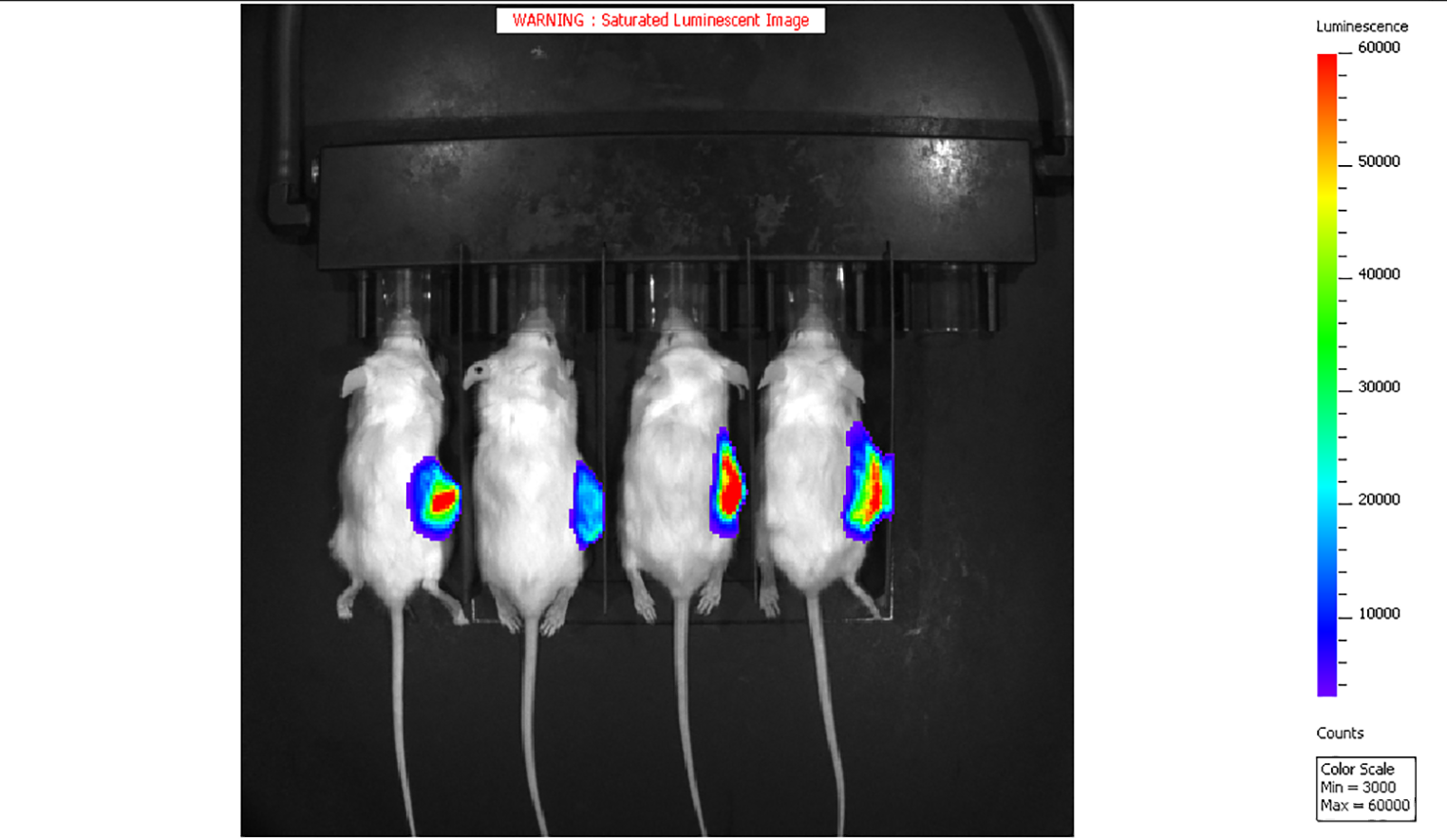

Supplement: Supplementary file 9 — Source data Fig. 3 [file 44319_2025_660_MOESM9_ESM.zip › Figure 3/3G/BAY-850/SKMEL-103_BAY-850_Week 5.tif]

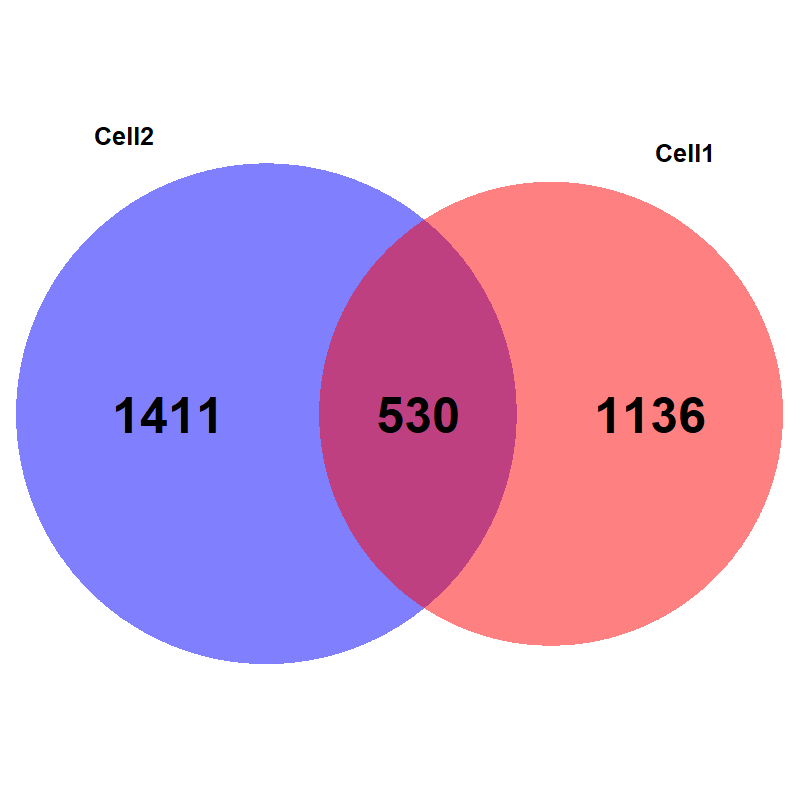

Supplement: Supplementary file 10 — Source data Fig. 4 [file 44319_2025_660_MOESM10_ESM.zip › Figure 5/5A/A375_SKMEL-103_down_venn_diagram copy.tiff]

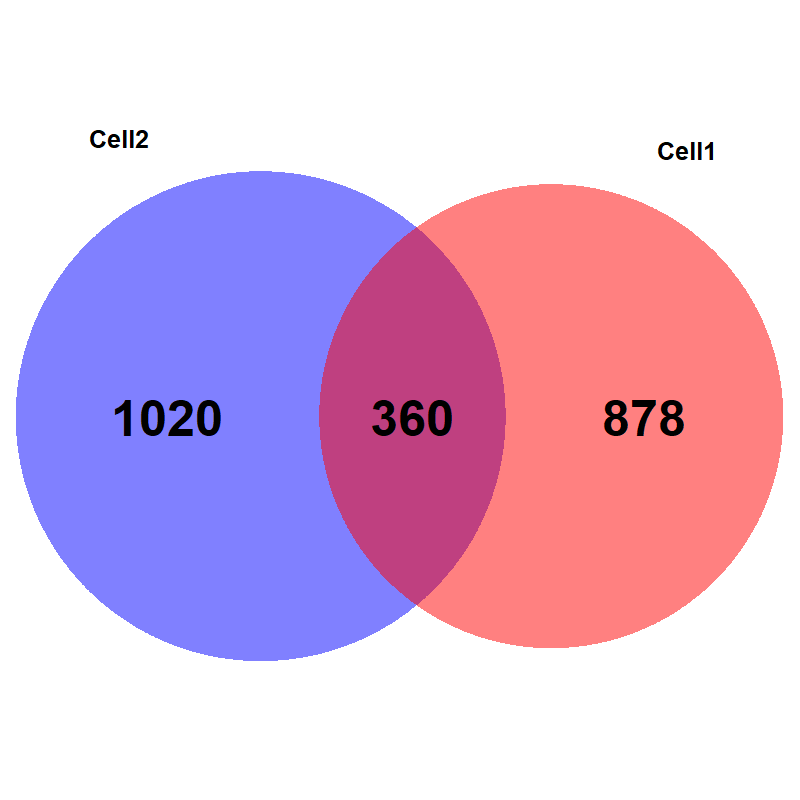

Supplement: Supplementary file 10 — Source data Fig. 4 [file 44319_2025_660_MOESM10_ESM.zip › Figure 5/5A/A375_SKMEL-103_up_venn_diagram copy.tiff]

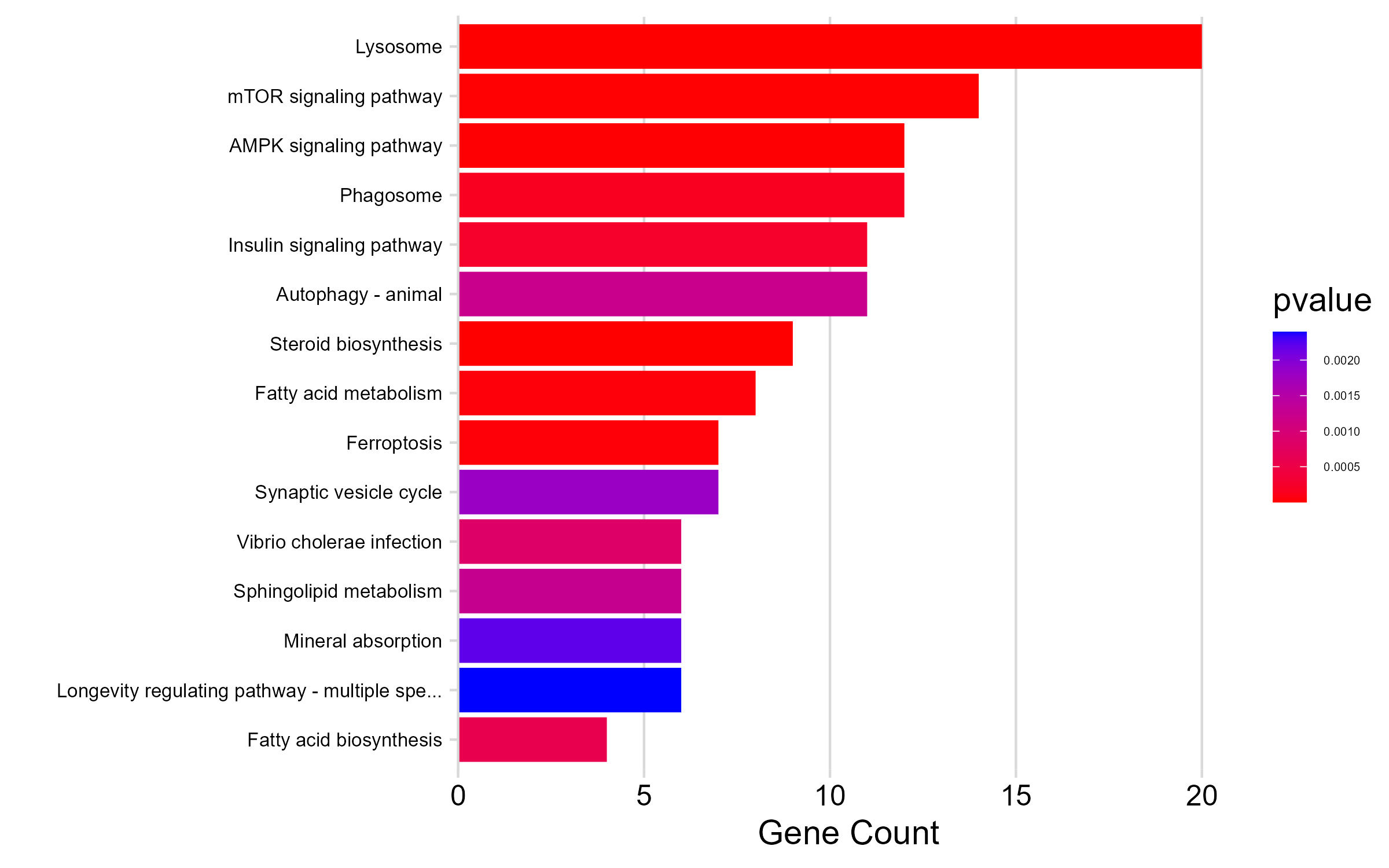

Supplement: Supplementary file 10 — Source data Fig. 4 [file 44319_2025_660_MOESM10_ESM.zip › Figure 5/5C/A375_SKMEL-103_common_UP_KEGG_enrichment copy.tiff]

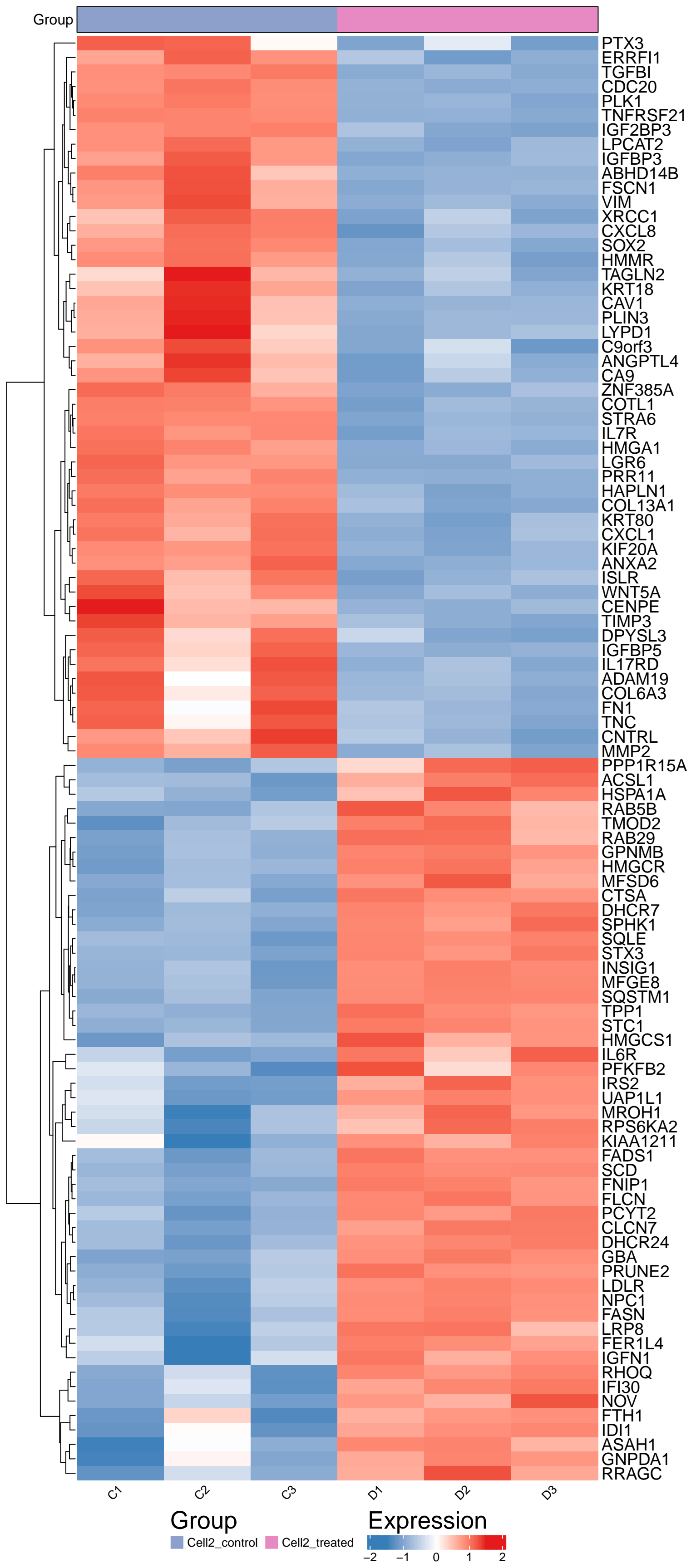

Supplement: Supplementary file 10 — Source data Fig. 4 [file 44319_2025_660_MOESM10_ESM.zip › Figure 5/5B/A375_SKMEL-103_common_top100_genesCell2_heatmap.pdf]

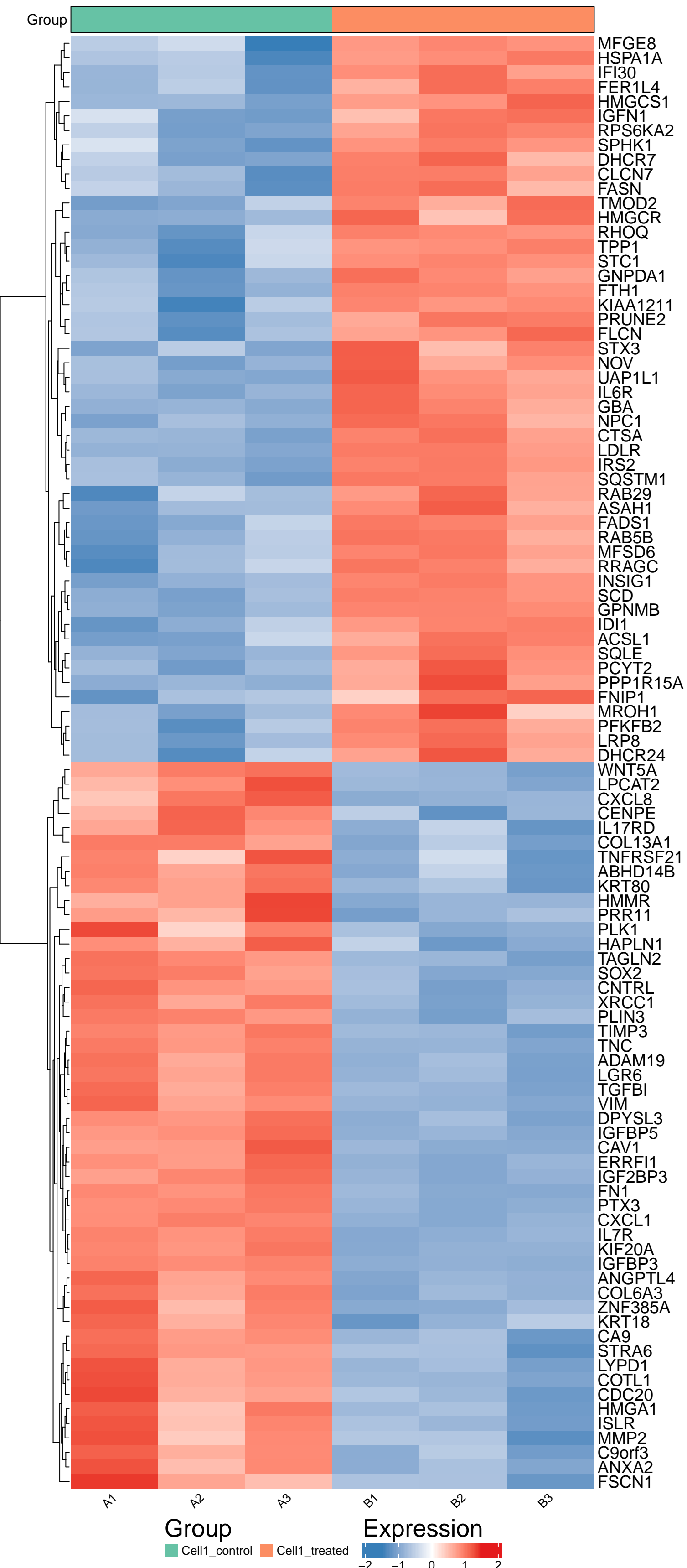

Supplement: Supplementary file 10 — Source data Fig. 4 [file 44319_2025_660_MOESM10_ESM.zip › Figure 5/5B/A375_SKMEL-103_common_top100_genesCell1_heatmap.pdf]

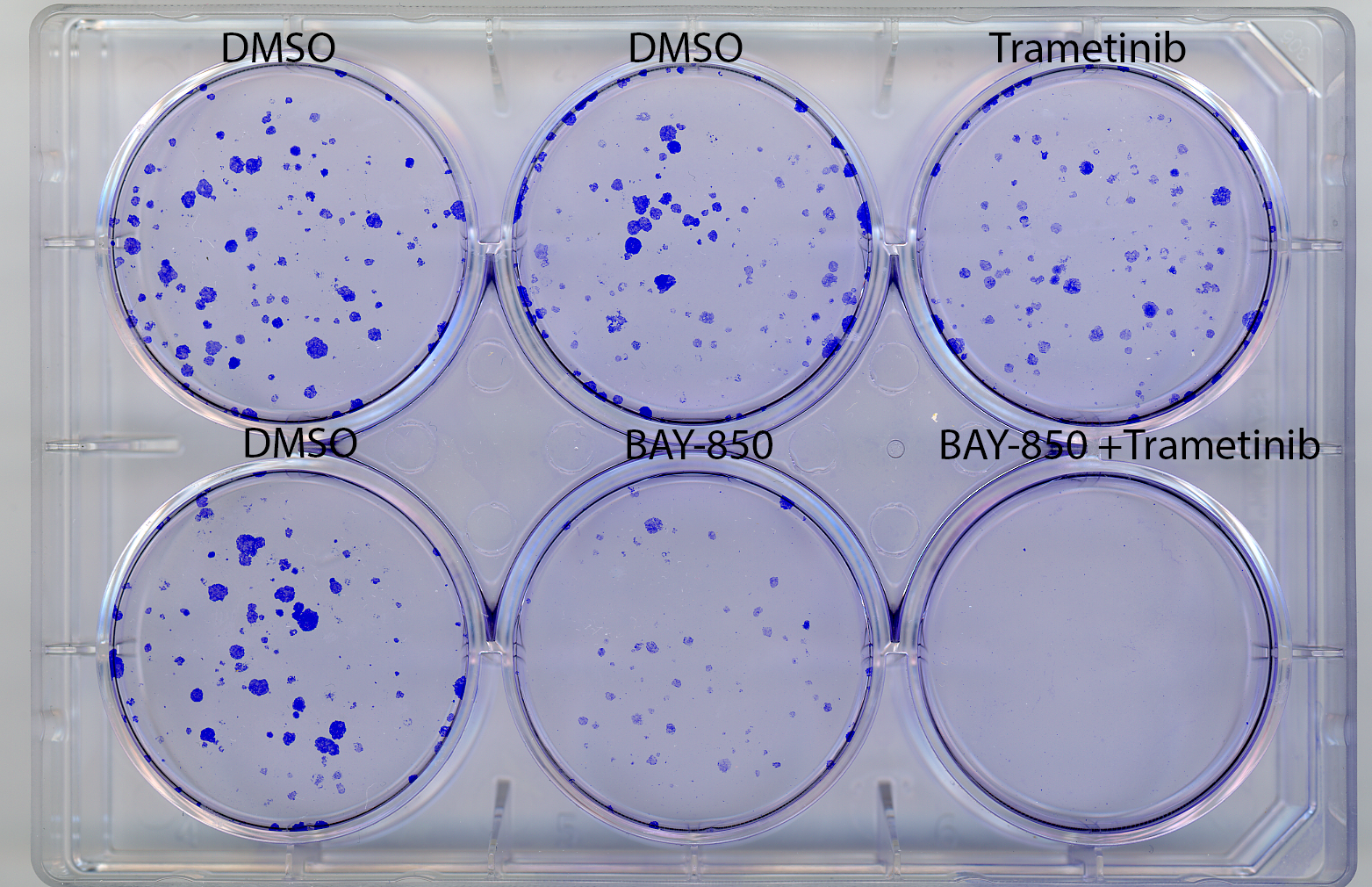

Supplement: Supplementary file 11 — Source data Fig. 5 [file 44319_2025_660_MOESM11_ESM.zip › Figure 6/6G/SKMEL-103_combination_clonogenic.tif]

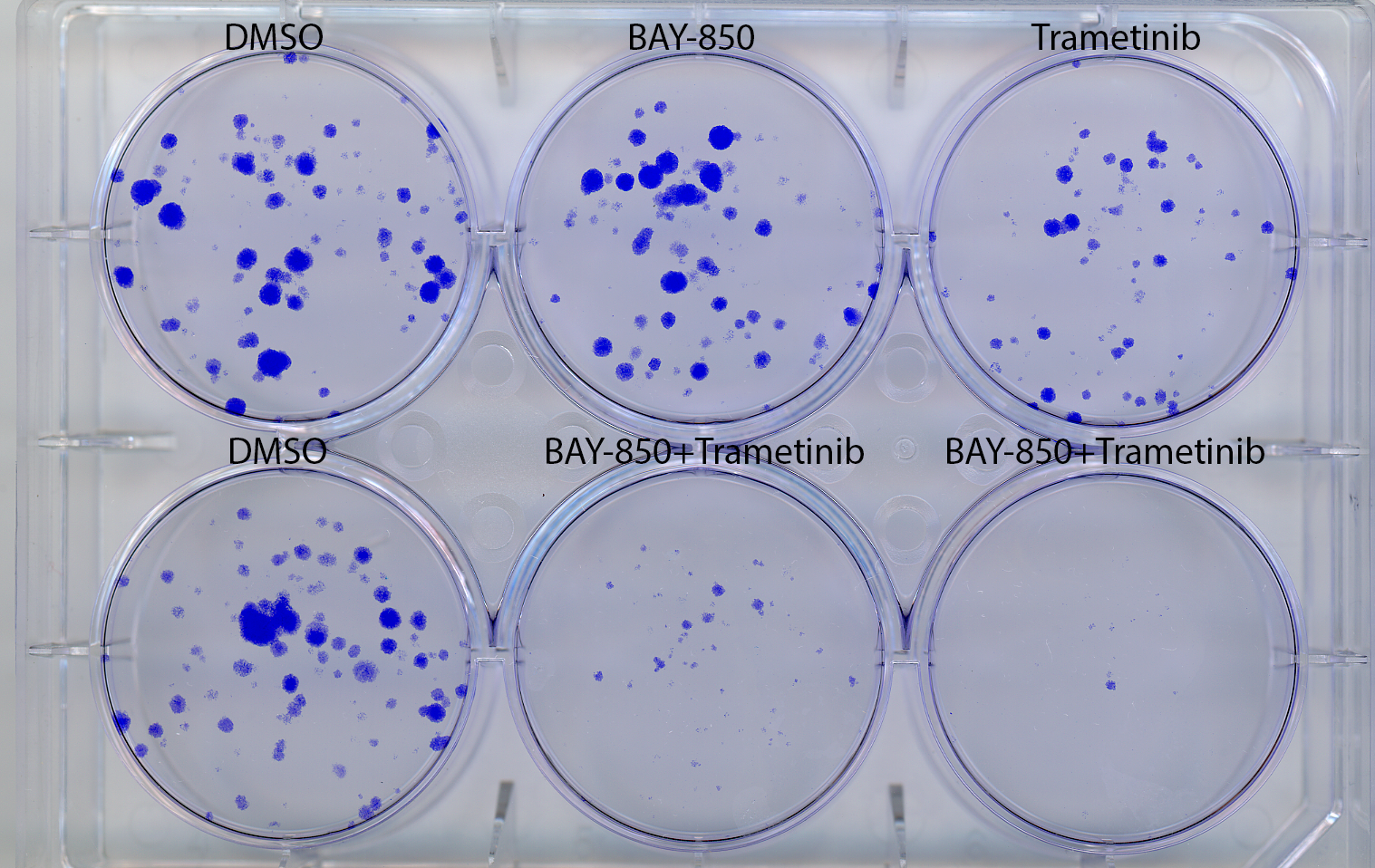

Supplement: Supplementary file 11 — Source data Fig. 5 [file 44319_2025_660_MOESM11_ESM.zip › Figure 6/6G/A375_combination_clonogenic.tif]

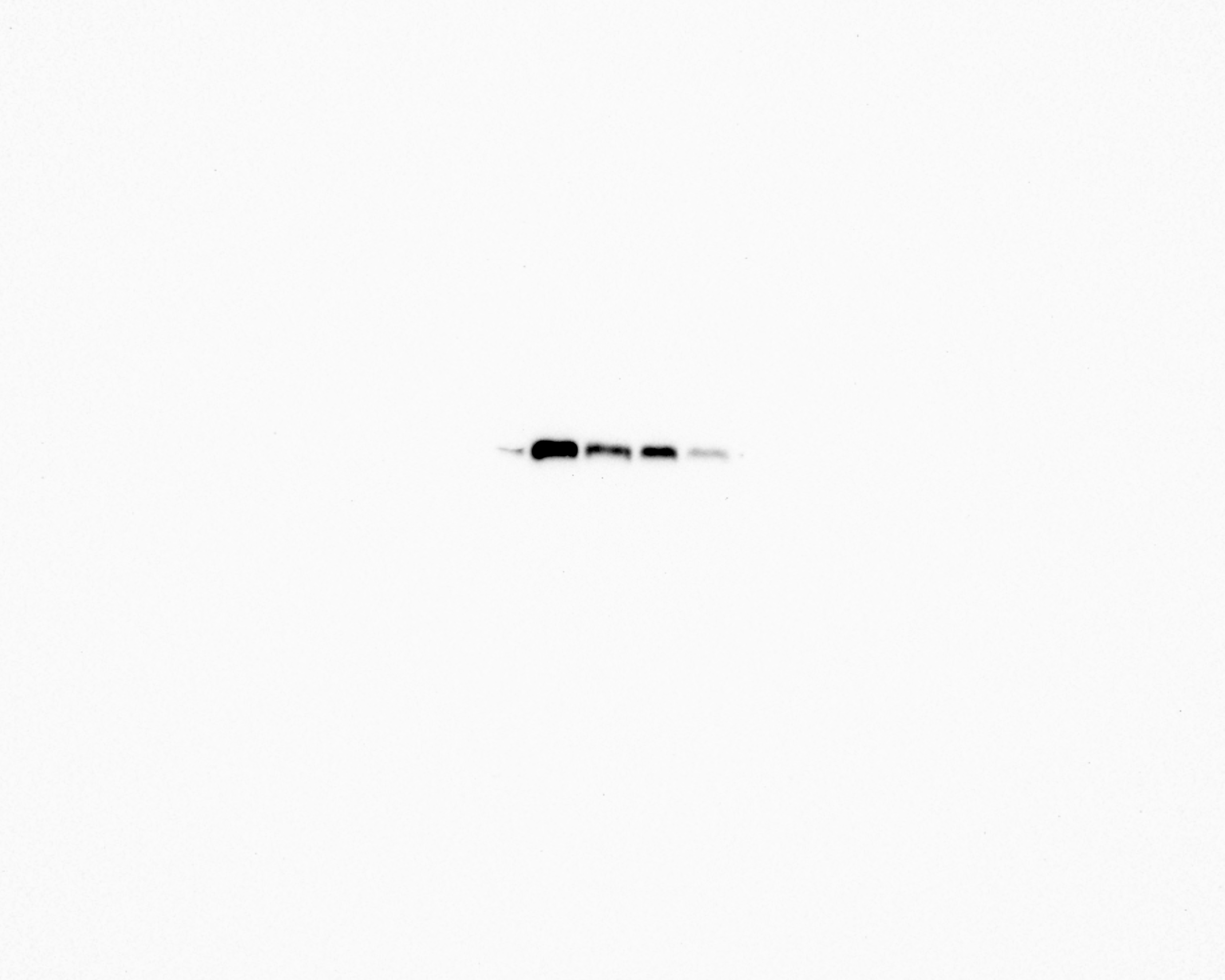

Supplement: Supplementary file 11 — Source data Fig. 5 [file 44319_2025_660_MOESM11_ESM.zip › Figure 6/6B/GPX4_BAY850 Treated_A375 _SKMEL-103.tif]

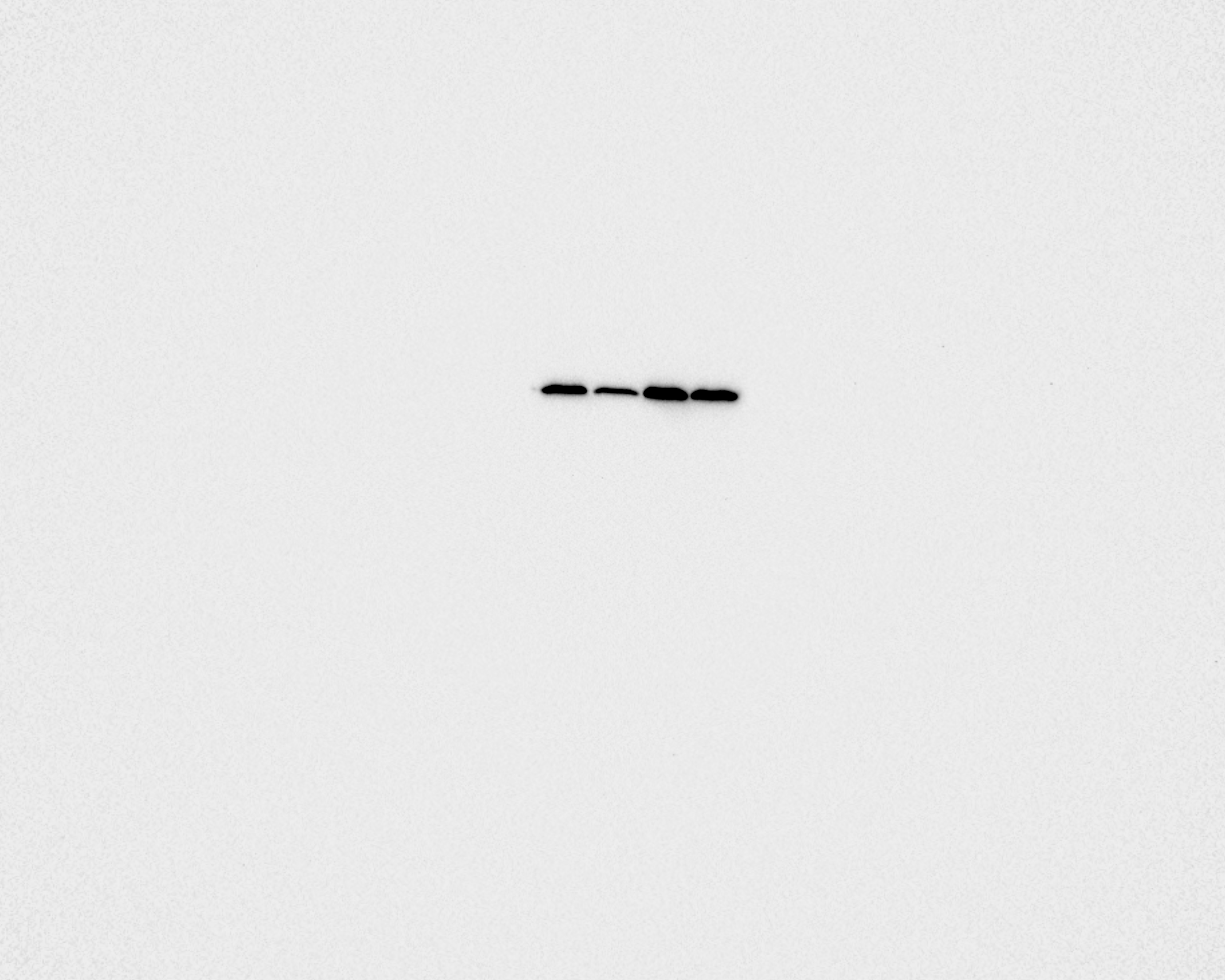

Supplement: Supplementary file 11 — Source data Fig. 5 [file 44319_2025_660_MOESM11_ESM.zip › Figure 6/6B/Actin for GPX4_BAY850 Treated_A375_SKMEL103.tif]

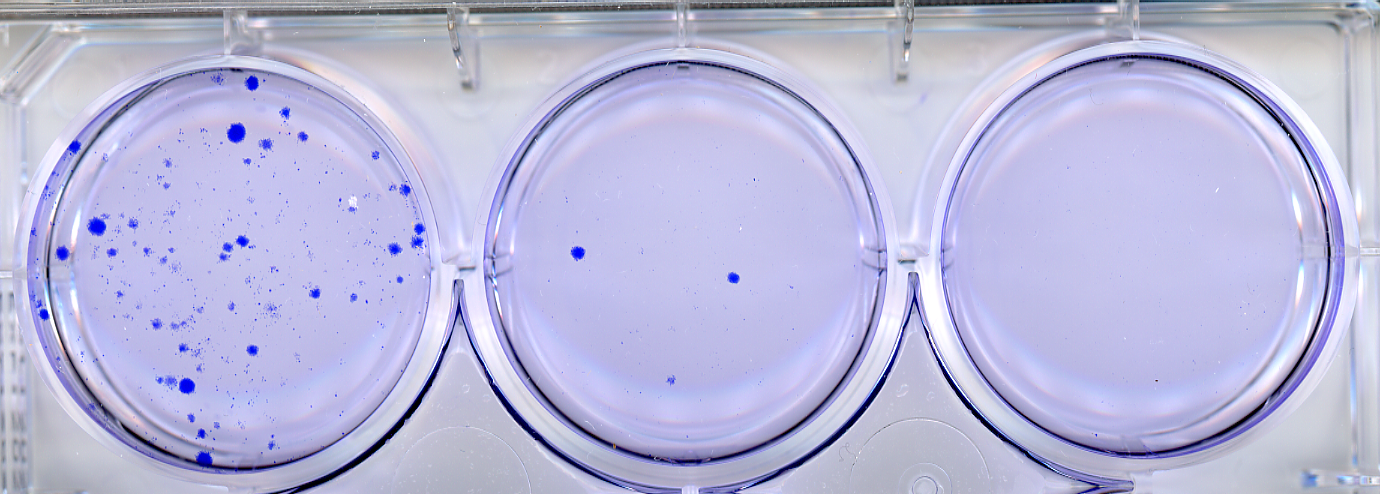

Supplement: Supplementary file 11 — Source data Fig. 5 [file 44319_2025_660_MOESM11_ESM.zip › Figure 6/6E/SKMEL-103_erastin trt.tif]

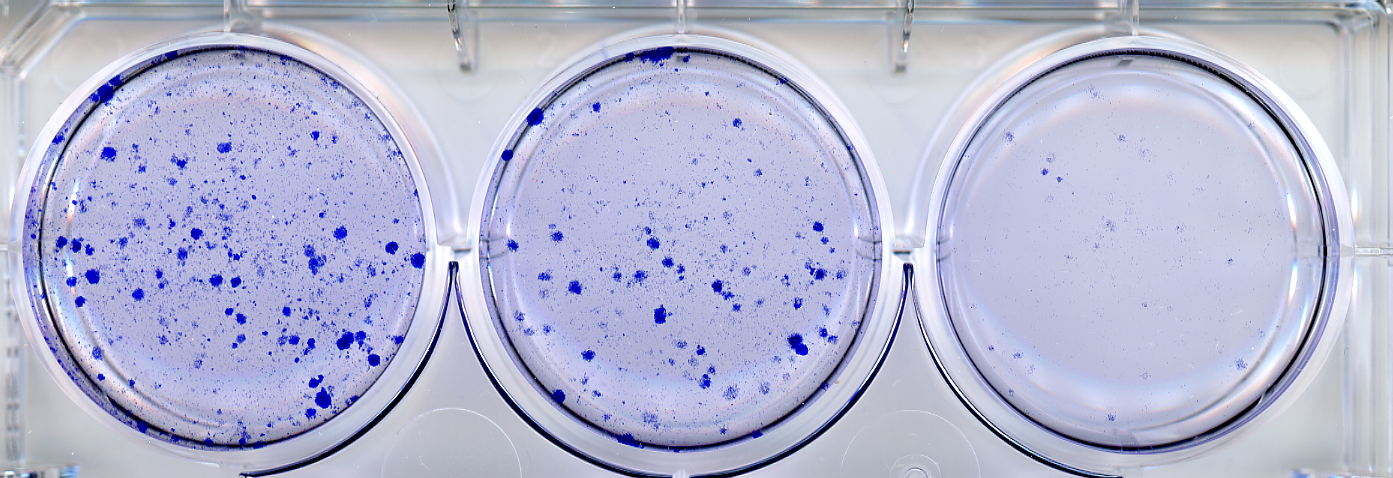

Supplement: Supplementary file 11 — Source data Fig. 5 [file 44319_2025_660_MOESM11_ESM.zip › Figure 6/6E/A375_Erastin trt.tif]

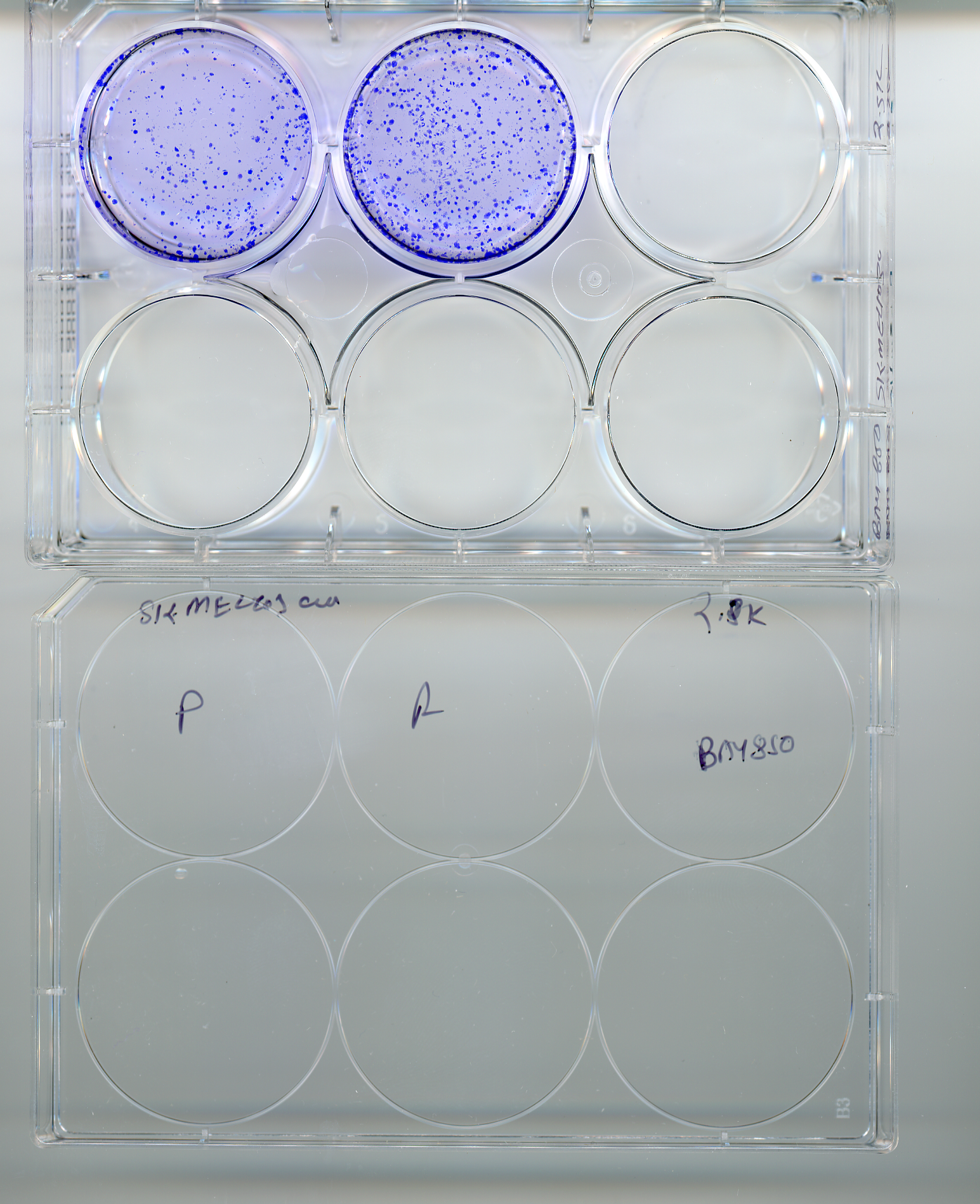

Supplement: Supplementary file 12 — Source data Fig. 6 [file 44319_2025_660_MOESM12_ESM.zip › Figure 6/6A/SK-MEL-103_BAY Res_BAY-850.tif]

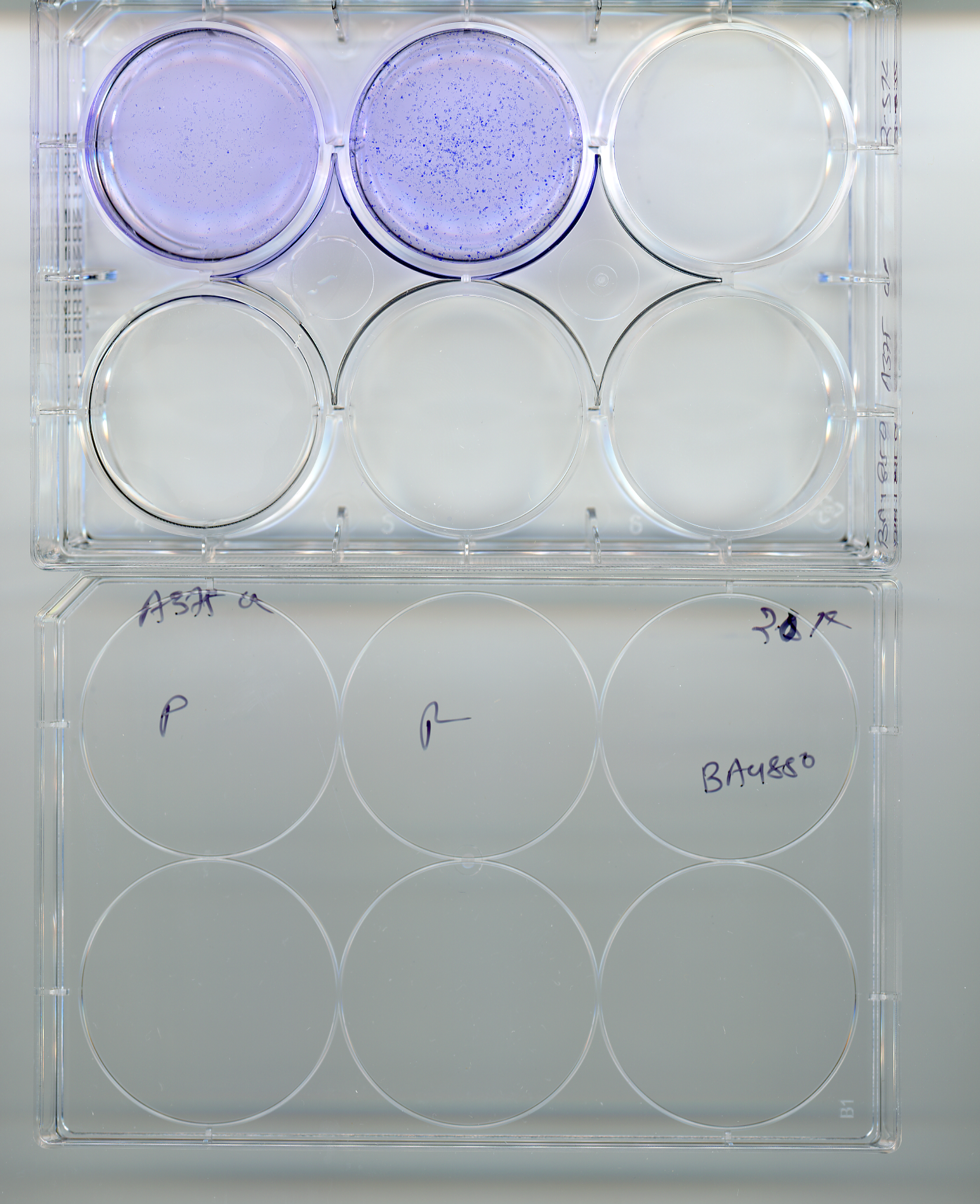

Supplement: Supplementary file 12 — Source data Fig. 6 [file 44319_2025_660_MOESM12_ESM.zip › Figure 6/6A/A375_BAY Res_BAY-850.tif]

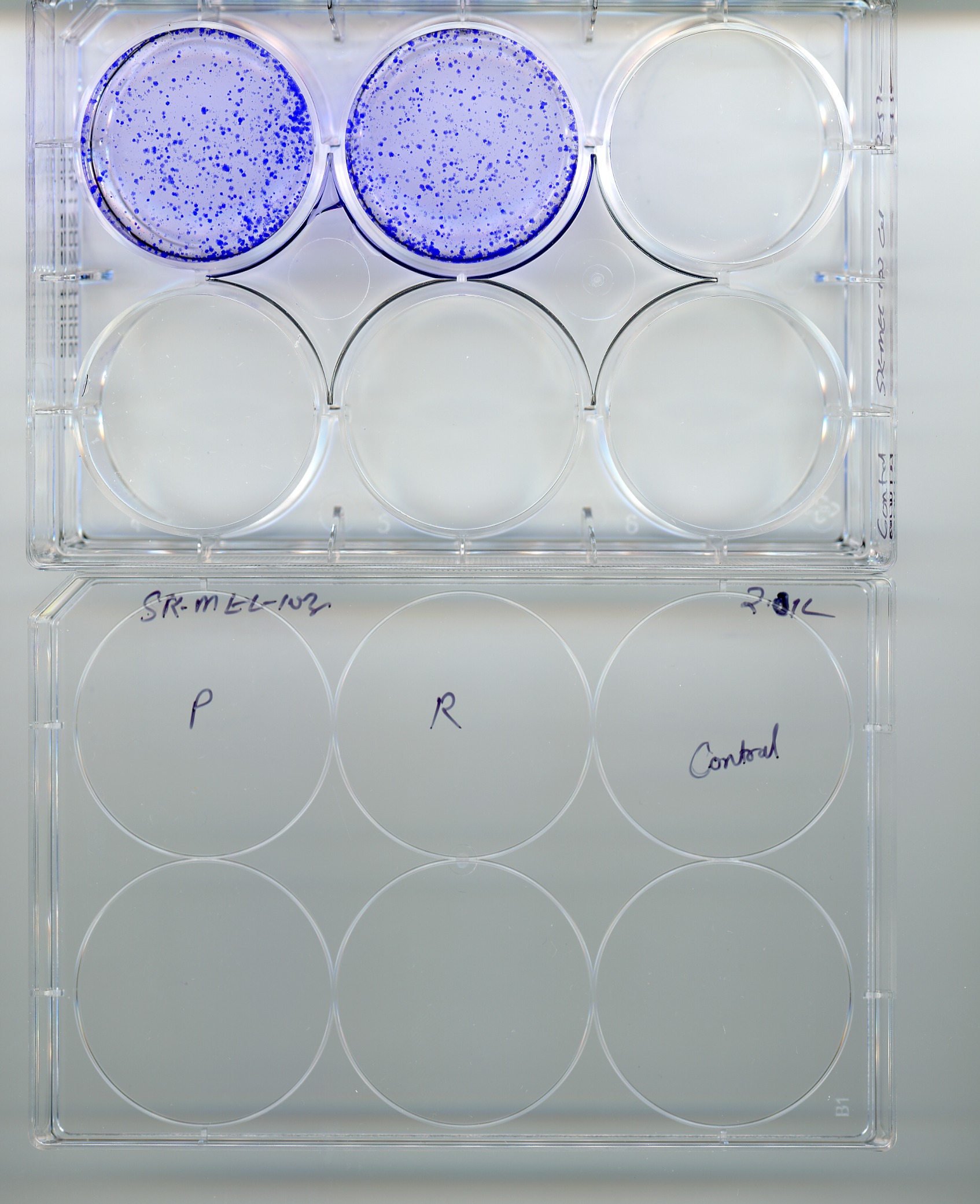

Supplement: Supplementary file 12 — Source data Fig. 6 [file 44319_2025_660_MOESM12_ESM.zip › Figure 6/6A/SK-MEL-103_BAY Res_DMSO.tif]

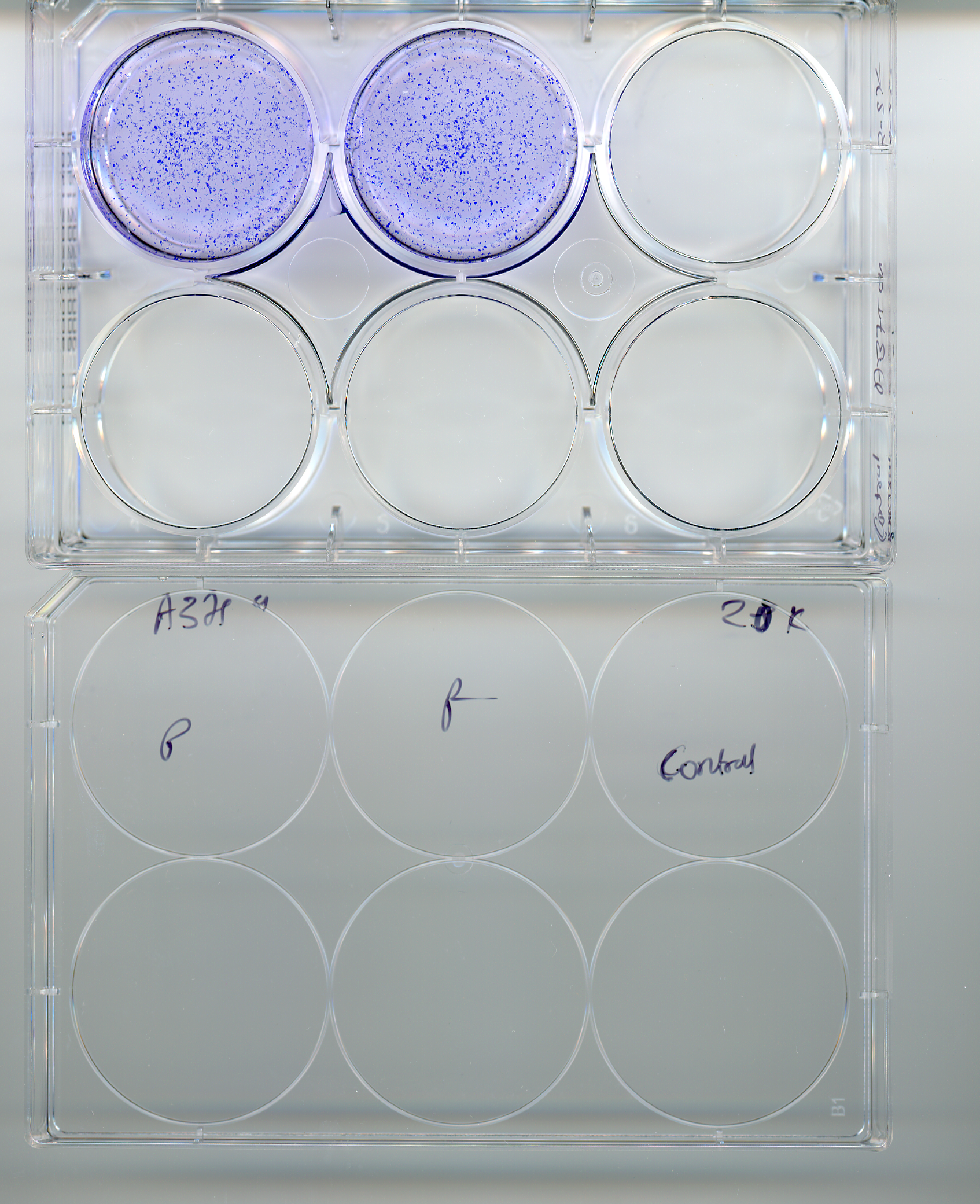

Supplement: Supplementary file 12 — Source data Fig. 6 [file 44319_2025_660_MOESM12_ESM.zip › Figure 6/6A/A375_BAY Res_DMSO.tif]

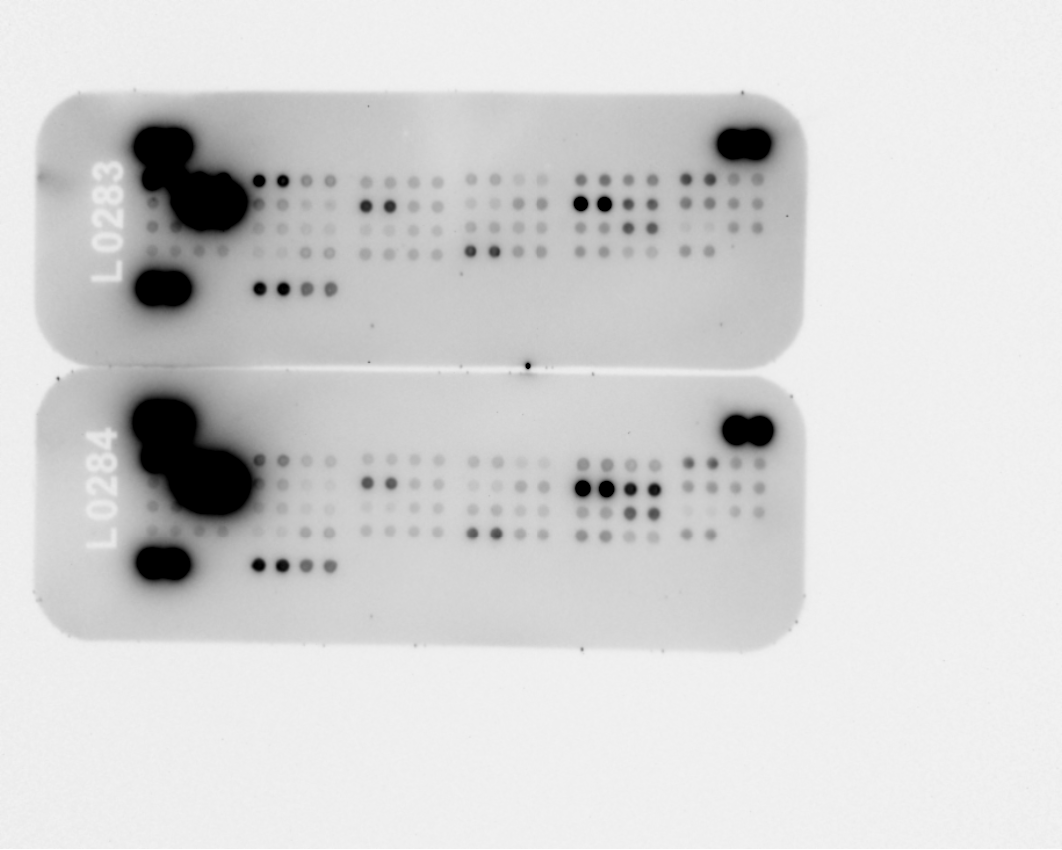

Supplement: Supplementary file 12 — Source data Fig. 6 [file 44319_2025_660_MOESM12_ESM.zip › Figure 6/6B/SK-MEL-103 BAY850 R Cells RTK.tif]

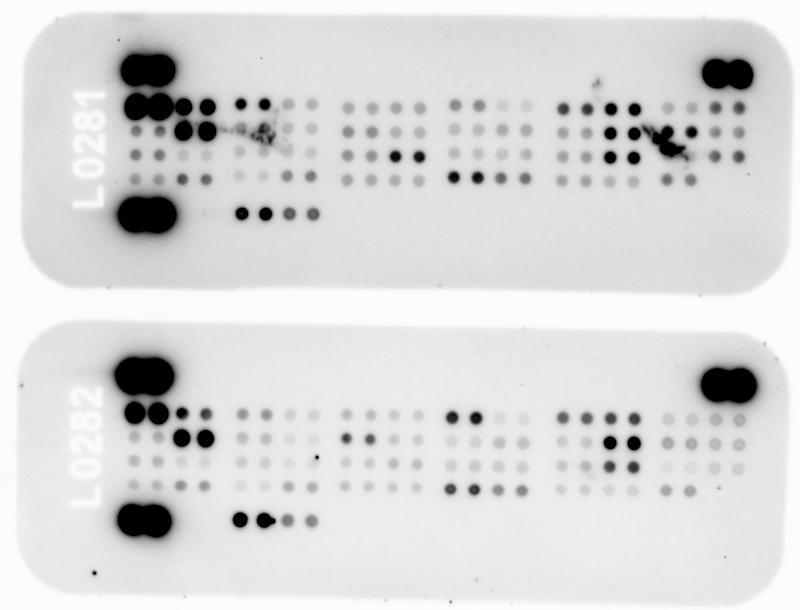

Supplement: Supplementary file 12 — Source data Fig. 6 [file 44319_2025_660_MOESM12_ESM.zip › Figure 6/6B/A375 BAY850 R Cells RTK.tif]

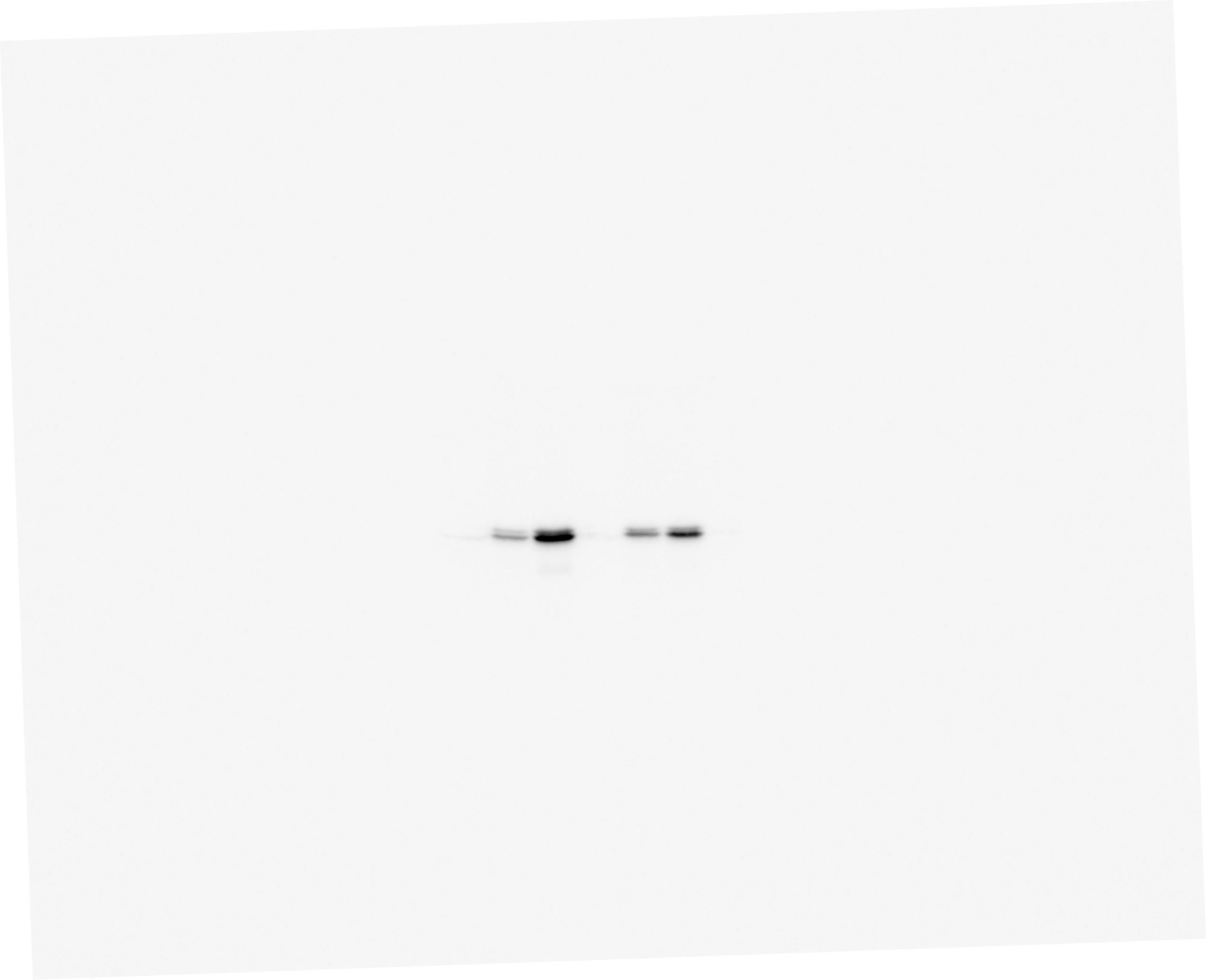

Supplement: Supplementary file 12 — Source data Fig. 6 [file 44319_2025_660_MOESM12_ESM.zip › Figure 6/6E/pERK1:2_BAY850 Resistant_A375 & SKMEL-103 Cells.tif]

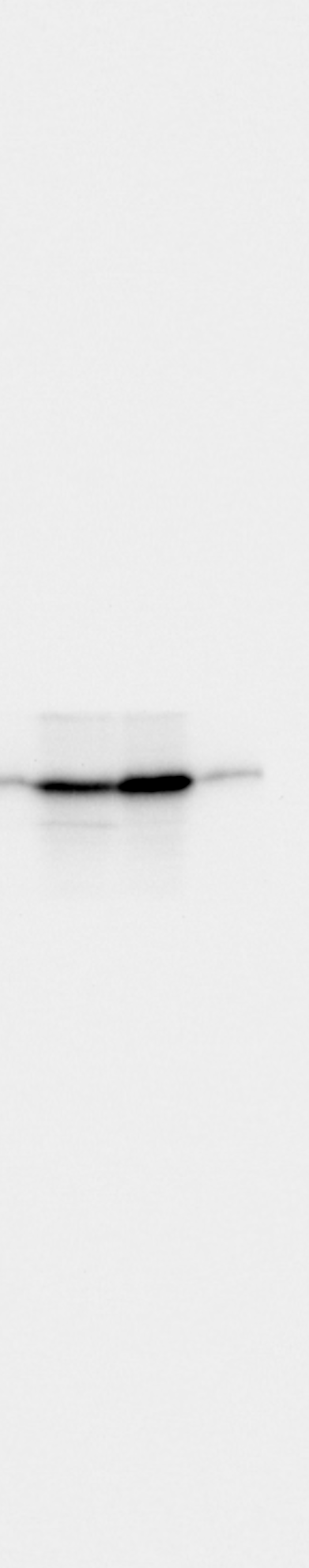

Supplement: Supplementary file 12 — Source data Fig. 6 [file 44319_2025_660_MOESM12_ESM.zip › Figure 6/6E/pMEK1:2_BAY850 Resistant_ SKMEL-103 Cells.tif]

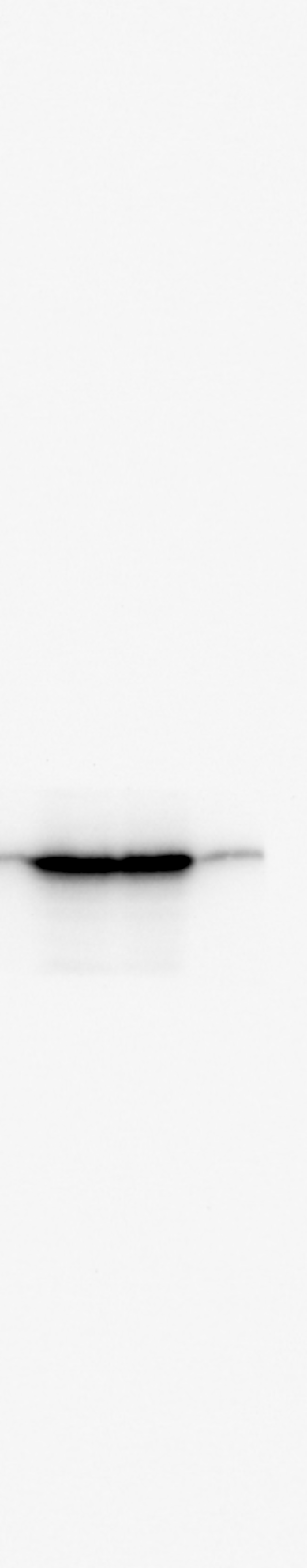

Supplement: Supplementary file 12 — Source data Fig. 6 [file 44319_2025_660_MOESM12_ESM.zip › Figure 6/6E/Total MEK1:2_BAY850 Resistant_SKMEL-103 Cells.tif]

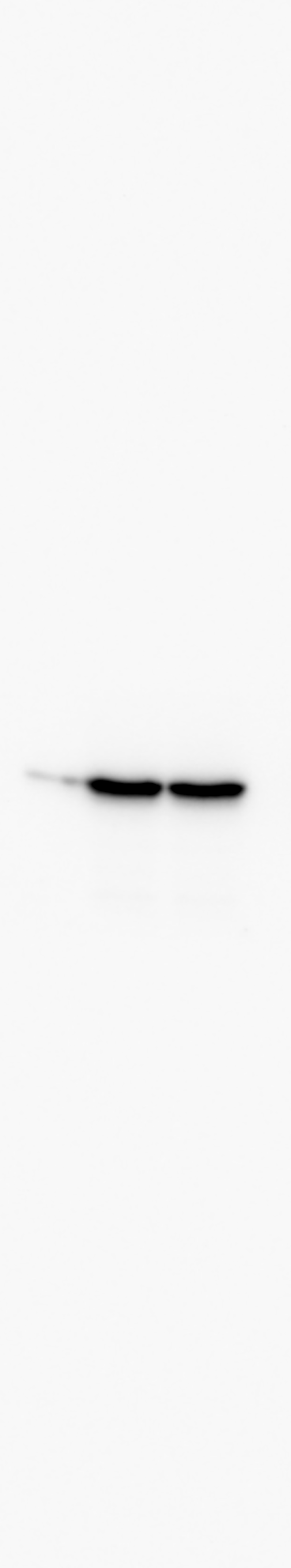

Supplement: Supplementary file 12 — Source data Fig. 6 [file 44319_2025_660_MOESM12_ESM.zip › Figure 6/6E/Total MEK12_BAY850_Resistant_A375 cells.tif]

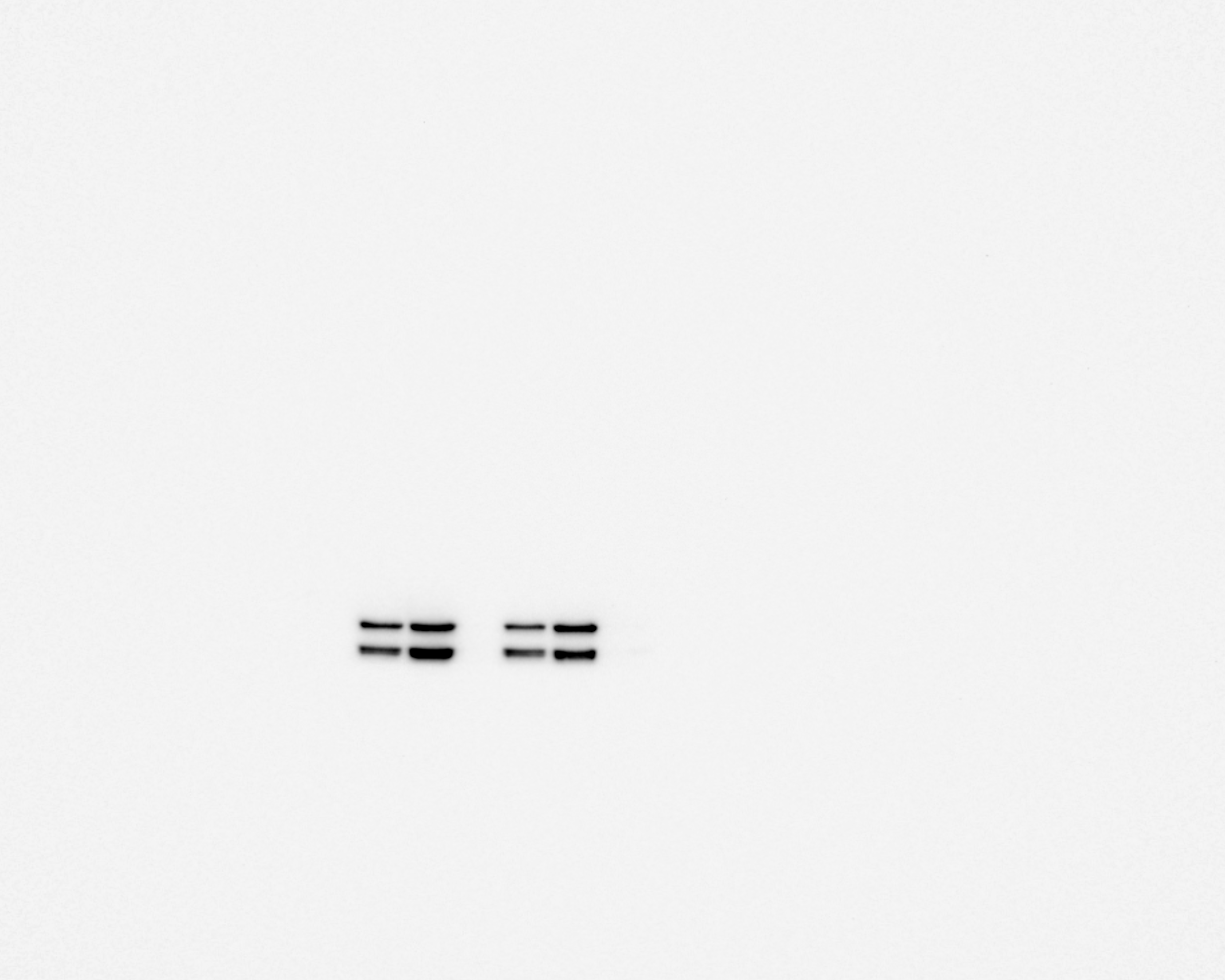

Supplement: Supplementary file 12 — Source data Fig. 6 [file 44319_2025_660_MOESM12_ESM.zip › Figure 6/6E/pROR1_BAY850 Resistant_A375 & SKMEL-103 Cells.tif]

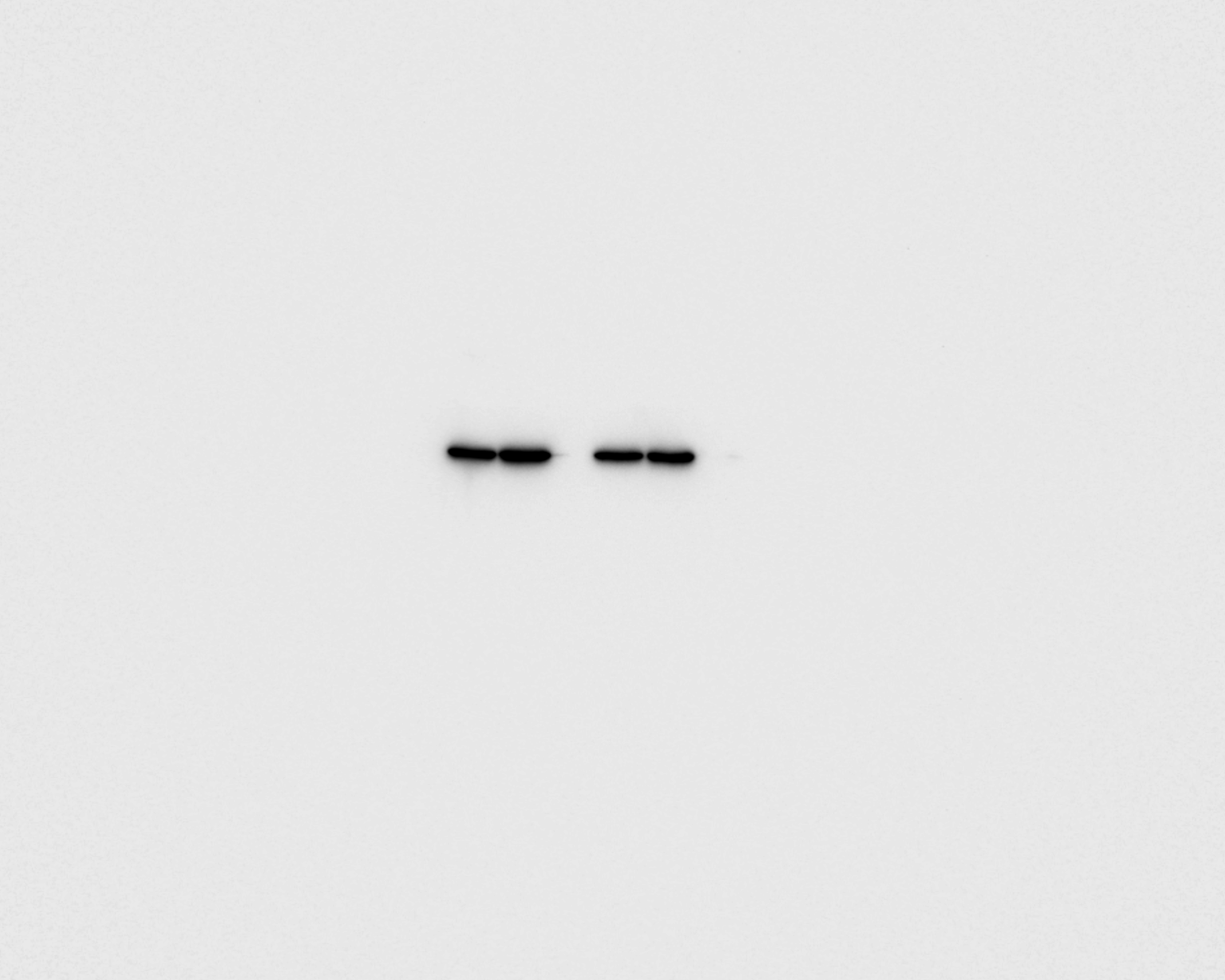

Supplement: Supplementary file 12 — Source data Fig. 6 [file 44319_2025_660_MOESM12_ESM.zip › Figure 6/6E/Total-ROR1_BAY850 Resistant_A375 & SKMEL-103 Cells.tif]

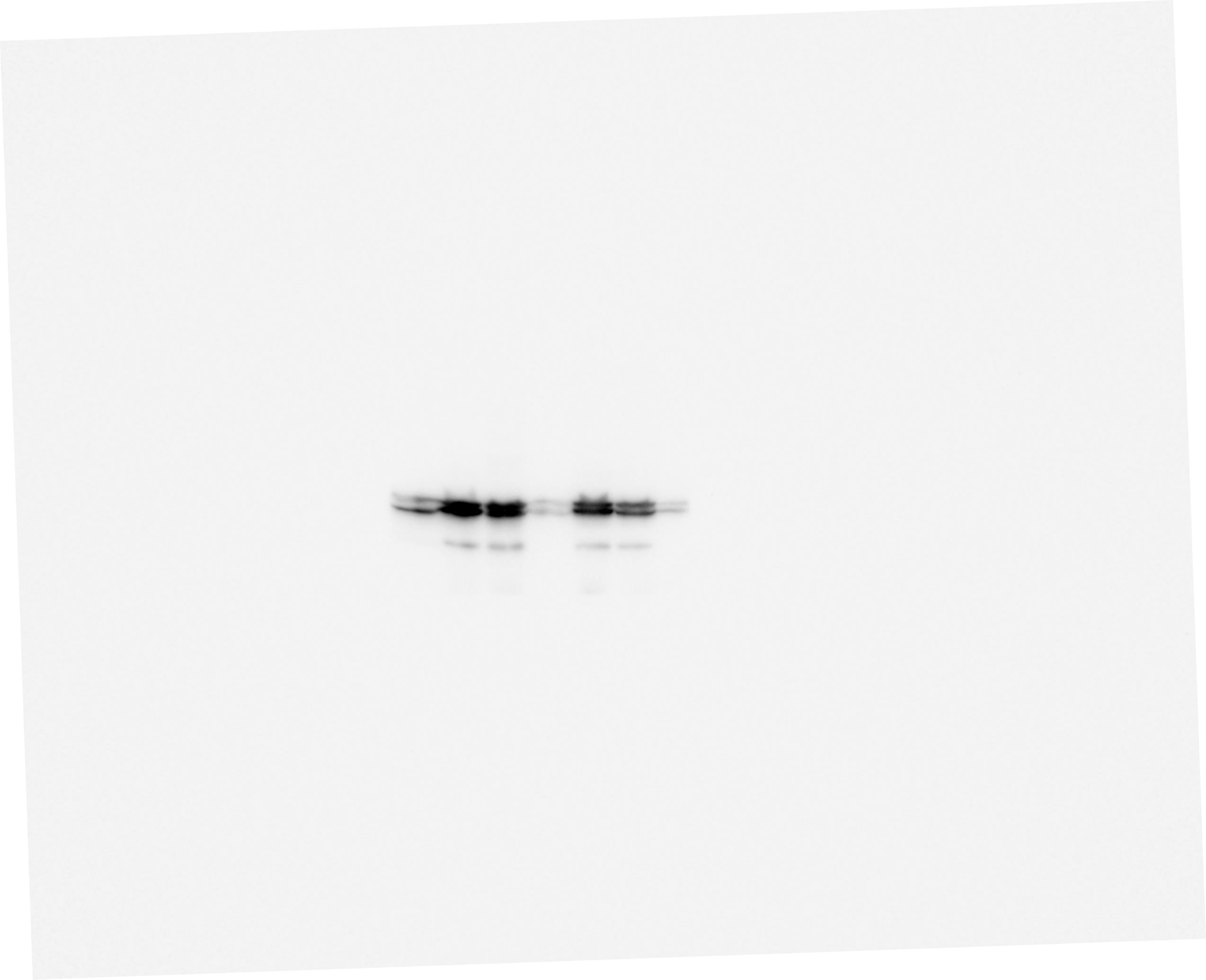

Supplement: Supplementary file 12 — Source data Fig. 6 [file 44319_2025_660_MOESM12_ESM.zip › Figure 6/6E/Total ERK1:2_BAY850 Resistant_A375 & SKMEL-103 Cells.tif]

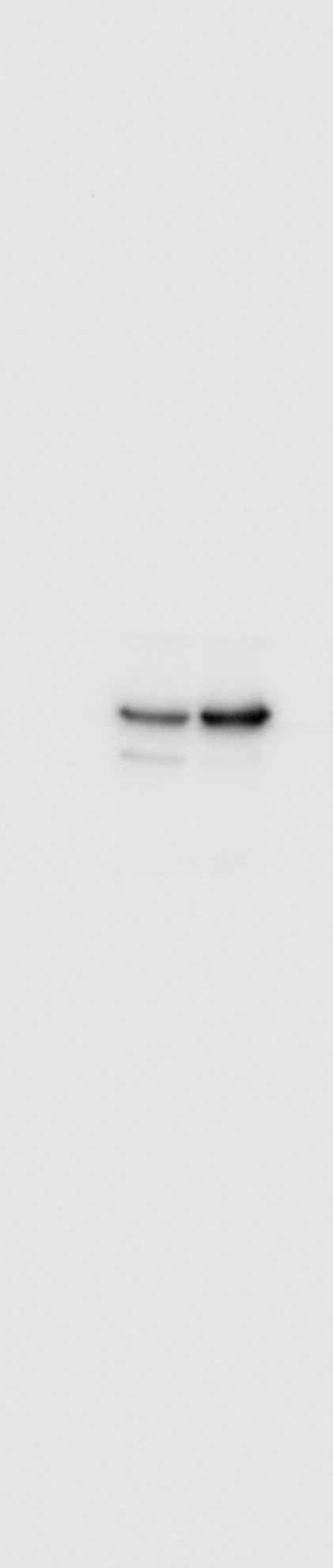

Supplement: Supplementary file 12 — Source data Fig. 6 [file 44319_2025_660_MOESM12_ESM.zip › Figure 6/6E/pMEK12_BAY850_Resistant_A375 Cells.tif]

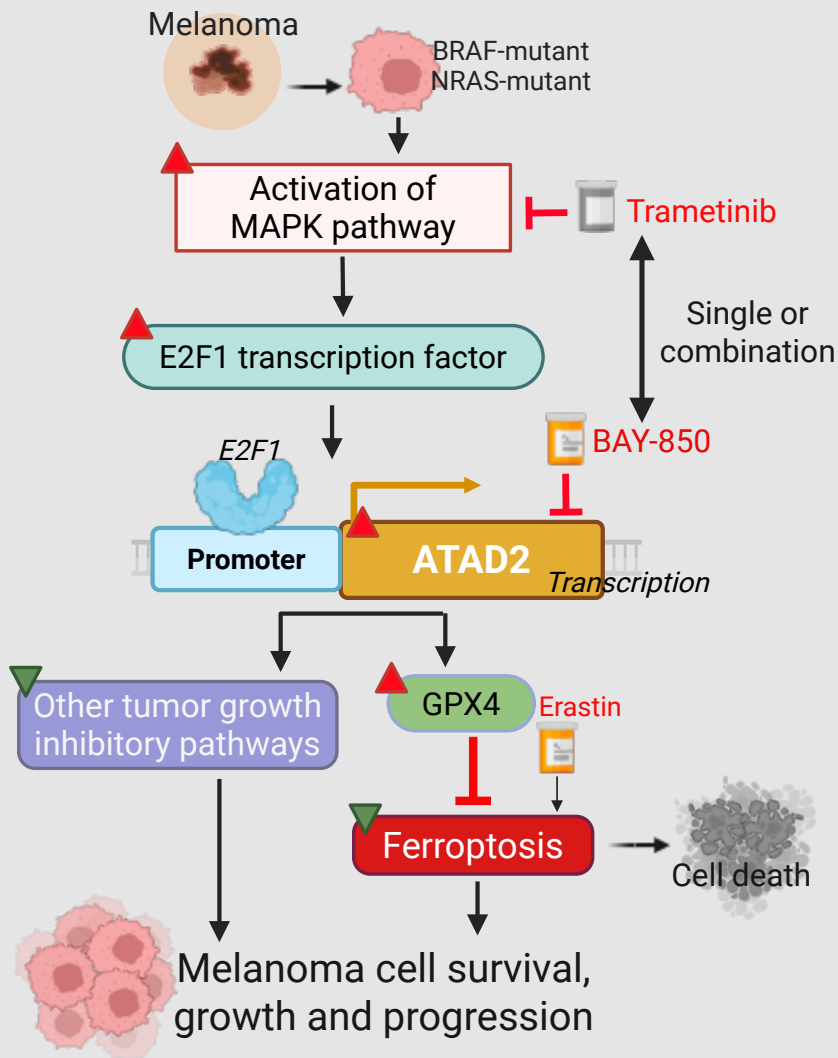

Supplement: Supplementary file 13 — Source data Fig. 7 [file 44319_2025_660_MOESM13_ESM.zip › Figure 7/Model.pdf]
