# Supplementary material for: ATAD2 drives melanoma growth and progression and inhibits ferroptosis
Source: EMBO Rep. 2025 Dec 2;27(2):501–32. doi: 10.1038/s44319-025-00660-w (PMC12852765; doi:10.1038/s44319-025-00660-w)
Supplement: Supplementary file 9 — Source data Fig. 3 [file 44319_2025_660_MOESM9_ESM.zip › Figure 3/3A/Schematics.pdf]

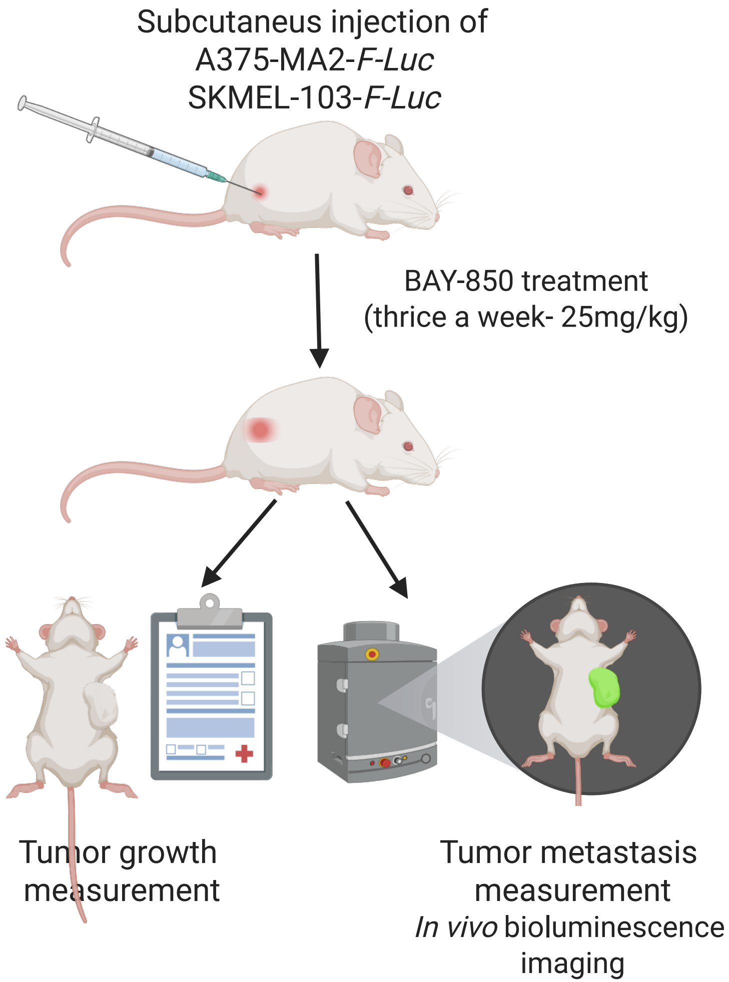
Subcutaneus injection of
A375-MA2-*F-Luc*
SKMEL-103-*F-Luc*
BAY-850 treatment
(thrice a week- 25mg/kg)
Tumor growth
measurement
Tumor metastasis
measurement
In vivo bioluminescence
imaging
