# Supplementary material for: ATAD2 drives melanoma growth and progression and inhibits ferroptosis
Source: EMBO Rep. 2025 Dec 2;27(2):501–32. doi: 10.1038/s44319-025-00660-w (PMC12852765; doi:10.1038/s44319-025-00660-w)
Supplement: Supplementary file 14 — Expanded View Figures [file 44319_2025_660_MOESM14_ESM.pdf]

Expanded View Figures

Figure EV1. ATAD2 is overexpressed in patient-derived melanoma samples and is regulated by MAPK pathways.

(**A**) The Gene Expression Profiling Interactive Analysis (GEPIA) data was used for plotting mRNA expression of family IV of bromodomain-containing members. mRNA expression of BRPF1, BRPF2, BRPF3, BRD7, BRD9, and ATAD2b is shown in melanoma patient samples ($n = 461$) and normal skin samples ($n = 558$). (**B**, **C**) Human Protein Atlas showing ATAD2 protein expression intensity in different patient samples. (**D**) List of transcription factors with predicted DNA binding sites on the *ATAD1* promoter DNA sequence (1 kb upstream from the transcription start site) generated using PROMO search. (**E**) TCGA melanoma data showing the correlation between ATAD2 and E2F1 mRNA expression. (**F**) In the indicated melanoma cell lines ATAD2 mRNA expression was measured using RT-qPCR and plotted relative to primary human fibroblast cells. *ACTINB* was used as a normalization control (left) and ATAD2 protein expression in the shown cells was measured via western blotting. ACTINB was used as loading control (right). (**G**) The indicated cell lines were treated with 5μM of BAY-850 for 3 days, and viability was assessed by 3-(4,5-dimethylthiazol-2-yl)-2,5-diphenyltetrazolium bromide (MTT) assay. Relative cell viability in treated condition relative to DMSO-treated condition is presented, ns $P = 0.2244$, **$P = 0.0013$, ****$P = < 0.0001$, ****$P = < 0.0001$, from left to right. (**A**) P value was calculated using ANOVA (Analysis of Variance) for differential expression analysis between tumor and normal tissues. (**G**) P value was calculated using unpaired Student's t test using three independent replicates.

© The Author(s)

A

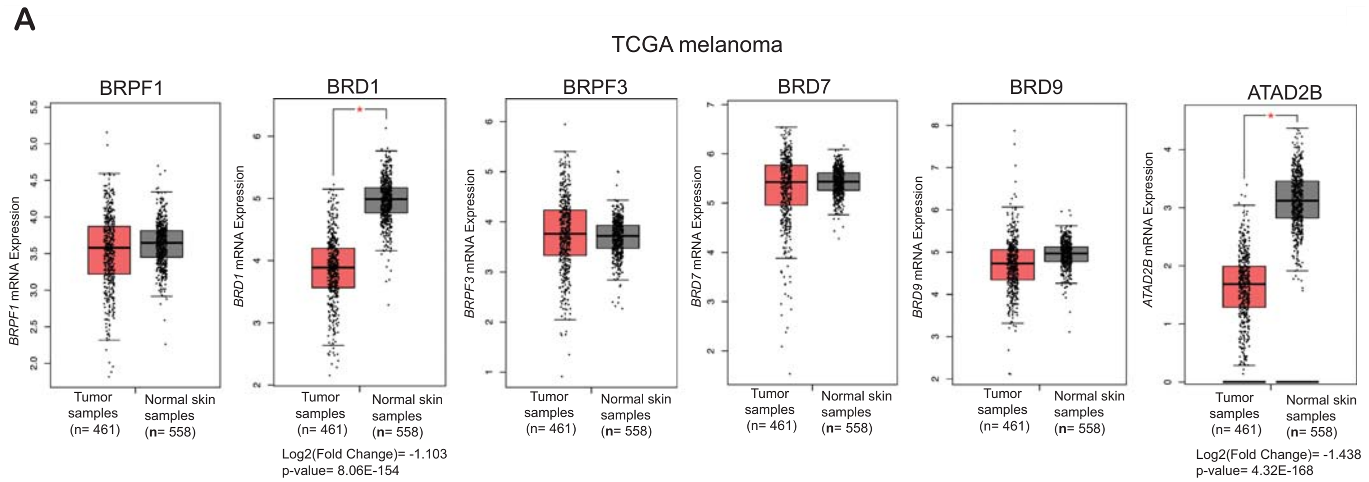

B

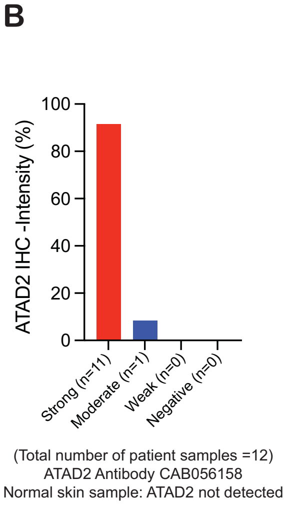

C

Human Protein Atlas
ATAD2 IHC- intensity

(Total number of patient samples =12)

Patient id	Intensity
2534	Strong
2383	Strong
3140	Strong
4028	Strong
2317	Strong
2136	Strong
2598	Strong
3611	Strong
3192	Strong
3060	Strong
4029	Strong
3887	Moderate

D

Factors predicted by PROMO for 1KB promoter region of ATAD2

USF1
GATA-1
GR-beta
TBP
GR
GR-alpha
RFX1
p53
AP-2alphaA
AP-2alphaB
NF-1
c-Ets-2
YY1
Pax-5
MZF-1
E2F-1
GCF
ENKTF-1
Sp1
E2F
CTF
TCF-4
HNF-3alpha
T3R-beta1

E

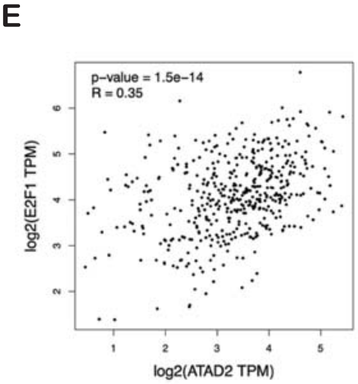

F

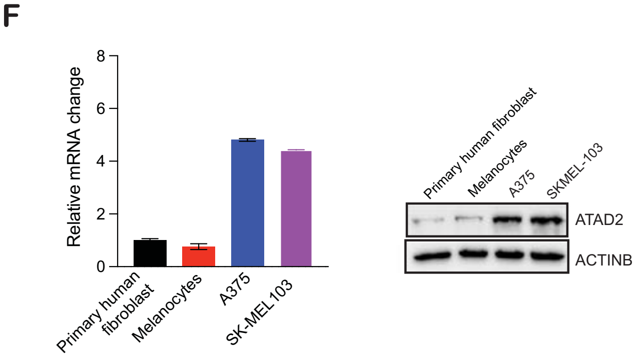

G

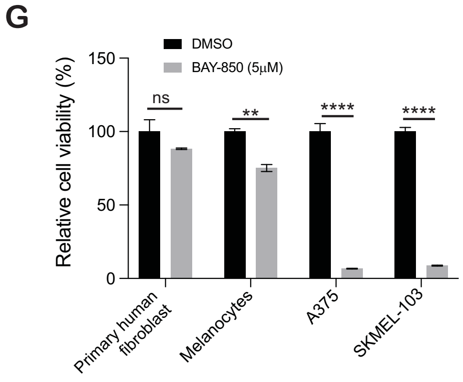

 © The Author(s)

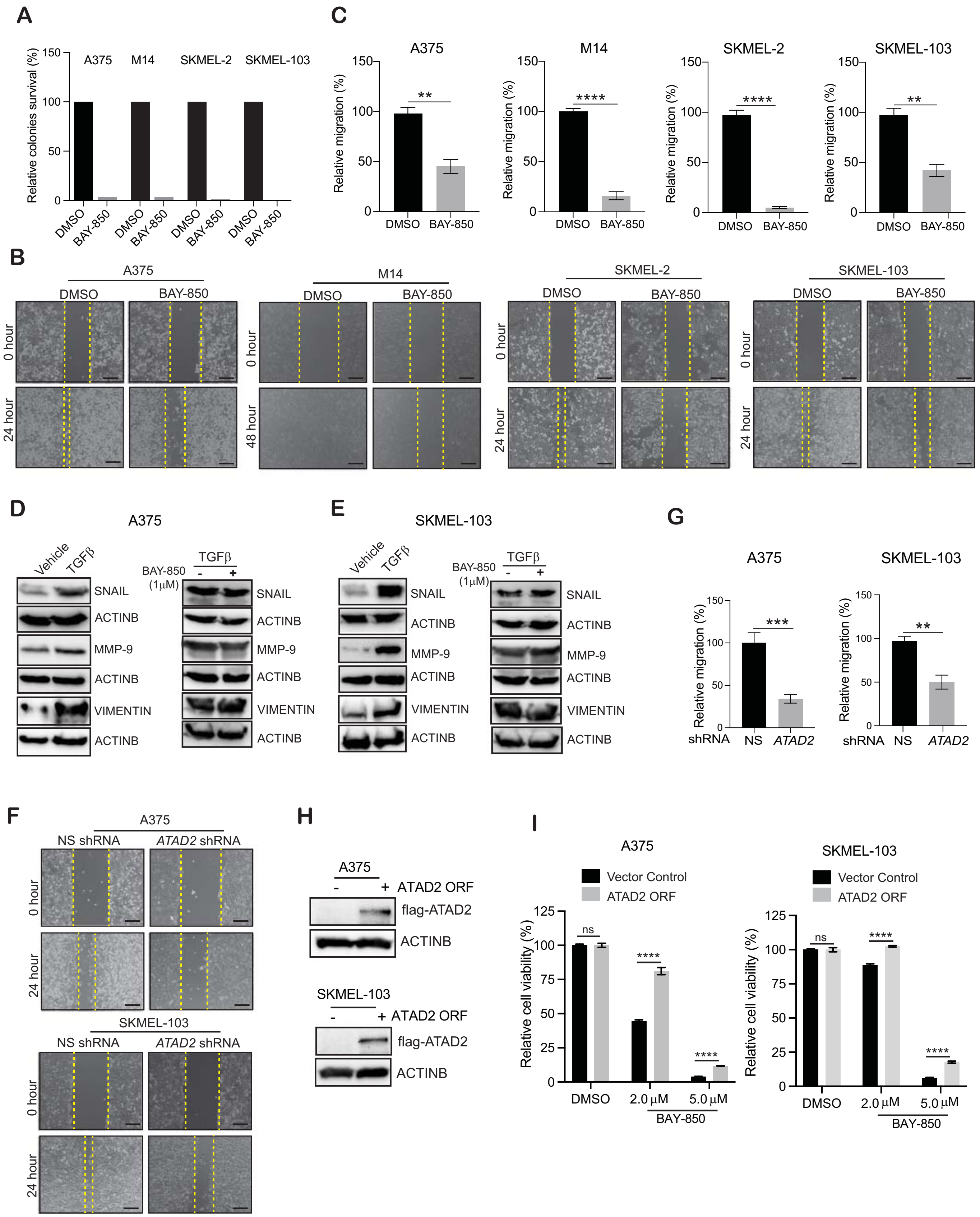
A
Relative colonies survival (%)
A375
M14
SKMEL-2
SKMEL-103
DMSO
BAY-850
C
A375
M14
SKMEL-2
SKMEL-103
Relative migration (%)
DMSO
BAY-850
B
0 hour
24 hour
48 hour
D
A375
Vehicle
TGFβ
BAY-850 (1μM)
SNAIL
ACTINB
MMP-9
VIMENTIN
E
SKMEL-103
G
shRNA
NS
ATAD2
F
NS shRNA
ATAD2 shRNA
H
ATAD2 ORF
flag-ATAD2
I
Vector Control
ATAD2 ORF
Relative cell viability (%)
2.0 μM
5.0 μM
ns

Figure EV2. ATAD2 inhibition suppresses melanoma growth and progression.

(**A**) The indicated melanoma cell lines were treated with 5 μM of BAY-850 for 2–4 weeks. Cell survival was measured using clonogenic assays. Relative percentage colony survival (%) shown in Fig. 2B is presented here. (**B**) Migration was analyzed in a wound-healing assay for melanoma cell lines treated with either DMSO or 5μM of BAY-850. Yellow dotted lines in the scratch assay images indicate the wound margins. Representative images are shown; scale bar, 200 μm. (**C**) Relative migration (%) in BAY-850 treated relative to the DMSO-treated cells is plotted for the experiment shown in (**B**), $^{**}P = 0.0021$, $^{****}P = < 0.0001$, $^{****}P = < 0.0001$, $^{**}P = 0.0040$, from left to right. (**D**, **E**) The indicated melanoma cell lines were serum starved for 12 h and were then exposed with Recombinant Human TGF-beta1 protein by adding to the media at concentration of 10 ng/mL for 48 h with or without 1 μM BAY-850 for 48 h. Indicated proteins were analyzed in shown condition for A375 and SKMEL-103 melanoma cell lines. ACTINB was used as the loading control. (**F**) Migration was analyzed in a wound-healing assay for melanoma cell lines expressing (NS) shRNAs or *ATAD2* shRNAs. Yellow dotted lines in the scratch assay images indicate the wound margins. Representative images are shown; scale bar, 200 μm. (**G**) Relative migration (%) in *ATAD2* shRNA expression cells relative to the control NS shRNA expressing cells shown in (**F**) is plotted. $^{***}P = 0.0010$, $^{**}P = 0.0076$, from left to right. (**H**) Indicated melanoma cell lines overexpressing either vector control or ATAD2-ORF were immune-blotted for the shown proteins confirming ATAD2 overexpression. (**I**) Indicated melanoma cell lines overexpressing either vector control or ATAD2-ORF were treated with BAY-850 at the shown concentration and cell viability was measured using 3-(4,5-dimethylthiazol-2-yl)-2,5-diphenyltetrazolium bromide (MTT) assay. Relative cell viability in the shown condition is presented, ns $P = > 0.9999$, $^{****}P = < 0.0001$, $^{****}P = < 0.0001$, ns $P = > 0.9999$, $^{****}P = < 0.0001$, $^{****}P = < 0.0001$, from left to right. (**B**, **F**, **I**) P value was calculated using unpaired Student's t test using three independent replicates.

 © The Author(s)

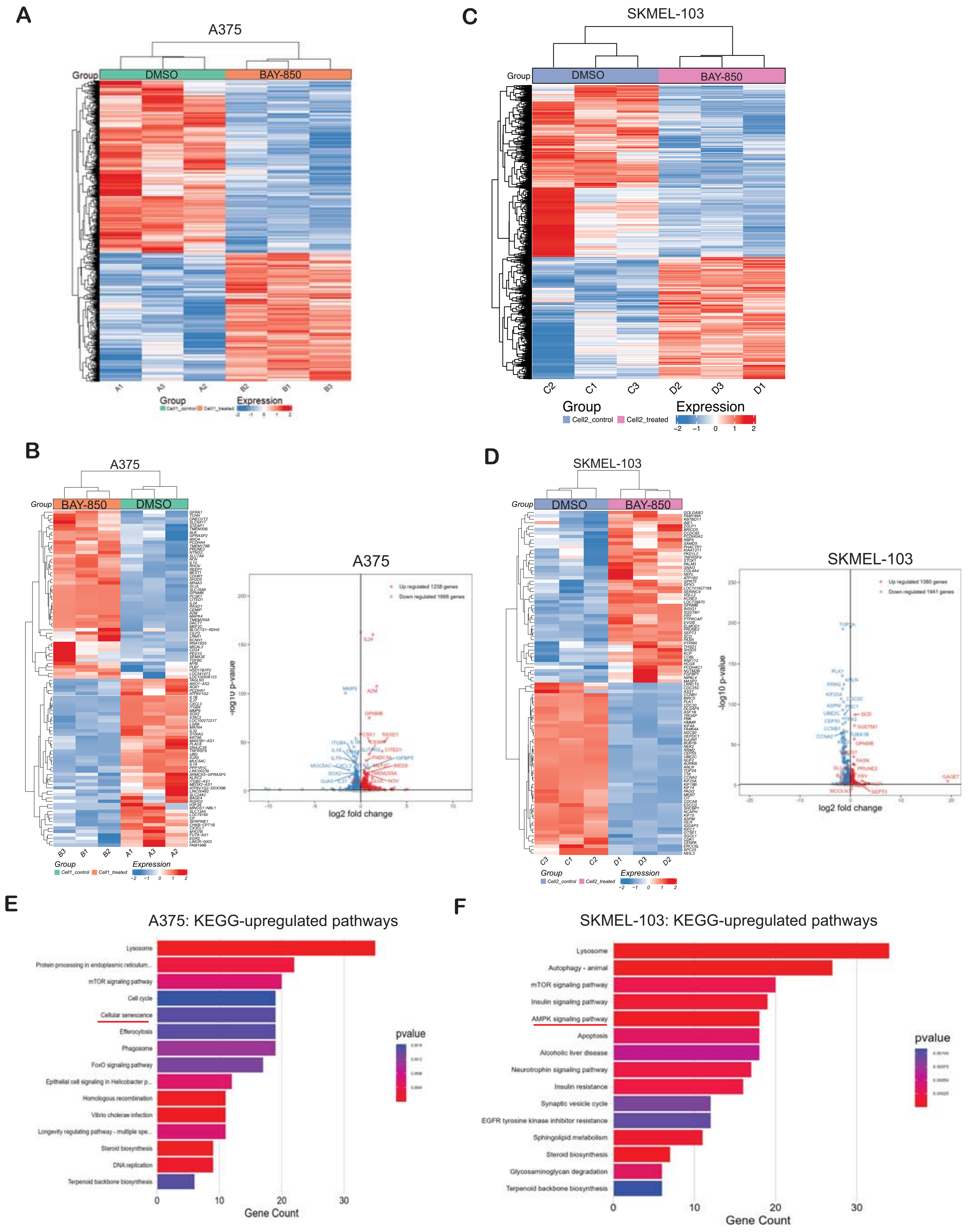
A
A375
Group
DMSO
BAY-850
A1 A3 A2 B2 B1 B3
Group
Expression
C
SKMEL-103
Group
DMSO
BAY-850
C2 C1 C3 D2 D3 D1
Group
Cell2_control
Cell2_treated
Expression
−2 −1 0 1 2
B
A375
Group
BAY-850
DMSO
B3 B1 B2 A1 A3 A2
Group
Cell1_control
Cell1_treated
Expression
A375
-log10 p-value
log2 fold change
D
SKMEL-103
Group
DMSO
BAY-850
C3 C1 C2 D1 D3 D2
Group
Cell2_control
Cell2_treated
Expression
SKMEL-103
-log10 p-value
log2 fold change
E
A375: KEGG-upregulated pathways
Lysosome
Protein processing in endoplasmic reticulum...
mTOR signaling pathway
Cell cycle
Cellular senescence
Efferocytosis
Phagosome
FoxO signaling pathway
Epithelial cell signaling in Helicobacter p...
Homologous recombination
Vibrio cholerae infection
Longevity regulating pathway - multiple spe...
Steroid biosynthesis
DNA replication
Terpenoid backbone biosynthesis
0 10 20 30
Gene Count
pvalue
F
SKMEL-103: KEGG-upregulated pathways
Lysosome
Autophagy - animal
mTOR signaling pathway
Insulin signaling pathway
AMPK signaling pathway
Apoptosis
Alcoholic liver disease
Neurotrophin signaling pathway
Insulin resistance
Synaptic vesicle cycle
EGFR tyrosine kinase inhibitor resistance
Sphingolipid metabolism
Steroid biosynthesis
Glycosaminoglycan degradation
Terpenoid backbone biosynthesis
0 10 20 30
Gene Count
pvalue

Figure EV3. ATAD2 targeting activates tumor growth inhibitory pathways in melanoma cells.

(**A**, **C**) Heatmap showing the genes that are upregulated and downregulated in A375 and SKMEL-103 cells upon treatment with BAY-850 (5 μM) for 48 h compared with the DMSO-treatment. (**B**, **D**) Heatmap showing top 50 upregulated and 50 downregulated genes (left) and volcano plot with top 15 upregulated and 15 downregulated genes (right) in A375 and SKMEL-103 cells upon BAY-850 treatment. (**E**, **F**) Pathways that were significantly upregulated upon BAY-850 treatment in A375 and SKMEL-103 cells based on gene expression changes was analyzed using KEGG pathway enrichment analysis and top 15 significantly altered pathway based on gene ratio and *P* values is presented. (**A**–**D**) *P* value was calculated using Quasi-Likelihood (QL) F-Tests.

 © The Author(s)

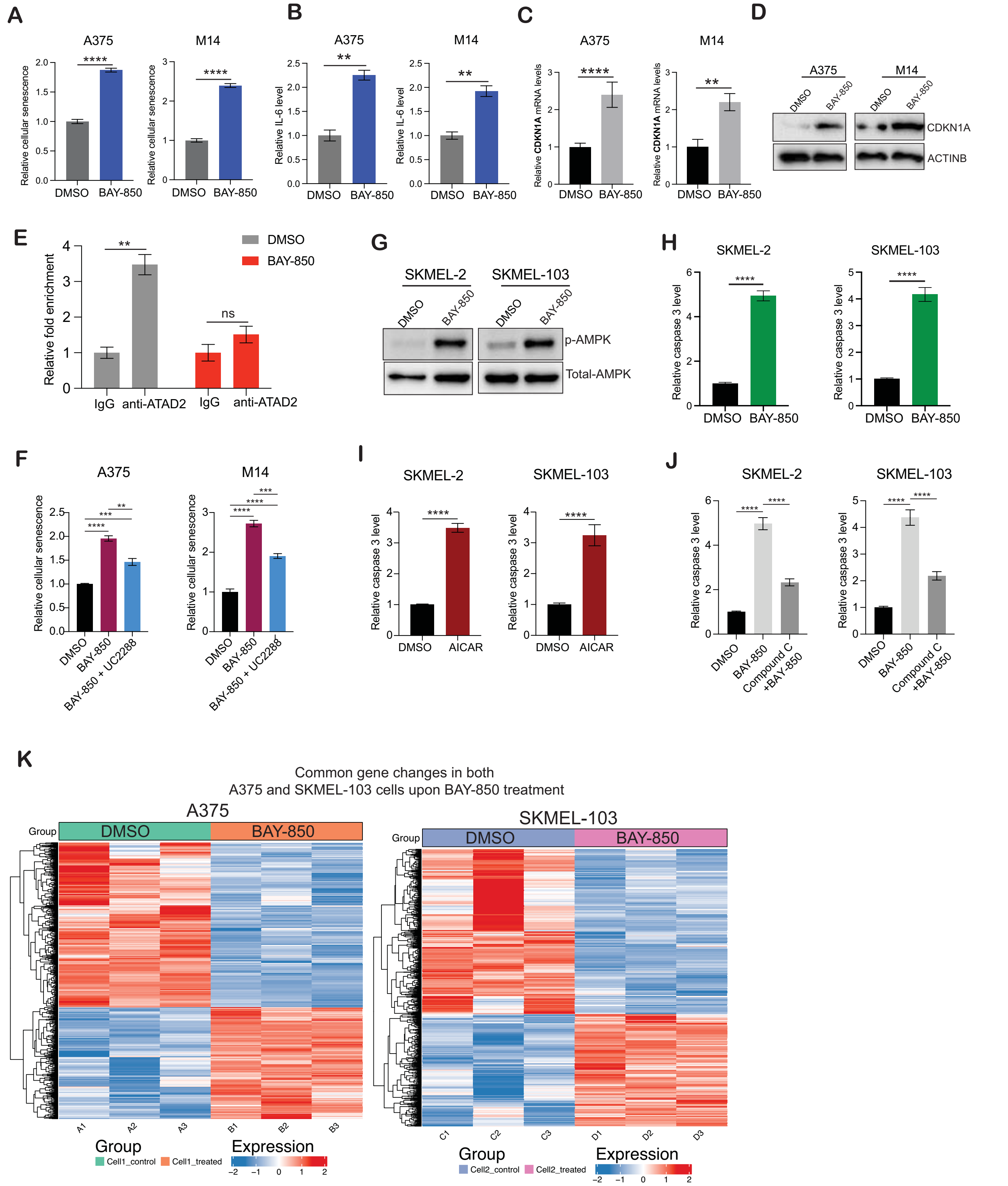
A
A375
M14
Relative cellular senescence
****
DMSO BAY-850
B
Relative IL-6 level
**
C
Relative CDKN1A mRNA levels
D
DMSO BAY-850
CDKN1A
ACTINB
E
Relative fold enrichment
DMSO
BAY-850
ns
IgG anti-ATAD2 IgG anti-ATAD2
G
SKMEL-2
SKMEL-103
p-AMPK
Total-AMPK
H
Relative caspase 3 level
F
BAY-850 + UC2288
***
I
AICAR
J
Compound C +BAY-850
K
Common gene changes in both
A375 and SKMEL-103 cells upon BAY-850 treatment
Group
A1 A2 A3 B1 B2 B3
C1 C2 C3 D1 D2 D3
Expression
Cell1_control Cell1_treated
Cell2_control Cell2_treated
−2 −1 0 1 2

Figure EV4. ATAD2 inhibits the growth of melanoma cells by regulating various tumor growth inhibitory pathways.

(**A**) Senescence associated beta gal assay was performed in indicated melanoma cell lines upon treatment with either DMSO or 5 μM BAY-850 for 48 h. Relative cellular senescence in BAY-850 treated condition with respect to DMSO-treated condition is presented for the indicated melanoma cell lines. ****P = < 0.0001, ****P = < 0.0001, from left to right. (**B**) Senescence associated secretory factor IL-6 level was measured in indicated melanoma cell lines upon treatment with either DMSO or 5 μM BAY-850 for 48 h using ELISA based method. Relative IL6 level in BAY-850 treated condition with respect to DMSO treatment for each cell line is presented. **P = 0.0012, **P = 0.0024, from left to right. (**C**, **D**) The indicated melanoma cell lines (A375 and M14) were treated with either DMSO or ATAD2 inhibitor BAY-850 5 μM for 48 h. (**C**) *CDKN1A (p21)* mRNA expression was measured using RT-qPCR and plotted as the level in BAY-850-treated cells relative to that in DMSO-treated cells. *ACTINB* was used as a normalization control. ****P = < 0.0001, **P = 0.0018, from left to right. (**D**) p21 protein expression was measured using western blot analysis under the indicated conditions. ACTINB was used as a loading control. (**E**) CUT-&-RUN analysis of ATAD2 binding on *CDKN1A* promoter in A375 treated with either DMSO or BAY-850 was performed. IgG was used as a negative control for CUT-&-RUN, and fold-enrichment plotted relative to IgG is shown. **P = 0.0016, ns P = 0.1951, from left to right. (**F**) The indicated melanoma cell lines were treated with either DMSO or 5 μM BAY-850 alone or 5 μM UC2288 and 5 μM BAY-850 in combination for 48 h, and cellular senescence was measured quantitatively using senescence-β-galactosidase activity assay kit (fluorescence, plate based # 25833; cell signaling) following the manufacturer's protocol and plotted. Relative cellular senescence in single and combination treatment condition with respect to DMSO control treated condition is plotted. ****P = < 0.0001, ***P = 0.0007, **P = 0.0017, ****P = < 0.0001, ****P = < 0.0001, ***P = 0.0002, from left to right. (**G**, **H**) The indicated melanoma cell lines (SKMEL-2 and SKMEL-103) were treated with either DMSO or ATAD2 inhibitor BAY-850 5 μM for 48 h. (**G**) phospho-AMPK and total AMPK protein expression was measured using western blot analysis under the indicated conditions. (**H**) Apoptosis was measured under the indicated conditions, ****P = < 0.0001, ****P = < 0.0001, from left to right. (**I**) The indicated melanoma cell lines were treated with either DMSO or 1 mM AICAR for 48 h, and caspase 3 level was measured using Caspase 3 colorimetric assay kit (CASP3C, Sigma-Aldrich), following the manufacturer's protocol. Relative caspase 3 level in treatment condition with respect to DMSO control treated condition is plotted. ****P = < 0.0001, ****P = < 0.0001, from left to right. (**J**) The indicated melanoma cell lines were treated with either DMSO or 5 μM BAY-850 alone or 0.1 μM Compound C and 5 μM BAY-850 in combination for 48 h, and caspase 3 level was measured using Caspase 3 colorimetric assay kit (CASP3C, Sigma-Aldrich), following the manufacturer's protocol. Relative caspase 3 level in single and combination treatment condition with respect to DMSO control treated condition is plotted, ****P = < 0.0001, ****P = < 0.0001, ****P = < 0.0001, ****P = < 0.0001, from left to right. (**K**) Heatmap showing the common genes that are upregulated and downregulated in A375 and SKMEL-103 cells upon treatment with BAY-850 (5 μM) for 48 h compared with DMSO-treatment. (**A–C**, **E**, **F**, **H–J**) P value was calculated using unpaired Student's t test using three independent replicates.

 © The Author(s)

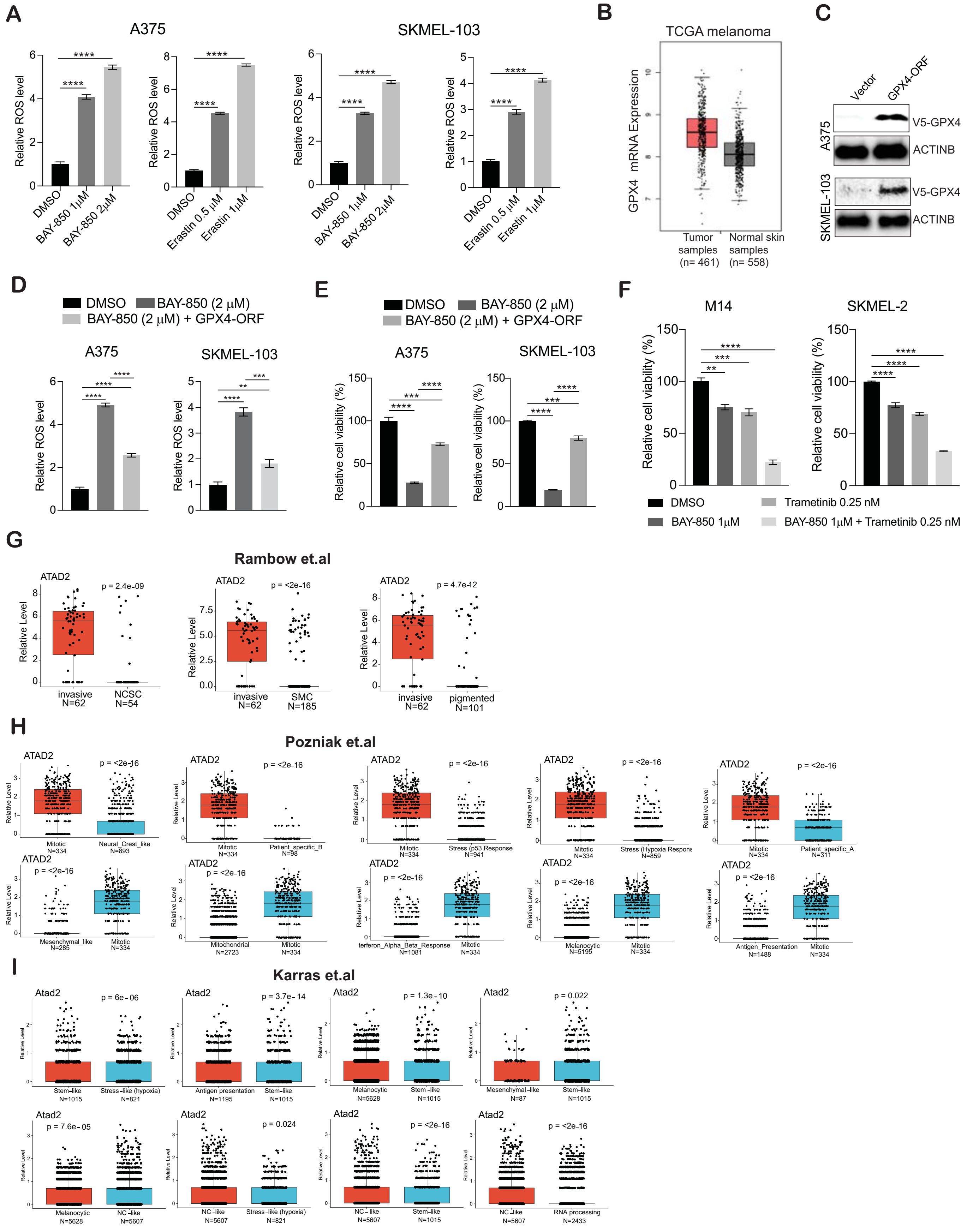
A
A375
SKMEL-103
Relative ROS level
DMSO
BAY-850 1μM
BAY-850 2μM
Erastin 0.5 μM
Erastin 1μM
B
TCGA melanoma
GPX4 mRNA Expression
Tumor samples (n= 461)
Normal skin samples (n= 558)
C
Vector
GPX4-ORF
A375
SKMEL-103
V5-GPX4
ACTINB
D
DMSO
BAY-850 (2 μM)
BAY-850 (2 μM) + GPX4-ORF
A375
SKMEL-103
Relative ROS level
E
DMSO
BAY-850 (2 μM)
BAY-850 (2 μM) + GPX4-ORF
A375
SKMEL-103
Relative cell viability (%)
F
M14
SKMEL-2
Relative cell viability (%)
DMSO
BAY-850 1μM
Trametinib 0.25 nM
BAY-850 1μM + Trametinib 0.25 nM
G
Rambow et.al
ATAD2
Relative Level
p = 2.4e-09
invasive N=62
NCSC N=54
p = <2e-16
SMC N=185
p = 4.7e-12
pigmented N=101
H
Pozniak et.al
ATAD2
Mitotic N=334
Neural_Crest_like N=893
Patient_specific_B N=98
Stress (p53 Response N=941
Stress (Hypoxia Respons N=859
Patient_specific_A N=311
Mesenchymal_like N=285
Mitochondrial N=2723
terferon_Alpha_Beta_Response N=1081
Melanocytic N=5195
Antigen_Presentation N=1488
p = <2e-16
I
Karras et.al
Atad2
p = 6e-06
Stem-like N=1015
Stress-like (hypoxia) N=821
p = 3.7e-14
Antigen presentation N=1195
p = 1.3e-10
Melanocytic N=5628
p = 0.022
Mesenchymal-like N=87
p = 7.6e-05
NC-like N=5607
p = 0.024
p = <2e-16
RNA processing N=2433

Figure EV5. ATAD2 targeting induces ferroptosis in melanoma cells and GPX4 overexpression partially rescues ATAD2 targeting induced ferroptosis induction and cell viability.

(**A**) The indicated melanoma cell lines were treated with either DMSO or BAY-850 or erastin at the shown concentration and cellular ROS levels was detected using the Cellular Reactive Oxygen Species Detection Assay Kit (ab186027, Abcam, Cambridge, UK), following the manufacturer's protocol. Relative ROS in BAY-850 or erastin treated condition or with respect to DMSO control treated condition is plotted. ****$P = < 0.0001$, ****$P = < 0.0001$, ****$P = < 0.0001$, ****$P = < 0.0001$, ****$P = < 0.0001$, ****$P = < 0.0001$, ****$P = < 0.0001$, ****$P = < 0.0001$, from left to right. (**B**) *GPX4* mRNA expression was plotted using Gene Expression Profiling Interactive Analysis (GEPIA). (**C**) Indicated melanoma cell lines overexpressing either vector control or GPX4-ORF were immune-blotted for the shown proteins confirming GPX4 overexpression. (**D**) Indicated melanoma cell lines overexpressing either vector control or GPX4-ORF were treated with BAY-850 at the shown concentration and cellular ROS levels was detected using the Cellular Reactive Oxygen Species Detection Assay Kit (ab186027, Abcam, Cambridge, UK), following the manufacturer's protocol. Relative ROS in the shown condition is plotted, ****$P = < 0.0001$, ****$P = < 0.0001$, ****$P = < 0.0001$, ****$P = < 0.0001$, **$P = 0.0046$, ***$P = 0.0001$, from left to right. (**E**). Indicated melanoma cell lines overexpressing either vector control or GPX4-ORF were treated with BAY-850 at the shown concentration and cell viability was assessed by 3-(4,5-dimethylthiazol-2-yl)-2,5-diphenyltetrazolium bromide (MTT) assay. Relative percentage cell viability in the shown condition is presented, ****$P = < 0.0001$, ***$P = 0.0008$, ****$P = < 0.0001$, ****$P = < 0.0001$, ***$P = 0.0004$, ****$P = < 0.0001$, from left to right. (**F**) The indicated melanoma cell lines were treated with either DMSO or BAY-850 1 μM alone or trametinib 0.25 nM alone or combination of BAY-850 1 μM and trametinib 0.25 nM for 3 days, and viability was assessed by 3-(4,5-dimethylthiazol-2-yl)-2,5-diphenyltetrazolium bromide (MTT) assay. Relative percentage cell viability in the shown condition is presented, **$P = 0.0011$, ***$P = 0.0007$, ****$P = < 0.0001$, ****$P = < 0.0001$, ****$P = < 0.0001$, ****$P = < 0.0001$, from left to right. (**G–I**) Analysis of the Rambow et al, (**G**), Pozniak et al, (**H**), and Karras et al, (**I**) datasets. N represents cell number. (**A**, **D**, **E**, **F**) *P* value was calculated using unpaired Student's *t* test using three independent replicates. (**G–I**) *P* value was calculated using Kruskal–Wallis test.

© The Author(s)
